# Duration between rewards controls the rate of behavioral and dopaminergic learning

**DOI:** 10.1038/s41593-026-02206-2

**Published:** 2026-02-12

**Authors:** Dennis A. Burke, Annie Taylor, Huijeong Jeong, SeulAh Lee, Leo Zsembik, Brenda Wu, Joseph R. Floeder, Gautam A. Naik, Ritchie Chen, Vijay Mohan K Namboodiri

**Affiliations:** 1 Department of Neurology, University of California, San Francisco, San Francisco, CA USA; 2 Neuroscience Graduate Program, University of California, San Francisco, San Francisco, CA USA; 3 University of California, Berkeley, Berkeley, CA USA; 4 Department of Neurological Surgery, Department of Psychiatry and Behavioral Sciences, Department of Bioengineering and Therapeutic Sciences, University of California, San Francisco, San Francisco, CA USA; 5 Weill Institute for Neurosciences, University of California, San Francisco, San Francisco, CA USA; 6 Kavli Institute for Fundamental Neuroscience, Center for Integrative Neuroscience, University of California, San Francisco, San Francisco, CA USA

**Keywords:** Reward, Classical conditioning

## Abstract

Learning the causes of rewards is crucial for survival. Cue–reward associative learning is controlled in the brain by mesolimbic dopamine. It is widely believed that dopamine drives learning by conveying a reward prediction error. Dopamine-based learning algorithms are generally ‘trial-based’: learning progresses sequentially across individual cue–outcome experiences. A foundational assumption of these models is that the more cue–reward pairings one experiences over a fixed duration, the more one learns this association. By identifying a new biological principle governing learning, we disprove this assumption. Specifically, across many conditions in mice, we show that behavioral and dopaminergic learning rates are proportional to the duration between rewards (or punishments). Due to this rule, the overall learning over a fixed duration is independent of the number of cue–outcome experiences. A dopamine-based model of retrospective learning explains these findings, thereby providing a unified account of the biological mechanisms of learning.

## Abstract (web summary)

Cue–reward learning rate scales proportionally with the time between rewards. Consequently, learning over a fixed duration is independent of the number of trials. This challenges trial-based dopamine learning models but supports retrospective learning.

## Main

The neurobiological study of reward learning is dominated by the hypothesis that mesolimbic dopamine conveys a reward prediction error (RPE)^1^ and that the brain implements temporal difference reinforcement learning (TDRL)^2^. TDRL has been influential in explaining behavior and dopamine dynamics across cue–reward learning^1–9^. TDRL implementations assume that the learning rate, which determines the amount learned per cue–reward experience, is a free parameter in deterministic environments (although modulated by uncertainty in nondeterministic ones^10–12^). Thus, the dominant framework for dopamine-based learning does not anticipate any mathematical rules governing learning rate in simple deterministic cue–reward learning.

However, learning is known to be more effective per experience with greater temporal spacing (‘spacing effect’^13,14^, for example, students are regularly advised that studying over time is more effective than ‘cramming’^15^). This effect is widely recognized^14,16–19^, including in cue–reward learning^20–25^. Determining whether spacing effects in cue–reward learning depend on duration between cues, duration between rewards or some combination thereof could reveal fundamental algorithms of learning. Yet, most demonstrations of spacing effects do not consider or propose a fundamental rule governing learning rate. In contrast, a meta-analysis has suggested that learning rate is proportional to the ratio of the spacing between consecutive cue–reward experiences to the interval between cue and reward^26–28^. However, the generality of this rule is debated^29–33^.

Why is discovering such a scaling rule governing learning rate important? For one, it would instruct principles to optimize learning from each experience. Further, quantitative empirical rules have enabled some of the greatest discoveries (for example, the mechanisms governing gravity, genetic inheritance and action potential generation). Yet, despite their potential to reveal mechanisms, scaling rules of learning rate have largely been ignored in neurobiological conceptions of cue–reward learning, which have instead primarily focused on explaining mesolimbic dopamine dynamics^1,4,6–8,34–37^. However, a fundamental mechanism of cue–reward learning should capture not only dopamine dynamics, but also quantitative rules of learning rate control.

To address this major gap, we investigated the behavioral, algorithmic and dopaminergic mechanisms underlying learning rate control. We tested the impact of trial spacing on learning rate during cue–reward trace conditioning, the dominant paradigm used to study dopamine function^1,6^, in which scaling rules of learning rate remain untested. In addition to behavioral learning, we measured the evolution of mesolimbic dopaminergic cue responses across conditioning (‘dopaminergic learning’). Surprisingly, we find a strong, mathematically proportional relationship between both behavioral and dopaminergic learning rates and the duration between rewards (‘inter-reward interval’ or IRI)—a rule different from that suggested by prior work^26,27^. A model of retrospective learning triggered by rewards (that is, learning whether a cue precedes reward) that accounts for known mesolimbic dopaminergic dynamics^35^ naturally explains the scaling of behavioral and dopaminergic learning rates by IRI.

## Behavioral learning in one-tenth the experiences with ten times the trial spacing

**Fig. 1:**
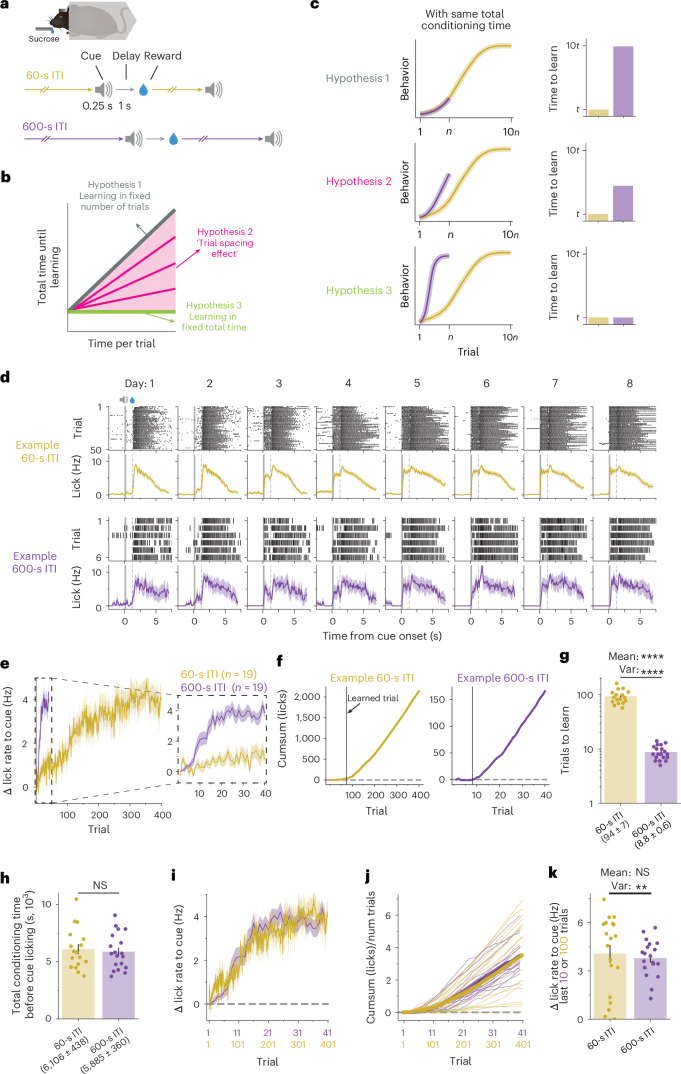
Behavioral learning in one-tenth the experiences with ten times the trial spacing. **a**, Schematic of experimental setup. Head-fixed mice underwent identical cue–reward pairing trials, differing only in the duration from reward to next cue (that is, the ITI). Mice were conditioned for 1 h per day resulting in 50 (60-s ITI) or 6 (600-s ITI) trials per session (Methods). **b**, Schematic illustrating three hypotheses: no trial spacing effect, a qualitative trial spacing effect and a quantitative proportional relationship between trial spacing and per-trial learning rate. **c**, Predicted group-averaged learning curves for 60-s and 600-s ITIs under each hypothesis, showing the expected relationship between learning rate and total conditioning time. **d**, Example lick raster plots (upper row) and lick peri-stimulus time histograms (PSTHs; lower row) for one example mouse from either the 60-s ITI group (top, gold) or the 600-s ITI group (bottom, purple) showing every cue and reward presentation across 8 days of conditioning. Each column represents a single day of conditioning. Graphs are aligned to cue onset (cue duration denoted by gray shading). Reward delivery is denoted by the vertical dashed line. **e**, 600-s ITI mice learn and reach asymptotic behavior in fewer trials than 60-s ITI mice. Left, Time course of mean cue-evoked licking over 40 (600-s ITI, purple, *n* = 19 mice) or 400 (60-s ITI, gold, *n* = 19 mice) cue–reward presentations. Inset right, Zoom of first 40 trials. Lines represent the mean across animals and the shaded area represents the s.e.m. **f**, Cumulative sum (cumsum) of cue-evoked licks across trials from the same example mice (**d**), which is used to determine when mice show evidence of learning (‘learned trial’; Extended Data Fig. 2 and Methods). The example 60-s ITI mouse learns after trial 74; the 600-s ITI mouse learns after trial 8. The learned trial is denoted by the vertical line. **g**, 600-s ITI mice learn in ten times fewer trials than 60-s ITI mice. Bar height represents mean learned trial for 60-s (*n* = 17) and 600-s (*n* = 19) ITI mice, plotted on a log scale. Error bar represents the s.e.m. Circles represent individual mice. Values under labels represent the mean ± s.e.m. Two mice that did not show evidence of learning were excluded (Extended Data Fig. 2 and Methods). ****P < 0.0001, Welch’s *t*-test, *F*-test. **h**, 60-s ITI and 600-s ITI mice learn after the same total conditioning time. Bar height represents mean total conditioning time until learned trial for 60-s (*n* = 17) and 600-s (*n* = 19) ITI groups. Error bar represents the s.e.m. Circles represent individual mice. Values under labels represent the mean ± s.e.m. NS, not significant; Welch’s *t*-test. **i**,**j**, On average, learning between groups progresses similarly as a function of total conditioning time and thus scales with the ratio of ITIs. **i**, Average cue-evoked licking for 600-s ITI and 60-s ITI groups across scaled trials (same data as **e**), showing that the 600-s ITI group learns ten times more per experience compared to the 60-s ITI group. **j**, Cumsum of cue-evoked licks plotted on the same scaled *x* axis. Thick lines represent means, and thin lines represent individual animals. Note the higher variability in the 60-s ITI group (quantified in **k**). **k**, Asymptotic cue-evoked licking have similar group means, but different variances. Bars represent mean cue-evoked licking during trials 301–400 (60-s ITI) or trials 31–40 (600-s ITI). Error bars represent the s.e.m., and circles represent individual mice. Welch’s *t*-test; **P < 0.01, *F*-test. See Supplementary Table 1 for full statistical details for all figures (tests, *n* values, degrees of freedom and corrected/uncorrected *P* values). Error bars and shading represent the s.e.m. unless otherwise noted. Values displayed under bar graph labels represent the mean ± s.e.m.

Most demonstrations of the trial spacing effect with cue–reward learning compare ‘spaced’ trials with standard intertrial intervals (ITIs) to ‘massed’ trials with a short ITI relative to cue–reward delay^20,24^ (ITI:cue–reward delay ratio < 10). Here, we asked whether trial spacing effects persist when ITI is much longer than cue–reward delay, and if so, whether there are strong, mathematical rules underlying learning rate. Thus, we classically conditioned thirsty head-fixed mice to associate a brief auditory tone (0.25 s, 12 kHz) (conditioned stimulus, CS) with the delivery of sucrose reward (15% wt/vol, 2–3 µl) in front of their mouth (Fig. 1a). Two groups of mice were presented with this same trial structure, with one group experiencing 60-s ITIs (‘60-s ITI’ mice) and another group experiencing 600-s ITIs (‘600-s ITI’ mice). Both groups were trained for ~1 h per day. So, 60-s ITI mice were presented with 50 cue–reward pairings a day, while 600-s ITI mice were presented with 6 cue–reward pairings a day (accounting for trial and fixed reward consumption periods; Methods). By placing mice a fixed distance from the reward spout, the head-fixed preparation both allows for brief cues critical for testing long ITIs relative to trial period, and enables conditioning without reward pretraining, thereby preventing nonspecific learning.

We tested between three hypotheses (Fig. 1b,c). Hypothesis 1 predicts that beyond an ITI already much longer than the cue–reward delay (as in 60-s ITI mice, ITI:cue–reward delay = 48), there is no spacing effect, and trial-by-trial learning rate is equivalent between groups. Thus, the number of trials to learn is constant and the total conditioning time before emergence of learned behavior increases in direct proportion to the ITI. Hypothesis 2 predicts that trial spacing enhances learning rate past 60-s ITI, so the number of trials to learn decreases with increasing ITI and the total time for learning still increases with increasing ITI but less steeply than in hypothesis 1. For instance, it may take 100 trials to learn with 60-s ITI but 90 trials to learn with 600-s ITI, consistent with trial spacing effect and an increase in the total time for learning. Hypothesis 3 predicts an extreme spacing effect with a proportional relationship between trial spacing and the learning rate per trial, consistent with learning rate scaling with IRI, or inter-cue interval (ICI), or the ratio between IRI and cue–reward delay^26–28^. This means that a tenfold increase in ITI (as well as IRI and ICI) reduces the number of experiences by a factor of 10 over a fixed duration but results in ten times more learning per experience, producing equivalent overall learning over a fixed duration. Thus, hypothesis 3 predicts that the total time until learning is a constant independent of the increasing ITI, implying that removing 9 of 10 experiences from 60-s ITI mice does not influence overall learning.

**Extended Data Fig. 1:**
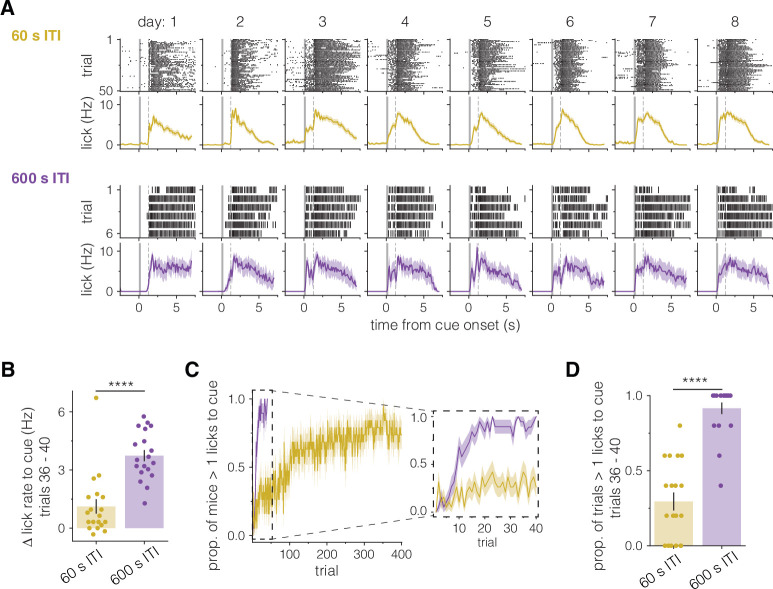
600 s ITI mice show evidence of learning in significantly fewer trials than 60 s ITI mice. **a**, Lick raster and PSTH plots from more individual 60 s ITI and 600 s ITI example mice, presented as in Fig. 1d. **b**, Average change in cue-evoked lick rate for trials 36 - 40 (timecourse in Fig. 1e). 600 s ITI mice show significantly more licking to cue in this period than 60 s ITI mice ****p < 0.0001, Welch’s t-test. **c-d**. 600 s ITI mice show asymptotic responding to the cue in fewer trials than 60 s ITI mice. **c**. *Left*, Timecourse showing the proportion of mice with more than one cue-evoked lick on each trial over 40 (600 s ITI, purple, n = 19) or 400 (60 s ITI, gold, n = 19) trials. *Inset, right*, Zoom in of first 40 trials for both groups. Lines represent mean across all animals and shaded area represents the SEM. **d**. Bar height represents proportion of trials in which animals responded to cue with more than one lick between trials 36 and 40. Error bars represent SEMs, and circles represent individual animals ****p < 0.0001, Welch’s t-test.

We measured behavioral learning using cue-evoked anticipatory licks before reward^3,6,38^. Mice from both groups began to show cue-evoked licking in the first few days of conditioning (Fig. 1d and Extended Data Fig. 1a). However, 600-s ITI mice learned and reached asymptotic behavior in far fewer trials than 60-s ITI mice (Fig. 1e). By trial 40, 600-s ITI mice showed significantly more cue-evoked licking (60-s ITI: 1.1 ± 0.4 Hz, 600-s ITI: 3.7 ± 0.3 Hz, *P* < 0.0001; Fig. 1e and Extended Data Fig. 1b) and were significantly more likely to respond to the cue (60-s ITI: 0.29 ± 0.06, 600-s ITI: 0.92 ± 0.04, *P* < 0.0001; Extended Data Fig. 1c,d) than 60-s ITI mice.

**Extended Data Fig. 2:**
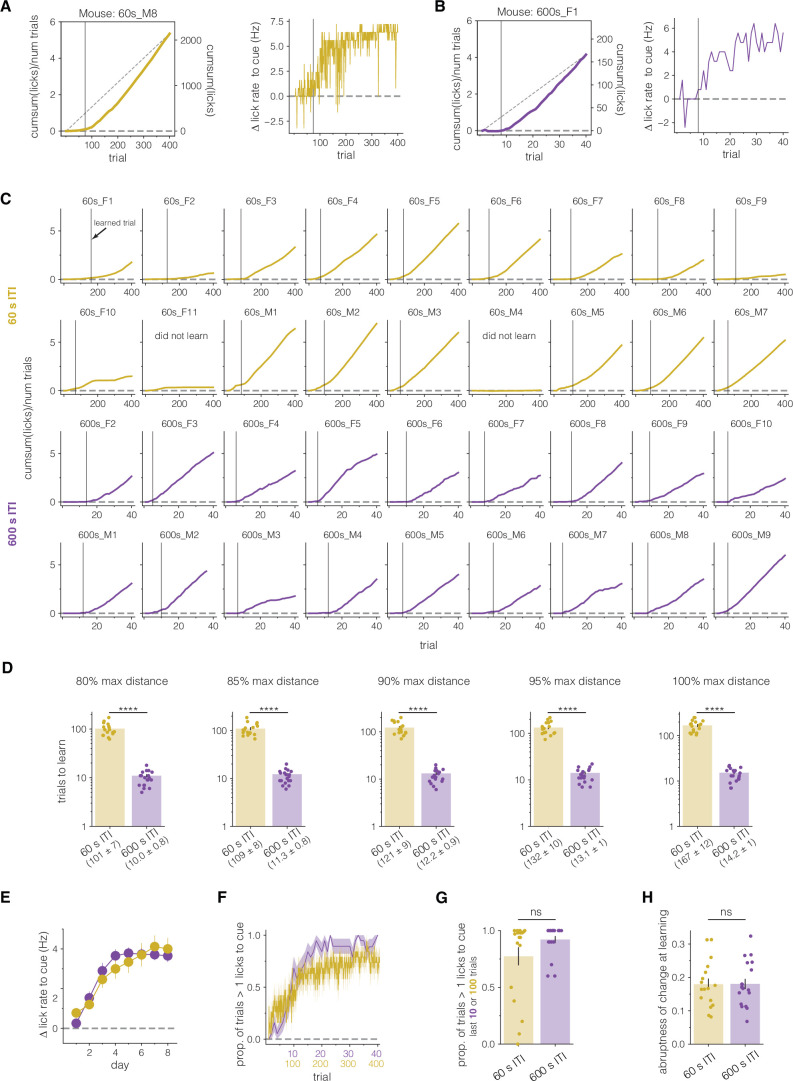
The cumulative sum of cue-evoked licking allows for determination of learned trial in individual animals (a-d), and learning scales with total conditioning time or the ratio between inter-trial intervals (e-h). **a-b**. Cumulative sum and trial-by-trial plots of cue-evoked licking of the same example mice as in Fig. 1d, f. *Left*, Cumulative sum plot showing diagonal line used to calculate learned trials. Right y-axis shows the total cumulative sum of all cue-evoked licks as in Fig. 1f. Left y-axis shows cumulative sum of cue-evoked licks divided by total trial number to allow comparisons across groups that experienced a different number of cue-reward pairings. Trial normalized cumulative sum values represent the mean number of cue-evoked licks over all previous trials. Solid vertical line represents the calculated learned trial, the trial after which animals show evidence of learning. *Right*, The cue-evoked change in lick rate plotted for the same individual example mouse. Note how the vertical line representing the learned trial, which corresponds to the point on cumulative sum plots where the cumulative sum curve takes off from the x-axis, captures the trial at which cue-evoked change in lick rate becomes consistently positive. **c.** Cumulative sum plots for all remaining mice included in behavior analysis plotted with trial normalized units (see **a,b**). Vertical line represents calculated learned trial. Animals which did not meet learning criteria (see *Methods*) are noted, and no vertical line is drawn. These animals were excluded from comparison of learned trials between groups. **d**. For analysis, learned trial was calculated as the first trial that fell within 75% of the maximum distance from a diagonal drawn from the point on the cumulative sum curve at trial 1 through trial 40 or 400 (Fig. 1g). 75% of maximum distance was chosen rather than the overall maximum distance (which would be the largest inflection point in the curve) to account for variability in post-learning behavior that occasionally caused the maximum distance from the diagonal to be at a point after a mouse has consistently licked to the cue for many trials. This choice did not affect our main conclusions as using 80%, 85%, 90%, 95%, or the maximum distance from the diagonal in our algorithm yielded a similar result of ten times more trials needed to learn in 60 s ITI mice than 600 s ITI mice. Bar heights represent mean number of trials to learn, error bars represent SEMs, and circles represent individual animals plotted on a log scale. Values under labels represent mean ± SEM. ****p < 0.0001, Welch’s t-test. **e**. Learning is similar between groups when trials are averaged across days. Timecourse of average cue-evoked lick rate as a function of days of conditioning. Circles represent mean change in lick rate per day, and error bars represent SEMs. **f-g**. Responding to cue scales with total conditioning time between groups and is not different at the end of conditioning. **f**. Timecourse showing the proportion of mice with more than one cue-evoked lick on each trial (same data as Extended Data Fig. 1c) plotted on scaled x-axis units. Lines represent means per group and shading represents SEM. **g**. Bar height represents proportion of trials in which animals responded to cue with more than one lick between trials 301 – 400 (60 s ITI) or 31 – 40 (600 s ITI). Error bars represent SEMs, and circles represent individual animals. ns: not significant, Welch’s t-test. **h**. The abruptness of change, a measure of how quickly an animal’s behavior changes at learning, determined by the steepness of the lick behavior cumulative sum curve (see *Methods*), is not different between groups. Bar height represents mean abruptness of change parameter for each group. Error bars represent SEMs, and circles represent individual animals. ns: not significant, Welch’s t-test.

To compare learning rates between groups, we determined the trial after which each mouse showed evidence of learning using the cumsum of cue-evoked licks (Methods, Fig. 1f and Extended Data Fig. 2a–d). Remarkably, 600-s ITI mice learned in 8.8 ± 0.6 trials, significantly fewer than the 94 ± 7 trials needed for 60-s ITI mice (*P* < 0.0001; Fig. 1g). By lengthening the ITI by a factor of 10, cue–reward learning required ten times fewer trials, showing a proportional scaling relationship between trial spacing and per-trial learning. This scalar relationship was not just limited to the learned trial number, as a single trial for 600-s ITI mice was worth 10 trials for 60-s ITI mice throughout learning (Fig. 1h–j and Extended Data Fig. 2e,f). Because 600-s ITI mice have the same experience as 60-s ITI mice but with the removal of 9 of 10 trials (that is, ten times the ITI), the overlapping learning curves demonstrate that those missing trials have no effect on total conditioning time until learning (*P* = 0.70; Fig. 1h), consistent with hypothesis 3 (Fig. 1b,c).

Further suggesting that learning between groups was simply scaled, average asymptotic cue-evoked licking (60-s ITI: 4.06 ± 0.54 Hz, 600-s ITI: 3.80 ± 0.26 Hz, *P* = 0.66; Fig. 1k), the likelihood of responses to the cue (60-s ITI: 0.77 ± 0.08, 600-s ITI: 0.92 ± 0.03, *P* = 0.098; Extended Data Fig. 2g), and the abruptness of change (60-s ITI: 0.18 ± 0.02, 600-s ITI: 0.18 ± 0.02, *P* = 0.97; Extended Data Fig. 2h), were all similar between groups. However, despite similar average rates of asymptotic licking, 60-s ITI mice showed significantly more variance in individual licking (*P* < 0.01; Fig. 1j,k; two 60-s ITI mice did not learn the cue–reward association; Extended Data Fig. 2c). An increased variance in the number of trials to learn was also seen in mice that showed evidence of learning (*P* < 0.0001; Fig. 1g). This suggests that individual variability in learning is driven in part by environmental factors and is not just a reflection of innate abilities.

## Dopaminergic learning in one-tenth the experiences with ten times the trial spacing

**Fig. 2:**
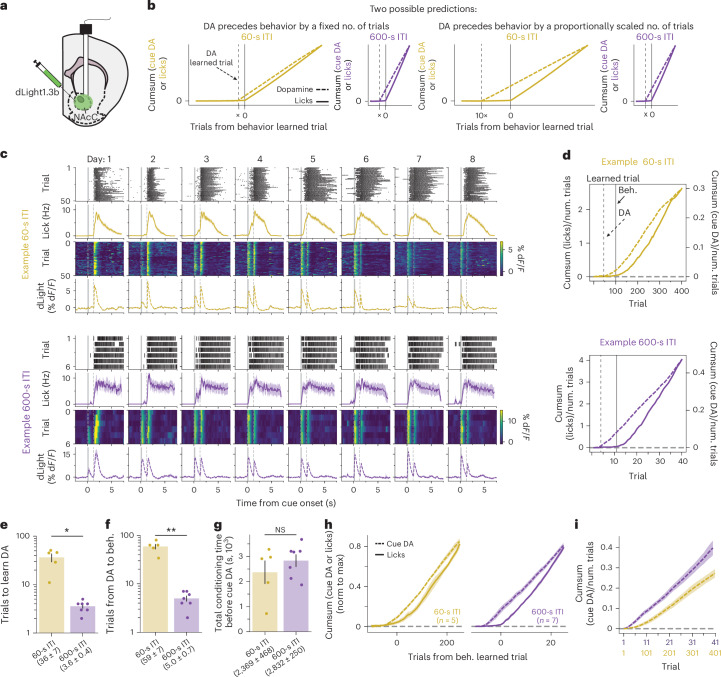
Dopaminergic learning in one-tenth the experiences with ten times the trial spacing. **a**, Schematic of mesolimbic dopamine measurements from the nucleus accumbens core (NAcC; Methods). **b**, Diagrams of two possible relationships between dopaminergic and behavioral learning in 60-s and 600-s ITI mice. **c**, Example lick raster plots (upper row), lick PSTHs (2nd row), heat maps of dopamine responses on each trial (3rd row) and average dopamine PSTHs for the day (lower row) for one example mouse from either the 60-s ITI group (top, gold) or the 600-s ITI group (bottom, purple) showing every cue and reward presentation across 8 days of conditioning. Lick data are presented as in Fig. 1d. Dopamine signals plotted as percentage change in fluorescence (%d*F/F*). Graphs are aligned to cue onset (cue duration denoted by gray shading). Reward delivery is denoted by the vertical gray dashed line. **d**, Cumsum of cue-evoked licks (solid, left axis) or of cue-evoked dopamine (dashed, right axis) for the same example mice as in **c**. Both lick and cue-evoked dopamine values were divided by total trial number to display average responses across conditioning. Before taking the cumsum, cue-evoked dopamine responses were normalized by maximum reward responses for each animal (Methods). Cumsum curves were used to determine the trial after which cue-evoked dopamine or cue-evoked licking emerge (‘learned trial’; Methods). Solid vertical lines represent a learned behavior trial, and dashed vertical lines represent a dopamine learned trial. This convention is followed for all similar figures in this paper. **e**, Dopamine responses to cue develop in ten times fewer trials in 600-s ITI mice (right, purple, *n* = 7) compared to 60-s ITI mice (left, gold, *n* = 5). Values under labels represent the mean ± s.e.m. across mice. *P < 0.05, Welch’s *t*-test. **f**, On average, dopamine cue responses develop 59 trials before the emergence of cue-evoked licking in 60-s ITI mice and 5 trials before in 600-s ITI mice. **P < 0.01. Welch’s *t*-test. **g**, Total conditioning time until the emergence of dopamine responses to cue for 60-s ITI and 600-s ITI mice. Error bar represents the s.e.m. Circles represent individual mice. Welch’s *t*-test. **h**, Average cumsum of cue-evoked licks (solid) and dopamine (dashed) across groups. Data were normalized by each animal’s final trial of conditioning and aligned to their learned trial before averaging. Lines represent means, and shading represents the s.e.m. **i**, Average cumsum of normalized cue-evoked dopamine responses in 60-s ITI (gold) and 600-s ITI (purple) mice. Cumsum curves divided by number of trials to account for differences between groups. DA, dopamine.

The dominance of trial-based accounts of associative learning is largely supported by the concordance between mesolimbic dopamine dynamics and TDRL RPE^1–8^. In such models, RPE/dopamine updates the value of a cue to drive behavior. Thus, cue-evoked dopamine should be tightly coupled to behavior^1,3^. To understand how learning rate scaling fits with dopamine, we examined how cue-evoked dopamine release evolves over learning (‘dopaminergic learning’) in both 60-s and 600-s ITI mice (Fig. 2). We considered two possible relationships between dopaminergic and behavioral learning (Fig. 2b). Either the emergence of cue-evoked dopamine precedes behavior by a fixed number of trials in both groups, or cue-evoked dopamine precedes behavior by ten times fewer experiences in 600-s ITI mice.

**Extended Data Fig. 3:**
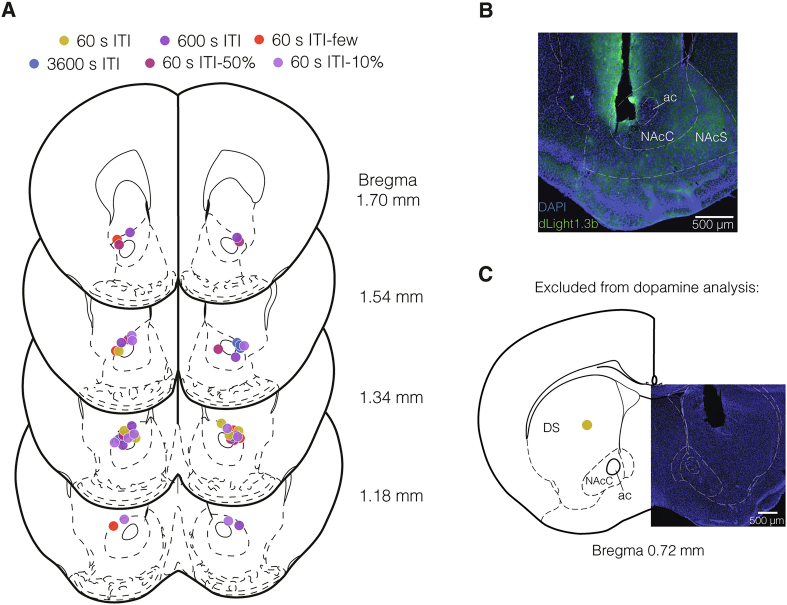
Fiber placements for dopamine measurement mice. **a.** Locations of center of optical fiber tip for subset of mice with fiber photometry dLight recordings from 60 s ITI (gold, n = 6), 600 s ITI (purple, n = 7), 3600 s ITI (blue, n = 5), 60 s ITI-few (red, n = 6), 60 s ITI-50% (magenta, n = 8), and 60 s ITI-10% (light purple, n = 10) groups. **b.** Example histology from a single mouse. Exact fiber locations of all cue-reward learning dopamine recording mice presented in **a**. Blue is DAPI staining and green is dLight1.3b. NAcC, nucleus accumbens core; NAcS, nucleus accumbens shell; ac, anterior commissure. **c.** Fiber location from the 1 mouse (60 s ITI group) out of 43 implanted cue-reward learning mice excluded from dopamine analysis for missed fiber placement.

**Extended Data Fig. 4:**
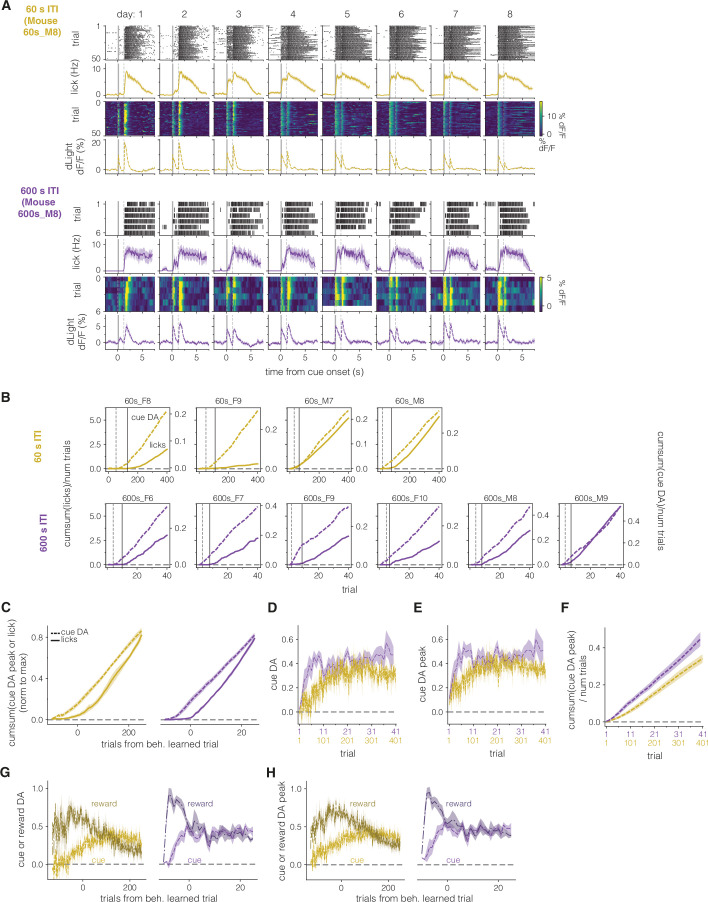
Taking the cumulative sum of cue-evoked dopamine and licking allows for determination of trials at which dopaminergic learning and behavioral learning occur in individual mice (a-b), and reward- and cue-evoked dopamine increase prior to behavioral learning (c-h). **a.** More lick raster, lick PSTH, dopamine response by trial, and average session dopamine PSTH plots from individual 60 s ITI and 600 s ITI example mice, presented as in Fig. 2c. **b.** Cumulative sum of cue-evoked licks (solid, left axis) or of normalized cue-evoked dopamine (dashed, right axis) for all 60 s and 600 s ITI dopamine recording mice not shown in Fig. 2d. Both lick and cue-evoked dopamine values were divided by total trial number to display average responses across conditioning. Solid vertical lines represent learned behavior trial and dashed vertical lines represent dopamine learned trial. **c**. Average cumulative sum of cue-evoked licks (solid) and dopamine response (dashed) for 60 s ITI (*left*, gold) and 600 s ITI (*right*, purple) mice. Data were normalized by each animal’s final trial of conditioning and aligned to their learned trial before averaging, as in Fig. 2h, but using measurements of the peak of the dopamine response rather than the AUC. Lines represent means, and shading represents SEM. **d-e**. Average normalized AUC (**d**) or peak (**e**) cue-evoked dopamine across scaled trial units. Lines represent means, and shading represents SEM. **f**. Average cumulative sum of normalized cue-evoked dopamine responses in 60 s ITI and 600 s ITI mice as in Fig. 2i but using measurements of the peak of the dopamine response instead of the AUC. Cumulative sum curves divided by number of trials to account for different trial numbers between groups. Lines represent means, and shading represents SEM. **g-h**. Average normalized AUC (**g**) or peak (**h**) of cue (lighter, dashed) and reward (darker, dashdot) evoked dopamine responses aligned to each animal’s behavior learned trial. Note the increase in reward response prior to behavioral learning. Lines represent means, and shading represents SEM.

To distinguish these possibilities, we measured dopamine release in the nucleus accumbens core with dLight1.3b in subsets of 60-s and 600-s ITI mice (Fig. 2a and Extended Data Fig. 3). In line with prior work^5,35,39,40^, cue-evoked dopamine release developed over trials and preceded the emergence of cue-evoked licking (Fig. 2c and Extended Data Fig. 4a). The trial at which cue-evoked dopamine emerged (Fig. 2d, Extended Data Fig. 4b and Methods) exhibited a proportional scaling between IRI and dopaminergic learning. In 600-s ITI mice, cue-evoked dopamine emerged after 3.6 ± 0.4 trials, significantly fewer than the 36 ± 7 trials needed for 60-s ITI mice (*P* < 0.05; Fig. 2e), and precedes behavior by 5.0 ± 0.7 trials, significantly fewer than the 59 ± 7 for 60-s ITI mice (*P* < 0.01; Fig. 2f). Thus, per-trial development of cue-evoked dopamine also scales proportionally with trial spacing and emerges in the same total conditioning time (*P* = 0.41; Fig. 2g). By increasing the ITI (and thus IRI and ICI) tenfold, cue-evoked dopamine both appears and precedes behavior by tenfold fewer trials (Fig. 2h and Extended Data Fig. 4c), consistent with hypothesis 3 (Fig. 1b,c). Despite the scaling in the onset of cue-evoked dopamine, it reached asymptotic levels more rapidly in 600-s versus 60-s ITI mice (Fig. 2i and Extended Data Fig. 4d–f). Further, reward-evoked dopamine increased during early conditioning as observed previously^35,36^, peaking before the onset of behavior (Extended Data Fig. 4g,h).

**Extended Data Fig. 5:**
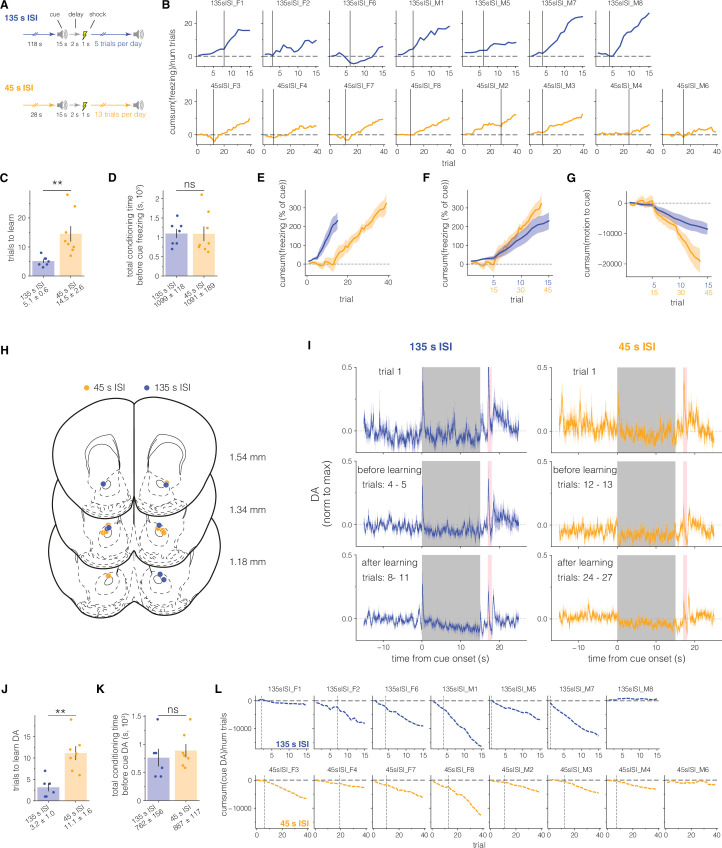
Learning rate scaled by inter-shock interval in cue-shock conditioned mice. **a.** Schematic of experimental setup for cue-shock conditioning (fear conditioning). Freely moving mice were divided into two groups that were each presented with identical cue-shock pairing trials (15 s cue, 2 s trace period, 1 s shock of 0.3 mA), but each group differed in the duration of the average inter-shock interval (ISI). 45 s ISI mice were conditioned with a 45 s ISI and 13 trials a day, while 135 s ISI mice were conditioned with a 135 s ISI and 5 shocks a day. Both groups were conditioned for 3 days. During conditioning, dopamine was recorded from all animals using dLight fiber photometry. (see *Methods*). **b.** Cumulative sum of cue-evoked freezing (% freezing to cue) as a function of trials for all individual fear conditioned mice. Learned trial is denoted by the solid black vertical line. **c.** Using same learned trial analysis as with cue-reward conditioned mice, 135 s ISI (blue, n = 7) mice learned in about one-third the number of trials as 45 s ISI mice (orange, n = 8). Bar height represents mean trial after which mice show evidence of learning. **p < 0.01, Welch’s t-test. **d.** Both 45 s and 135 s ISI mice learn in an equivalent amount of total conditioning time. ns: not significant, Welch’s t-test. **e-f**. Average cumulative sum of freezing during the cue for 45 s and 135 s ISI mice as a function of (**e**) unscaled and (**f**) scaled trials. **g**. Average cumulative sum of total motion during the cue as a function of scaled trials. Since the definition of freezing requires a threshold for lack of motion, we show the unthresholded average motion during the cue. **h**. Fiber locations for dLight photometry dopamine recordings. **i**. Average dopamine PSTHs for each group on trial 1 (*top*), prior to the onset of behavioral learning (freezing) (*middle*) and following behavioral learning (*bottom*). To average across animals, dopamine responses were normalized to the average of the largest three peaks recorded from cue onset through 2 s following shock termination (see *Methods*). Note that the mean response to the cue is a decrease in the level of dopamine that grows in magnitude over learning; the shock response is positive. This pattern of responses reproduces prior results^57^. **j.** Using the same dopamine learned trial analysis as with cue-reward conditioned mice to detect the onset of a decrease in dopamine during the cue, 135 s ISI (blue, n = 7) mice show cue responses in significantly fewer trials than 45 s ISI mice (orange, n = 8). **p < 0.01, Welch’s t-test. **k.** Both 45 s and 135 s ISI mice develop dopamine cue responses in an equivalent amount of total conditioning time. ns: not significant, Welch’s t-test. **l.** Cumulative sum of cue-evoked dopamine decreases as a function of trials for all individual fear conditioned mice. Dopamine learned trial is denoted by the dashed black vertical line. M8 and M6 were excluded from the analysis due to lack of consistent responses through the end of conditioning.

We further found that both behavioral and dopaminergic learning rate scaling occurs in aversive cue–shock learning (Extended Data Fig. 5). Specifically, animals that experienced three times longer inter-shock intervals (ISIs) acquired freezing in three times fewer cue–shock experiences. The total duration of conditioning until behavioral or dopaminergic learning did not differ between groups. These results imply that the scaling of learning rate by inter-outcome interval generalizes across appetitive and aversive learning.

## Learning rate scales proportionally with IRI

**Fig. 3:**
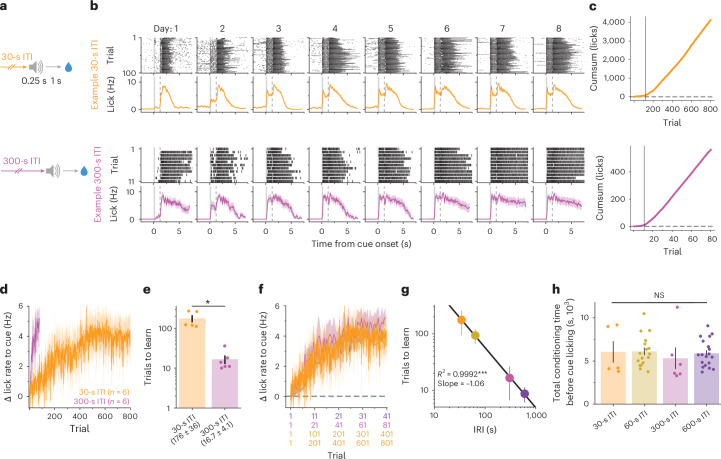
Learning rate scales proportionally with reward frequency across a range of trial spacing intervals. **a**, 30-s ITI and 300-s ITI conditioning (30 s, 100 trials per day, 300 s, 11 trials per day). **b**, Example lick raster plots (upper row) and PSTHs (lower row) for one example mouse from either 30-s ITI (top, orange) or 300-s ITI group (bottom, pink) showing every cue and reward presentation across 8 days of conditioning. Data are presented as in Fig. 1d. **c**, Cumsum of cue-evoked licks across trials from the same example mice as in **b**. Learned trial is denoted by the black vertical line. **d**, 300-s ITI mice learn and reach asymptotic behavior in fewer trials than 30-s ITI mice. Time course showing the average change in cue-evoked licking over 80 (300-s ITI, pink, *n* = 6) or 800 (30-s ITI, orange, *n* = 6) cue–reward presentations. **e**, 300-s ITI mice learn in ten times fewer trials than 30-s ITI mice. One mouse that did not show evidence of learning is excluded from comparison (Extended Data Fig. 6c and Methods). *P < 0.05, Welch’s *t*-test. **f**, Average cue-evoked licking for 30-s (orange, same data as **d**), 60-s (gold, same data as Fig. 1e,i), 300-s (pink, same data as **d**) and 600-s ITI mice (purple, same data as Fig. 1e,i) across scaled trial numbers. On average, learning between groups scales with the ratio of ITIs and progresses similarly as a function of total conditioning time. Lines represent mean across animals and shaded area represents the s.e.m. **g**, Mean trials to learn for different ITI groups as a function of IRI plotted on a log–log axis. Circles represent mean trials to learn per group, and error bars represent the s.d. Solid black line is the best-fit regression line (*R*^2^ = 0.9992, ***P < 0.001). Slope is not significantly different from −1 (one-sample *t*-test) indicating a proportional quantitative scaling relationship between IRI and learning rate (1/trials to learn). **h**, 30-s, 60-s, 300-s and 600-s ITIs show evidence of learning after the same total conditioning time. Welch’s analysis of variance (ANOVA).

**Extended Data Fig. 6:**
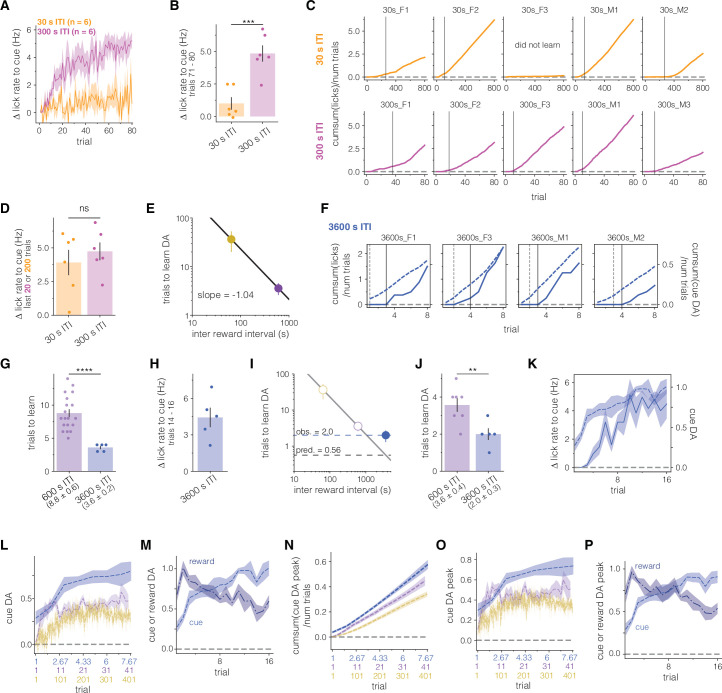
300 s ITI mice show evidence of learning in significantly fewer trials than 30 s ITI mice but do not differ in asymptotic behavior, and cue-evoked dopamine in 3600 s ITI mice appears in fewer trials and reaches a higher asymptote than in 600 s ITI mice. **a-b**. 300 s ITI mice show significantly more cue-evoked licking than 30 s ITI mice over the first 80 trials of conditioning. **a**. Timecourse of average cue-evoked lick rate across 80 trials. **b**. Mean change in cue-evoked lick rate for trials 71 – 80. ***p < 0.001, Welch’s t-test. **c.** Cumulative sum of cue-evoked licks for all 30 s and 300 s ITI mice not shown in Fig. 3c. Learned trial denoted by solid vertical line. **d.** Asymptotic cue-evoked lick rates are not significantly different between 30 s and 300 s ITI mice. Bars represent mean cue-evoked lick rates during trials 601–800 (30 s ITI) or trials 61-80 (300 s ITI). Error bars represent SEMs and circles represent individual mice. ns: not significant, Welch’s t-test. **e.** Mean trials to learn DA as a function of IRI for 60 s and 600 s ITI mice plotted on a log-log axis. Slope of line between points is −1.04, suggesting that dopaminergic learning scales proportionally with IRI, similar to behavioral learning. Error bars represent the standard deviation across mice. **f.** Cumulative sum of cue-evoked licks (solid, left axis) or of normalized cue-evoked dopamine (dashed, right axis) for all 3600 s ITI mice not shown in Fig. 4c. Both lick and cue-evoked dopamine values were divided by total trial number to display average responses across conditioning. Solid vertical lines represent learned behavior trial and dashed vertical lines represent dopamine learned trial. **g.** Mean trials to learn for 3600 s and 600 s (same data as Fig. 1g) ITI mice. 3600 s ITI mice learn in significantly fewer trials. ****p < 0.0001, Welch’s t-test. **h.** Asymptotic (trials 14-16) cue-evoked lick rate in 3600 s ITI mice. Bar height and error bar represent mean and SEM across animals. **i.** 3600 s ITI mice (blue circle, filled) take more trials to learn than predicted from the scaling relationship in **e**. White filled circles and line are same data as **e**. Dashed lines represent predicted and observed mean trials to learn for 3600 s ITI. **j.** Mean trials to learn cue-evoked dopamine for 3600 s and 600 s (same data as Fig. 2e) ITI mice. 3600 s ITI mice learn in significantly fewer trials. **p < 0.01, Welch’s t-test. **k.** Average cue-evoked lick rate and normalized cue-evoked dopamine across all eight days of conditioning in 3600 s ITI mice (n = 5). **l.** Average normalized cue-evoked dopamine in 3600 s (blue, n = 5), 600 s (purple, same data as Extended Data Fig. 4d), and 60 s (gold, same data as Extended Data Fig. 4d) mice across scaled trials. **m.** Average normalized cue (dashed, lighter) or reward (dashdot, darker) evoked dopamine on each trial across all eight days of conditioning in 3600 s ITI mice (n = 5). **n.** Average cumulative sum of normalized cue-dopamine as in Fig. 4i, analyzing peak of dopamine response rather than AUC. **o.** Average normalized cue-evoked dopamine as in **l**, analyzing peak rather than AUC. **p.** Average normalized cue- or reward- evoked dopamine as in **m**, analyzing peak rather than AUC.

To further probe the relationship between trial spacing and learning rate, we conditioned two additional groups with the same cue–reward delay as above separated by either a 30-s (‘30-s ITI’, 100 trials per day) or 300-s (‘300-s ITI’, 11 trials per day) ITI (Fig. 3a–c). The 300-s ITI mice learned and reached asymptotic behavior in many fewer trials than 30-s ITI mice (Fig. 3d and Extended Data Fig. 6a), showing significantly more cue-evoked licking by trial 80 (30 s: 1.0 ± 0.5 Hz, 300 s: 4.8 ± 0.6 Hz, *P* < 0.001; Extended Data Fig. 6b). Similarly to 600-s versus 60-s ITI mice, 300-s ITI mice learned in ten times fewer trials than 30-s ITI mice (300 s: 16.7 ± 4.1; 30 s: 176 ± 36, *P* < 0.05; Fig. 3e and Extended Data Fig. 6c), while showing comparable asymptotic licking (30 s: 3.9 ± 1 Hz, 300 s: 4.7 ± 0.7 Hz, *P* = 0.49; Extended Data Fig. 6d).

**Extended Data Fig. 7:**
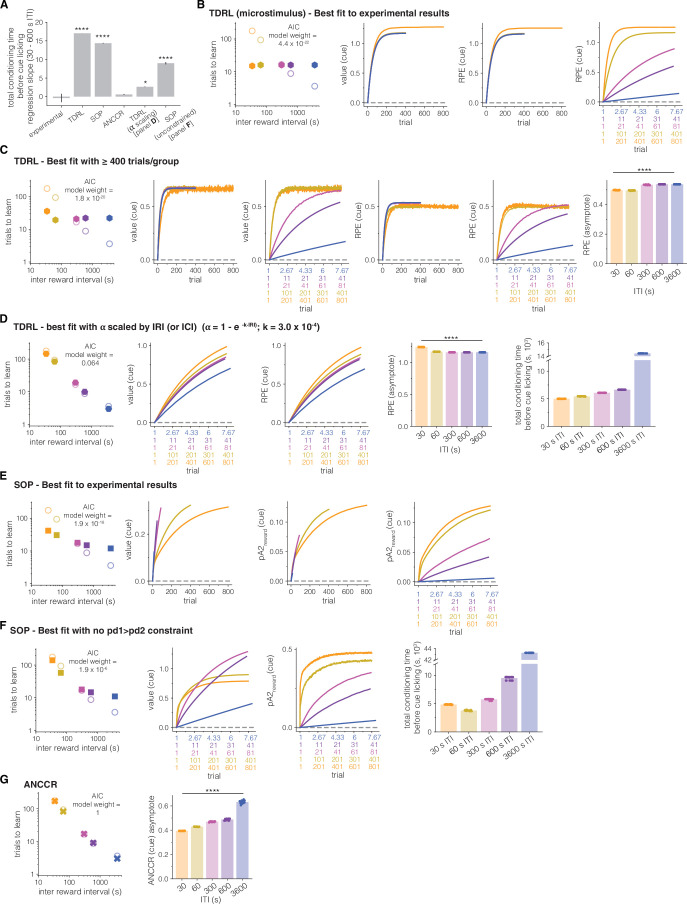
Total conditioning time and trials to learn comparisons between models, and additional simulation results. **a.** Slope of regressions for total time before cue licking as a function of ITI for 30 – 600 s ITI conditions from experimental results (Fig. 5b), TDRL (Fig. 5e), SOP (Fig. 5h), ANCCR (Fig. 5k), TDRL with alpha scaling (**d**), and SOP not constrained with pd1>pd2 (**f**). Error bars represent standard error of regression slope estimate. Asterisks represent significant difference from experimental results. **** p < 0.0001, * p < 0.05. t-test. **b.** Best fit TDRL model: *Left*, Trials to learn (when value > threshold) as a function of IRI for TDRL simulation with parameter combination that best fits experimental results (see *Methods*). Hexagons represent mean trials to learn across iterations (n = 20 each) for all conditions. Open circles represent experimental data (same as Figs. 3g and 4f). Akaike information criterion (AIC) model weight comes from comparison with best fit SOP(**e**) and ANCCR (**g**) models. Please note that this AIC model does not penalize free parameters, that is, is a conservative estimate biased against ANCCR (see *Methods* for rationale and Supplementary Table 1 for AIC model weight when penalizing for parameters). *Middle*-*left*, Average value (maximum between cue and reward) along with RPE at the time of cue across unscaled (*middle-right)* and scaled (*right*) trials for best fit TDRL model (Fig. 5c–e). **c.** For comparisons between models (Fig. 5), simulations were run for the same number of trials as experimental groups. If each condition is instead run for at least 400 trials, different learned trials are observed between 30 and 60 s ITI conditions in the best fit model. Such differences, however, do not hold across all ITI groups. *Left*, Trials to learn (when value > threshold) as a function of IRI for the best-fit TDRL simulation with ≥400 trials for each ITI (see *Methods*). Hexagons represent mean trials to learn across iterations (n = 20 each). Open circles represent experimental data (same data as Figs. 3g and 4f). Akaike information criterion (AIC) model weight comes from comparison with best fit SOP (**e**), and ANCCR (**g**) models. Please note that this AIC model does not penalize free parameters, that is, is a conservative estimate biased against ANCCR (see *Methods* for rationale and Supplementary Table 1 for AIC model weight when penalizing for parameters). *Middle*, Timecourses of value on each trial (maximum between cue and reward) or RPE at time of cue across iterations (n = 20 each) and plotted across scaled or unscaled trials. SEM occluded by thickness of mean lines. *Right*, Average RPE at time of cue during final conditioning trials. Bar height represents mean across iterations. Error bars represent SEMs, and circles represent individual iteration. **** p < 0.0001, Kruskal-Wallis. Post-hoc Mann–Whitney U planned comparisons: 60 s vs. 600 s, p < 0.0001; 60 s vs 3600 s, p < 0.0001; 600 s vs. 3600 s, p = 0.38. **d.** In principle, a similar rule derived in ANCCR could be applied *ad hoc* to any model of associative learning. Here, we demonstrate that applying such a rule to TDRL improves fit to experimental results. Using the best fit model parameters determined during initial TDRL parameter sweep (Fig. 5c–e), we replaced the learning rate, α, based on the equation α = 1 – *e* ^(−k· IRI)^ and performed another parameter sweep to determine the best fit *k*. *Left*, Trials to learn (when value > threshold) as a function of IRI for the best-fit TDRL simulation with scaled learning rate (see Methods). Hexagons represent mean trials to learn across iterations (n = 20 each). Open circles represent experimental data (same data as Figs. 3g and 4f). Akaike information criterion (AIC) model weight comes from comparison with best fit SOP (**e**), and ANCCR (**g**) models. Please note that this AIC model does not penalize free parameters, that is, is a conservative estimate biased against ANCCR (see *Methods* for rationale and Supplementary Table 1 for AIC model weight when penalizing for parameters). *Middle-left*, Timecourse of value on each trial (maximum between cue and reward) or RPE at time of cue (*middle*) for best fit *k* averaged across iterations (n = 20 each) and plotted across scaled trials. SEM occluded by thickness of mean lines. *Middle-right*, Mean RPE at time of cue during final conditioning trials. Bar height represents mean across iterations. Error bars represent SEMs, and circles represent individual iteration. *Right*, Total conditioning time prior to the emergence of behavior (threshold crossing). Model was simulated for 2400 trials per ITI group to accurately capture asymptotic RPE values. Symbols represent time for a single iteration, and bar height represents mean (n = 20 iterations per ITI condition). **** p < 0.0001, Kruskal-Wallis. Post-hoc Mann–Whitney U planned comparisons: 60 s vs. 600 s, 60 s vs 3600 s, 600 s vs. 3600 s, all p < 0.0001. **e.** Best fit SOP model: *Left* Trials to learn (when value > threshold) as a function of IRI for SOP simulation with best-fit parameter combination of experimental results (see *Methods*). Squares represent mean trials to learn across iterations (n = 20 each) for all conditions. Open circles represent experimental data (same as Figs. 3g and 4f). Akaike information criterion (AIC) model weight comes from comparison with best fit TDRL (**b**) and ANCCR (**g**) models. Please note that this AIC model does not penalize free parameters, that is, is a conservative estimate biased against ANCCR (see *Methods* for rationale and Supplementary Table 1 for AIC model weight when penalizing for parameters). *Middle-left*, Average value (maximum between cue and reward) *Middle-right*, pA2_reward_ across unscaled trials and *right*, pA2_reward_ across scaled trials (*right*) for best fit SOP model (Fig. 5f–h). **f.** In SOP, when a cue-reward association is learned, the cue attains the ability to activate the secondary, decayed representation of the reward (A2_reward_), which is thought to underlie conditioned responding to the cue after learning. pA2_reward_ at time of cue refers to the proportion of reward representation elements driven into the secondary activation state (A2) by cue presentation. In SOP, the decay of elements from A1 active state to the A2 secondary active state and from the A2 state to the Inactive state are controlled by the parameters *pd1* and *pd2*, respectively. Prior implementations of the model state that it is important to assume that pd1> pd2^43^ meaning that elements will more rapidly decay from A1 to A2 than from A2 to inactive. In our parameter sweep, we applied this constraint when determining the model best fit to the behavioral results (**e** and Fig. 5f–h). If this constraint is removed, the best fit parameter combination had pd1_reward_ = pd2_reward_. This panel shows trials to learn (when value > threshold) for this model as a function of IRI (*left*) as well as average timecourses of value and pA2 across scaled (*middle*) trial units. We conducted n = 20 iterations for each condition. SEM occluded by thickness of mean lines. *Left*, Open circles represent experimental data (same data as Figs. 3g and 4f). Akaike information criterion (AIC) model weight comes from comparison with best fit TDRL (**b**), and ANCCR (**g**) models. Please note that this AIC model does not penalize free parameters (see *Methods* for rationale and Supplementary Table 1 for AIC model weight when penalizing for parameters). *Right*, Total conditioning time prior to the emergence of behavior (threshold crossing). Symbols represent time for a single iteration, and bar height represents mean (n = 20 iterations per ITI condition. Note that there is a 3-fold difference in time to learn for 60 s vs 600 s ITI for this model, which is inconsistent with the experimental data. **g.** Best fit ANCCR model: *Left*, Trials to learn (when NC_cue⟷reward_ (net contingency) > threshold) as a function of IRI for ANCCR simulation with parameter combination that best fits experimental results (see *Methods*). Crosses represent mean trials to learn across iterations (n = 20 each). Open circles represent experimental data (same as Figs. 3g and 4f). Akaike information criterion (AIC) model weight comes from comparison with best fit TDRL (**b**) and SOP (**e**) models. *Right*, Mean ANCCR (the variable postulated to be encoded by dopamine in the ANCCR framework) during final conditioning trials from simulations in Fig. 5i–k. Bar height represents mean across iterations. Error bars represent SEMs, and circles represent individual iteration. **** p < 0.0001, Kruskal-Wallis. Post-hoc Mann–Whitney U planned comparisons: 60 s vs. 600 s, 60 s vs 3600 s, 600 s vs. 3600 s, all p < 0.0001.

The learning of all groups (30–600-s ITIs) progressed similarly across scaled trial numbers that maintain equivalent conditioning time (Fig. 3f). As total duration between trials in the previous experiments is equal to the IRI (or equivalently, the ICI due to 100% reward probability), we calculated learning as a function of IRI. We found a strong linear relation between log(trials to learn) and log(IRI) with a slope statistically indistinguishable from −1 (−1.06, *P* = 0.106; Fig. 3g), indicating inverse proportionality between trials to learn and IRI (or a proportional relationship between learning rate and IRI): for every increase in IRI by a factor of *n*, animals learn in *n* times fewer trials. Dopaminergic learning showed a similar inversely proportional relationship between trials to learn and IRI (slope: −1.04; Extended Data Fig. 6e), and the total conditioning time until the emergence of cue-evoked licking was statistically indistinguishable for the four groups (*P* = 0.94; Fig. 3h and Extended Data Fig. 7a). Thus, increasing IRI proportionally increases learning on each trial such that total conditioning time remains constant, consistent with hypothesis 3 but not hypothesis 1 or 2 (Fig. 1b,c).

**Fig. 4:**
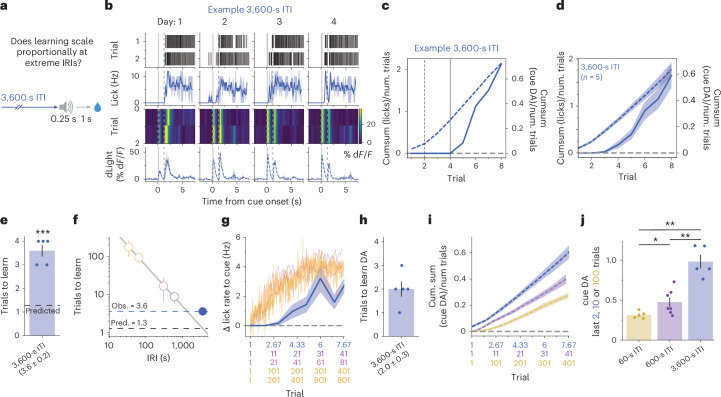
Learning rate increases, but not proportionally, with extreme trial spacing. **a**, A group of animals with a 3,600-s ITI were conditioned similarly to previous groups to test if the observed power law relationship between IRI and trials to learn holds at extreme IRIs. Animals were presented with two cue–reward pairings per session for a total session duration of 2 h. **b**, Example lick raster plots (upper row), lick PSTHs (2nd row), heat maps of dopamine responses on each trial (3rd row) and dopamine PSTHs (lower row) for one example 3,600-s ITI mouse showing every cue and reward presentation across 8 days of conditioning. Data are presented as in Fig. 2c. **c**, Cumsum of cue-evoked licks (solid line, left axis) or of normalized cue-evoked dopamine (dashed line, right axis) for the same example mouse as in **b**. Data are presented as in Fig. 2d. **d**, Average cumsums of cue-evoked licks and normalized cue-evoked dopamine in 3,600-s ITI mice (*n* = 5). **e**, Number of trials for 3,600-s ITI (*n* = 5) mice to learn cue–reward association. Bar height represents mean trial after which mice show evidence of learning. Dashed line represents predicted trials to learn based on relationship observed for 30–600-s ITI groups (best-fit line in Fig. 3g). Values under labels represent the mean ± s.e.m. ***P < 0.001, one-sample *t*-test versus predicted. **f**, 3,600-s ITI mice (blue circle, filled) take more trials to learn than predicted from the proportional scaling relationship in Fig. 3g. White filled circles and line are the same data as in Fig. 3g. Dashed lines represent predicted and mean observed trials to learn for 3,600-s ITI mice. **g**, Learning in 3,600-s ITI mice does not scale proportionally with other groups. Average cue-evoked licking for 30-s, 60-s, 300-s, 600-s (30–600-s ITIs, same data as in Fig. 3f) and 3,600-s (blue, *n* = 5) ITI mice across scaled trial numbers. Previously displayed data are shown without error to aid visualization. **h**, Mean dopamine learned trial for 3,600-s ITI mice (*n* = 5). Values under labels represent the mean ± s.e.m. **i**, Average cumsum of normalized cue-evoked dopamine responses in 60-s ITI (gold, same data as Fig. 2i), 600-s ITI (purple, same data as Fig. 2i) and 3,600-s ITI (blue, *n* = 5) mice. Cumsum curves divided by number of trials to account for differences between groups. Transparency added to previously displayed data to aid visualization. Shaded region represents the s.e.m. **j**, Asymptotic normalized cue-evoked dopamine after learning is highest in 3,600-s ITI mice. Bars represent means for trials 301–400 (60-s ITI), trials 31–40 (600-s ITI) or trials 15–16 (3,600-s ITI mice). *P < 0.05, **P < 0.01, Welch’s *t*-test.

This strong proportional relationship between learning rate and IRI implies that at extreme IRIs, animals could learn cue–reward associations in one or two trials. To test this, we conditioned a ‘3,600-s ITI’ group of mice with identical trial structure, but a 3,600-s ITI (two trials per day, ~2-h session time; Fig. 4a), which are predicted to learn within 1.3 trials (Fig. 3g). The 3,600-s ITI mice rapidly learned the cue–reward association within 3.6 trials, significantly more than predicted (*P* < 0.001; Fig. 4b–f and Extended Data Fig. 6f), but significantly fewer than 600-s ITI mice (*P* < 0.0001; Extended Data Fig. 6g). Thus, while learning rate increased with IRI, the proportional scaling broke down at this extreme interval (Fig. 4g). Dopaminergic learning showed a similar pattern: cue-evoked dopamine emerged after two trials (Fig. 4h), significantly fewer than in 600-s ITI mice (*P* < 0.01), but not in line with the proportional scaling seen with shorter IRIs (Extended Data Fig. 6i,j). Interestingly, cue-evoked dopamine (relative to maximum reward response), but not licking, had a significantly greater asymptote (0.98 ± 0.08) than in 60-s ITI (0.31 ± 0.02, *P* < 0.01) or 600-s ITI (0.47 ± 0.06, *P* < 0.01) mice, and was also significantly greater in 600-s ITI versus 60-s ITI mice (0.47 ± 0.06 versus 0.31 ± 0.02, *P* < 0.05; Fig. 4i,j and Extended Data Fig. 6h,k–p). Thus, extreme IRIs increase learning rate and asymptotic dopamine, but not proportionally.

## ANCCR, a model of retrospective causal learning, captures the proportional scaling of learning rate by IRI

Given the experimentally observed proportional scaling of learning rate with the time between trials, we assessed whether any learning model can naturally explain these results. We recently proposed a dopamine-based learning framework, adjusted net contingency (NC) of causal relations (ANCCR), that learns retrospectively whether a cue consistently precedes reward^35^. The learned retrospective association, *p*(*c* | *r*) (‘how often does cue precede reward?’) is then converted to a prospective association, *p*(*r* | *c*) (‘how often does reward follow cue?’) using Bayes’ rule by multiplying *p*(*c* | *r*) by a normalization factor equal to the ratio of the overall probability of the reward and of the cue: $p({r|c})=p({c|r})\frac{p(r)}{p(c)}$. Here, *p*(*c* | *r*) is updated at rewards (definition of conditional probability), while the normalization factor $\frac{p(r)}{p(c)}$ is updated continuously. For the Bayes’ rule to be appropriate in a dynamic world, both terms must be calculated over the same temporal window of past experience. For example, if *p*(*c* | *r*) is calculated over 1 h, it is inappropriate to normalize it by $\frac{p(r)}{p(c)}$ calculated over the last 1 min, because the world may have changed over the last hour. This directly implies that the cue–reward learning rate per reward should scale proportionally with IRI.

**Fig. 5:**
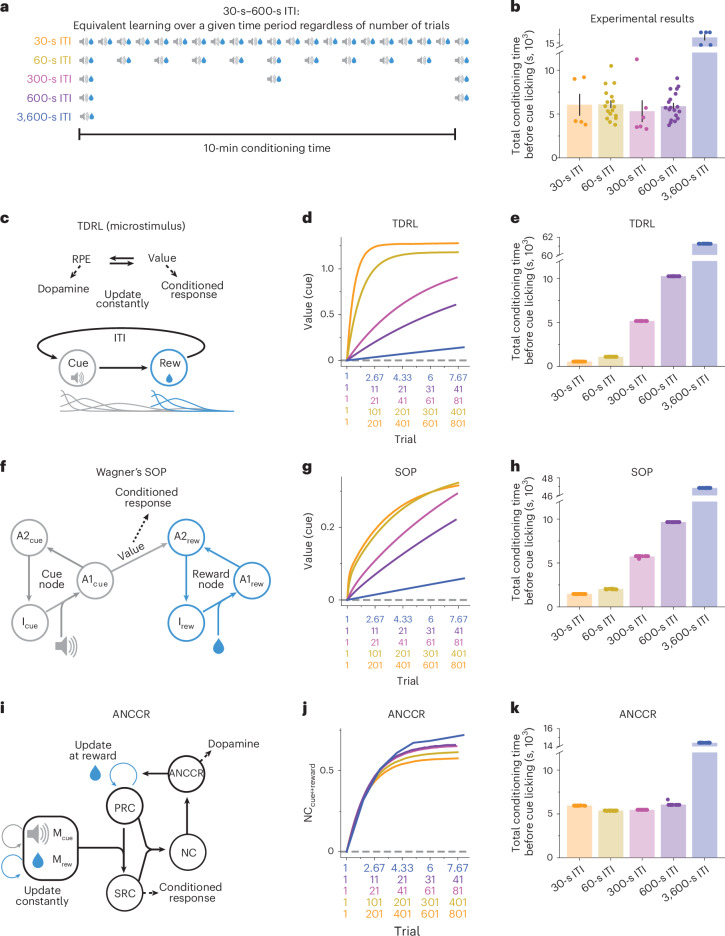
Proportional scaling of learning rate by IRI is only captured by a retrospective learning model. **a**, Diagram of number of trials experienced by each ITI group over a 10-min interval. Based on the experimentally observed proportional scaling of learning rate by trial spacing (Fig. 3g), learning is equivalent during this interval regardless of the number of trials experienced for 30–600-s ITI, but not 3,600-s ITI mice. Note that multiple 3,600-s trials are not shown to maintain scale. **b**, Total conditioning time before the emergence of anticipatory licking from experiments in Figs. 1, 3 and 4. Circles represent individual mice, and bar height represents group mean. The 30–600-s ITI groups display the same data as in Fig. 3h. **c**, Schematic diagram of microstimulus implementation of TDRL. At each moment, a value estimate of future rewards is updated by an RPE, the deviation from the current value estimate. RPE is thought to be encoded in the brain by dopamine signaling. Value is used to drive behavioral responding. The microstimulus implementation of TDRL was chosen because external stimuli (that is, cues and rewards) evoke microstimuli, which spread representations of the stimuli in time. Because the model contains ITI states that themselves can acquire value, the model contains a potential mechanism to explain how the spacing between trials could affect learning rate. Parameter combinations were swept to determine if any set could capture the quantitative scaling observed in the experimental results where learning rate varied proportionally with the IRI. **d**, Time course of value on each trial (maximum between cue and reward) for the best-fit TDRL model (**c**) averaged across iterations (*n* = 20 each) and plotted across scaled trials for all conditions. The s.e.m. is occluded by line thickness. **e**, Total conditioning time before the emergence of behavior (threshold crossing). Circles represent time for a single iteration, and bar height represents the mean (*n* = 20 iterations per ITI). **f**, Schematic diagram of Wagner’s SOP model of associative learning. Presentations of cues or rewards evoke presumed mental representations or processing nodes consisting of many informational elements. These stimulus representations are dynamic: presentation of a stimulus moves a portion of elements from (only) the inactive (I) state into the primary active state (A1). Elements then decay into the secondary active state (A2) and then decay again to the inactive state while the stimulus is absent. Elements transition between states according to individually specified probabilities. Cue–reward associations (value) are strengthened and learned when cue elements in A1_cue_ and reward elements in A1_reward_ overlap in time. Following learning, cues evoke conditioned responding by directly activating reward elements to their A2 state. The SOP theory has been hypothesized to explain ITI impact on learning because more time between trials allows more elements to decay to the inactive state, allowing for a greater number of elements to transition to the A1 active state upon next cue and reward presentation. Parameter combinations were swept to determine if any set of parameters could capture the quantitative scaling observed in the experimental results. **g**, Time course of value on each trial (maximum between cue and reward) for the best-fit SOP model (**f**) averaged across iterations (*n* = 20 each) and plotted across scaled trials. The s.e.m. values are occluded by line thickness. **h**, Total conditioning time before the emergence of behavior (threshold crossing). Circles represent time for a single iteration, and bar height represents the mean (*n* = 20 iterations per ITI). **i**, Schematic diagram of ANCCR. See the main text for an explanation of learning rate scaling in ANCCR. **j**, Time course of NC_cue⟷reward_ on each trial for the best-fit ANCCR model (**i**) averaged across iterations (*n* = 20 each) and plotted across scaled trials. The s.e.m. values are occluded by line thickness. **k**, Total conditioning time before the emergence of behavior (threshold crossing). Symbols represent time for a single iteration, and bar height represents the mean (*n* = 20 iterations per ITI).

The temporal window of past experience used for learning is related to learning rate. Intuitively, when overall learning rate increases, recent experience is more emphasized than older experience. The overall learning rate of a quantity over some time period is the amount of learning per update multiplied by the number of updates. Therefore, to equate the amount of past experience used to update *p*(*c* | *r*) and $\frac{p(r)}{p(c)}$, the learning rate per *p*(*c* | *r*) update times its update frequency (equal to reward frequency) should be equal to the learning rate per $\frac{p(r)}{p(c)}$ update times its update frequency. Because the latter product is approximately constant, the learning rate per *p*(*c* | *r*) update should be a constant multiplied by the time between *p*(*c* | *r*) updates, which is IRI (1/frequency of reward). Thus, because ANCCR updates cue–reward associations retrospectively only at rewards, it predicts that learning rate should be proportional to the time between rewards, or IRI (that is, ten times the learning per reward for a ten times less frequent reward; see the Methods for detailed derivation). This rule is consistent with the experimentally observed proportional relationship between learning rate and trial spacing (Fig. 3g), and explains the constant total conditioning time across a 20-fold variation in the rate of trials (Fig. 5a,b).

While the proportional scaling observed experimentally is consistent with ANCCR, other models may better fit the results. To quantitatively test this, we compared three candidate models of conditioning (Fig. 5 and Extended Data Fig. 7): (1) the microstimulus implementation of TDRL^41^, which allows ITI states to acquire value, thereby containing a mechanism for trial spacing effects (longer ITIs reduce ITI value and increase cue RPE, similarly to ‘context extinction’^22^); (2) Wagner’s standard operating procedure or sometimes opponent process (SOP)^42,43^, which is not a dopaminergic learning model, but has been proposed to explain trial spacing effects^29^; and (3) ANCCR^35^, which retrospectively infers the cause of rewards and predicts a strong proportional scaling of learning rate by IRI.

We then quantitatively compared how well each model captured behavioral learning using Akaike information criterion (AIC)-derived relative weights (Methods). Over the range of the parameters tested, the best-fit microstimulus model did not capture the observed proportional scaling in either value (presumed to drive behavior) or RPE (presumed to be encoded by dopamine) and requires more conditioning time to learn with increased spacing (AIC: 445.5, compared to best-fit SOP and ANCCR: 4.4 × 10^−22^; Fig. 5d,e and Extended Data Fig. 7a,b). A TDRL model with learning rate artificially scaled by IRI using the relationship derived from ANCCR provides an approximate fit to behavior (AIC: 352.7, compared to best-fit SOP and ANCCR: 0.0643), but still requires more conditioning time to learn with increased spacing and did not reproduce the asymptotic dopamine cue response (Extended Data Fig. 7a,d). The best-fit SOP model exhibited a qualitative trial spacing effect^22,25,29^ but did not produce proportional scaling and requires more conditioning time to learn with increased spacing (AIC: 373.5, compared to best-fit TDRL and ANCCR: 1.9 × 10^−6^; Fig. 5g,h and Extended Data Fig. 7a,e). In contrast, ANCCR captured both the proportional scaling of learning rate and the asymptotic dopamine cue responses, and maintained constant total conditioning time to learn across ITIs from 30 s to 600 s (AIC: 347.2, compared to best-fit TDRL and SOP: 1; Fig. 5j,k and Extended Data Fig. 7a,g). Therefore, proportional scaling with trial spacing is categorically absent in common TDRL frameworks and SOP but emerges due to ANCCR’s retrospective updates.

## Learning rate scaling is not explained by number of experiences per day, context extinction, overall rate of auditory stimuli or overall rate of rewards

**Fig. 6:**
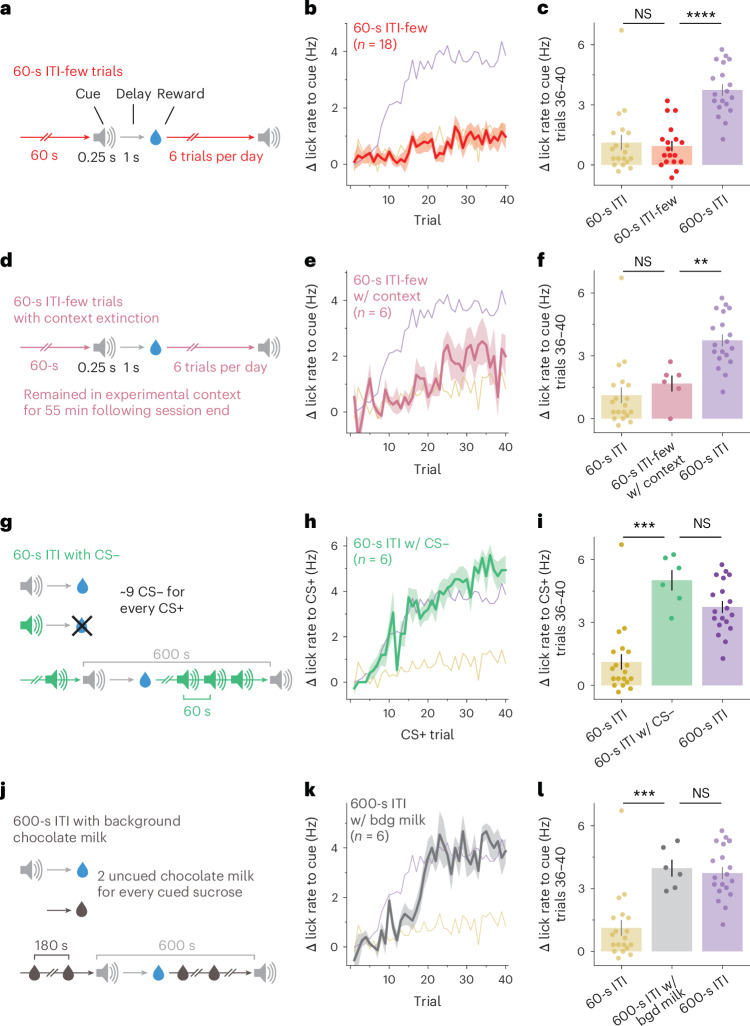
Learning rate scaling is not explained by number of experiences per day, context extinction, overall rate of auditory cues or overall rate of rewards. **a**–**c**, Number of trials per day does not explain differences in learning rates between 60-s and 600-s ITI mice. **a**, Schematic of conditioning for ‘60-s ITI-few’ group conditioned with the same ITI as 60-s ITI mice (mean, 60 s) but the same number of trials/rewards per day as 600-s ITI mice (six). **b**, Time course of average cue-evoked licking for 60-s ITI-few (red, *n* = 18), 60-s ITI (same data as Fig. 1e) and 600-s ITI (same data as Fig. 1e) mice over 40 trials. The 60-s and 600-s ITI time courses are shown transparent and without error for visualization purposes. **c**, Mean cue-evoked licking (trials 36–40). The 60-s ITI-few mice show significantly less cue-evoked licking than 600-s ITI mice and behave like 60-s ITI mice. ****P < 0.0001, Welch’s *t*-tests. **d**–**f**, Context extinction does not explain difference in learning rates between 60-s and 600-s ITI mice. **d**, Schematic of conditioning for ‘60 s ITI-few with context extinction’ group. Mice were conditioned similarly to 60-s ITI-few mice but remained in the experimental context for ~55 min following the final conditioning trial, matching 600-s ITI group’s time in context and number of cue–reward experiences, while the rate of rewards during trials matched the 60-s ITI group. **e**, Time course of average cue-evoked licking for 60-s ITI-few with context extinction (light pink, *n* = 6), 60-s ITI (same data as Fig. 1e) and 600-s ITI (same data as Fig. 1e) mice over 40 trials. **f**, Mean cue-evoked licking (trials 36–40). The 60-s ITI-few with context extinction mice show significantly less cue-evoked licking than 600-s ITI mice and are not significantly different from 60-s ITI mice. **P < 0.01, Welch’s *t*-tests. **g**–**i**, Overall rate of auditory cues does not explain differences in learning rates between 60-s and 600-s ITI mice. **g**, Schematic of conditioning for ‘60-s ITI with CS−’ group. Mice were conditioned similarly to 600-s ITI mice but during the (~600-s) interval between CS + →reward trials, distractor CS− cues (3 kHz pure tone) were presented on average every 60 s to match the rate of auditory cues experienced by 60-s ITI mice. All mice could hear and respond to CS− as evidenced by some generalized licking (Extended Data Fig. 9c,d). **h**, Time course of average cue-evoked licking for 60-s ITI with CS− (green, *n* = 6), 60-s ITI (same data as Fig. 1e) and 600-s ITI (same data as Fig. 1e) mice over 40 trials of conditioning. **i**, Mean cue-evoked licking (trials 36–40). The 60-s ITI with CS− mice show significantly more cue-evoked licking than 60-s ITI and are not significantly different from 600-s ITI mice. ***P < 0.01, Welch’s *t*-tests. **j**–**l**, Learning rate is not scaled by overall rate of rewards. **j**, Schematic of conditioning for ‘600 s ITI with background chocolate milk’ group. Mice were conditioned similarly to 600-s ITI mice but during the (~600-s) interval between cue→sucrose trials, mice received two uncued deliveries of chocolate milk ~180 s apart to test whether cue–sucrose learning rate is affected by the general or identity-specific rate of rewards. Mice readily consumed chocolate milk rewards upon delivery (Extended Data Fig. 9h). **k**, Time course of average cue-evoked licking for 600-s ITI with background chocolate milk (gray, *n* = 6), 60-s ITI (same data as Fig. 1e) and 600-s ITI (same data as Fig. 1e) mice over 40 trials. **l**, Mean cue-evoked licking (trials 36–40). The 600-s ITI with background chocolate milk mice show significantly more cue-evoked licking than 60-s ITI and are not significantly different from 600-s ITI mice. ***P < 0.01, Welch’s *t*-tests.

**Extended Data Fig. 8:**
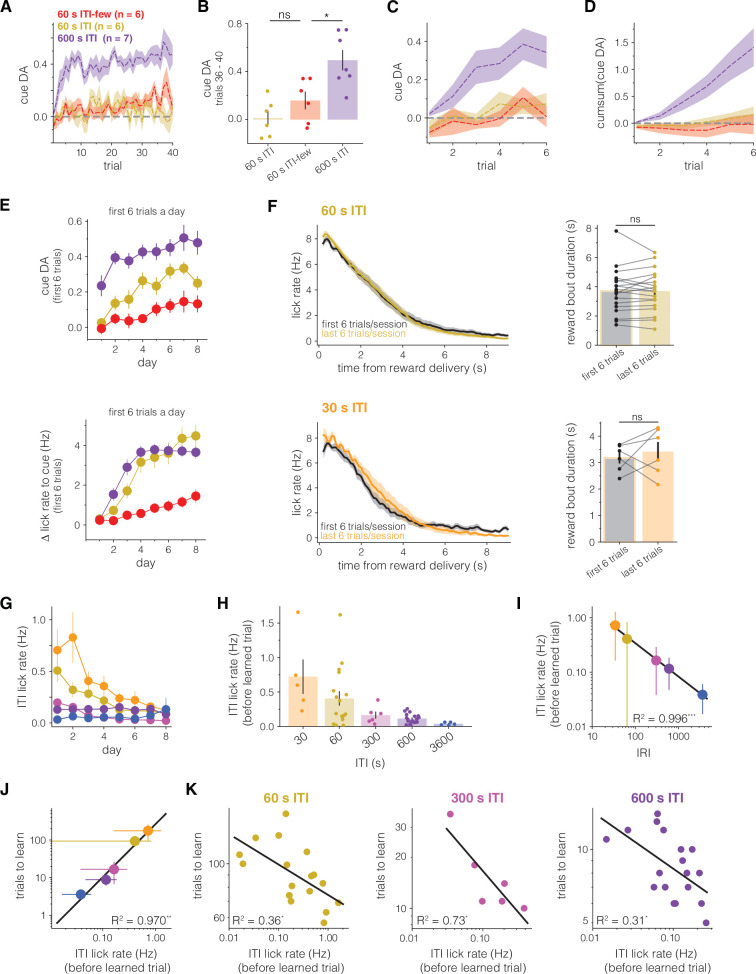
Satiety does not account for differences in learning between groups (a-f), and lick rate during ITI scales with reward rate, and therefore, trials to learn (g-k). **a-b**. Cue-evoked dopamine in 60 s ITI-few mice (red, n = 6) evolves similarly to that of 60 s ITI mice and is significantly lower than that of 600 s ITI dopamine at the end of conditioning. **a**. Timecourse of normalized cue-evoked dopamine across first 40 trials of conditioning. Lines represent animal means and shaded region represents SEM. **b**. Mean cue-evoked dopamine from trials 36-40. *p < 0.05, ns: not significant. **c-d**. Average (**c**) or cumulative sum (**d**) of normalized cue-evoked dopamine responses in 60 s ITI-few (red, n = 6), 60 s ITI (gold, n = 5), and 600 s ITI (purple, n = 7) over the first 6 trials on the first day of conditioning, which are the first exposures to both cues and rewards and during which satiety is not expected to differ between groups. Lines represent means across animals and shaded region represents SEM. Note the similarities between 60 s ITI-few and 60 s ITI groups, and the greater dopamine responses in 600 s ITI despite equivalent cumulative number of rewards experienced between groups in the first session before any overnight period. **e**. Average cue-evoked dopamine (*top*) and cue-evoked lick rates (*bottom*) for the first six trials on each day in 60 s ITI-few (lick: n = 18, cue DA: n = 6), 60 s ITI (lick: n = 19, cue DA: n = 6), and 600 s ITI (lick: n = 19, cue DA: n = 7) mice. Dopamine responses between 60 s ITI and 60 s ITI-few mice diverge across conditioning, suggesting that satiety following the first 6 trials throughout a session does not drive the differences in dopamine signaling or lick behavior seen between groups. **f**. Average reward bout duration for first 6 trials and last 6 trials a session is not different in 60 s ITI (n = 19, *top*) or 30 s ITI (n = 6, *bottom*) mice, suggesting a lack of within session satiety and decreased motivation. *Left¸* Lick PSTHs aligned to reward delivery. *Right*, Average duration of reward licking bout for first 6 trials and last 6 trials per session averaged across eight conditioning days. Error bar represents SEM. Circles represent individual mice, and lines connect data from a single mouse. ns: not significant, paired t-test. **g**. Average lick rate during the ITI (defined as licks following cessation of reward consumption bout until next cue onset) over days. Note the clear separation between groups in early days that greatly reduces over conditioning, consistent with greater context-reward associations in shorter ITI groups that extinguish over time. **h**. Average ITI lick rates preceding each individual animal’s learned trial. **i**. Average ITI lick rates preceding learning, as a function of inter-reward interval (IRI) plotted on log-log axis. Circles represent mean trials to learn per group and error bars represent standard deviation. Solid black line is best fit regression line (R^2^ = 0.996, *** p < 0.001). This suggests a strong positive relationship between reward rate and ITI licking, consistent with differential context-reward associations between groups due to an effect of context extinction (that is lower rate of reward in the context during long ITI). **j**. Average learned trial as a function of ITI lick rates preceding learning plotted on log-log axis. Circles represent mean trials to learn per group and error bars represent standard deviation. Solid black line is best fit regression line (R^2^ = 0.970, ** p < 0.001), suggesting an inverse relationship between ITI lick rate and learning rate. **k**. Contrary to what may be predicted from the between-group relationship (**i**), within each ITI group there is a positive relationship between ITI lick rate and learning rate, suggesting reduced context-reward associations are not the primary driver of cue-reward learning. *Left*, 60 s ITI (R^2^ = 0.36, ** p < 0.05), *middle*, 300 s ITI (R^2^ = 0.73, ** p < 0.05), *right*, 600 s ITI (R^2^ = 0.31, ** p < 0.05).

Although learning rate scaling by IRI appears inconsistent with trial-based learning theories, several confounds preclude a strong conclusion. First, longer ITIs involve fewer rewards per day, potentially increasing cue–reward salience^44,45^ via novelty or reduced satiety, and enhance overnight consolidation (mice learned in 2–3 days on average). To control for these factors, we conditioned a ‘60-s ITI-few’ group with the same ITI as 60-s ITI mice (mean, 60 s) and the same number of trials per day as 600-s ITI mice (six), while recording dopamine from a subset (Fig. 6a). Learning in these mice progressed similarly to 60-s ITI mice, with both cue-evoked licking and dopamine significantly lower than 600-s ITI mice (lick: 60-s ITI-few: 0.9 ± 0.3 Hz versus 600-s ITI: 3.7 ± 0.3 Hz, *P* < 0.0001; versus 60-s ITI: 1.1 ± 0.4 Hz, *P* > 0.99; dopamine: 60-s ITI-few: 0.16 ± 0.07 versus 600-s ITI: 0.49 ± 0.08, *P* < 0.05; versus 60-s ITI: 0.01 ± 0.06, *P* = 0.33; Fig. 6b,c and Extended Data Fig. 8a,b). Further, over the first six trials when satiety or day effects do not differ between groups, cue-evoked dopamine increased only in 600-s ITI mice (Extended Data Fig. 8c–e). Moreover, shorter ITI groups show consistent reward consumption across a session (Extended Data Fig. 8f), indicating that satiety does not explain learning rate differences.

A second confound is that longer ITIs potentially accelerate learning via context extinction^22^. To test this, we conditioned a ‘60-s ITI-few with context extinction’ group identical to 60-s ITI-few but kept mice in the context for ~55 min after trials, matching the 600-s ITI group in context time and cue–reward count, while matching the 60-s ITI group in reward rate (Fig. 6d). Learning in these mice again progressed similarly to 60-s ITI mice (*P* > 0.99), with significantly less cue-evoked licking (1.7 ± 0.4 Hz) than 600-s ITI mice (*P* < 0.01; Fig. 6e,f). Moreover, licking during the ITI positively correlates with learning rate within groups, suggesting that context extinction does not explain learning rate differences (Extended Data Fig. 8g–k).

**Extended Data Fig. 9:**
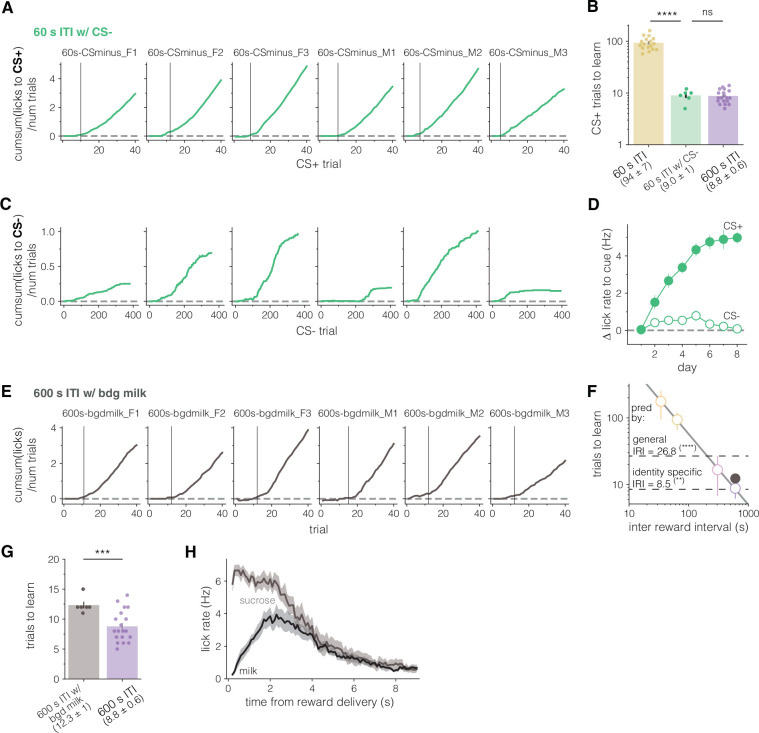
Trials to learn in “60 s ITI with CS-” and “600 s ITI with background milk” mice. **a.** Cumulative sum of CS+ evoked licks for all “60 s ITI with CS- mice” across 40 trials. **b.** 60 s ITI with CS- mice learn in significantly fewer CS+ trials than 60 s ITI, similar to 600 s ITI mice (60 s and 600 s ITI: same data as Fig. 1g). **** p < 0.0001 not significant, Welch’s t-test. **c.** Cumulative sum of CS- evoked licks for all 60 s ITI with CS- mice across all CS- presentations before the 40^th^ CS+ trial. Note that despite the lower average licks to CS- compared to CS+ licking (**a**), every mouse showed at least some CS- evoked licking, evidence that all mice could hear and respond to the tone. **d.** Timecourse of average CS+ (filled) or CS- (open) evoked lick rate as a function of days of conditioning. Circles represent mean change in lick rate per day, and error bars represent SEMs. **e.** Cumulative sum of cue-evoked licks for all 600 s ITI with background milk mice across 40 trials. **f.** Predicted and actual trials to learn from experiment shown in Fig. 6j–l. Dashed lines represent predicted trials to learn based the relationship determined in Fig. 3g using either the general IRI including both chocolate milk and sucrose (upper line) or using the interval between sucrose deliveries alone (lower line). The actual observation (brown filled circle) is much closer to a scaling by an identity-specific IRI of the sucrose than a general IRI of either sucrose or chocolate milk. The fact that the actual trials to learn do not exactly match the identity-specific IRI suggests slight generalization between sucrose and chocolate milk rewards due to shared sensory features (for example, both are sweet, liquid rewards). Open circles and gray line same data as Fig. 3g. ****p < 0.0001, **p < 0.01. One-sample t-test. **g.** Mean trials to learn in 600 s ITI with background milk vs. 600 s ITI (same data as Fig. 1g) mice. **h.** Average lick PSTH aligned to reward delivery for sucrose (gray) or chocolate milk (black) for all 600 s ITI with background milk over eight days of conditioning. Shaded error represents SEM across mice. *** p < 0.001, Welch’s t-test.

A third confound is the overall rate of auditory stimuli, which could alter cue salience or enable replay/reactivation-based ‘virtual trials’^46^. To control for this, we conditioned a ‘60-s ITI with CS−’ group like 600-s ITI mice but presented distractor CS− tones (3 kHz, ~every 60 s) during the ~600-s interval between CS+ reward trials, matching the auditory cue rate of 60-s ITI mice (Fig. 6g). Learning in these mice resembled 600-s ITI mice (*P* = 0.22), with significantly more CS+ licking (5.0 ± 0.5 Hz) than 60-s ITI mice (*P* < 0.001; Fig. 6h,i). Furthermore, they learned the CS+ reward relationship in nine trials, no different than 600-s ITI mice (*P* > 0.99), yet significantly fewer than 60-s ITI mice (*P* < 0.0001; Extended Data Fig. 9a–d).

Finally, a fourth confound is the overall reward rate. ANCCR predicts learning rate should scale with identity-specific IRI, and not overall reward rate. To test this, we conditioned a ‘600-s ITI with background chocolate milk’ group like 600-s ITI mice but delivered two uncued chocolate milk rewards during the ~600-s interval between cue–sucrose trials (Fig. 6j). Learning in these mice progressed similarly to 600-s ITI mice (*P* > 0.99), with significantly more cue-evoked licking (4.0 ± 0.4 Hz) than 60-s ITI mice (*P* < 0.001; Fig. 6k,l). These mice learned in 12 ± 1 trials, significantly fewer than the general IRI prediction (26.8 trials from relationship in Fig. 3g; *P* < 0.0001), but also significantly more than both the identity-specific IRI prediction, which assumes perfect reward discriminability (8.5 trials; *P* < 0.01), and 600-s ITI mice (*P* < 0.001; Extended Data Fig. 9e–h). These results are consistent with learning rate scaling by an identity-specific IRI with some generalization between sweet liquids.

## Partial reinforcement scales learning rate by increasing the IRI

**Fig. 7:**
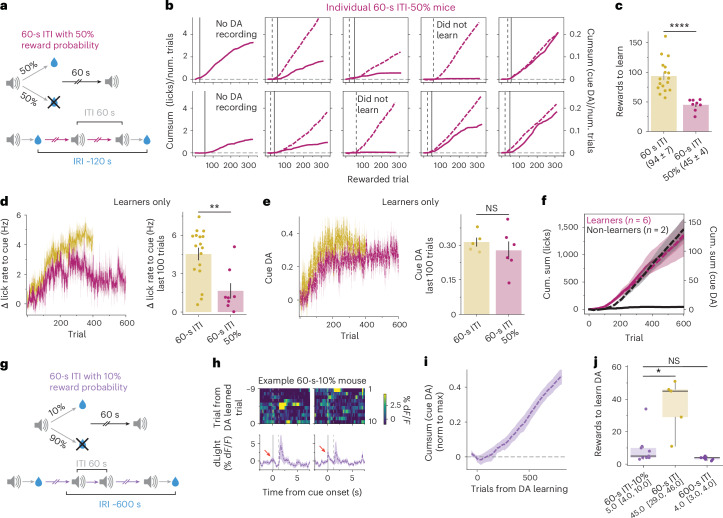
Partial reinforcement scales learning rate by increasing the IRI. **a**, Schematic of 60-s ITI-50% partial reinforcement conditioning. Mice were conditioned identically to 60-s ITI mice (Fig. 1) except rewards were delivered with 50% reward probability for 50 trials with ~25 rewards a day. Reducing the reward probability by 50% leads to a doubling of the IRI to ~120 s on average across a session while maintaining the ITI and ICI. Mice were conditioned for 12 days. **b**, Cumsum of cue-evoked licks (solid line, left axis) or normalized cue-evoked dopamine (dashed line, right axis) as a function of rewarded trials for all 60-s ITI-50% mice. Data are presented as in Fig. 2d. Dopamine was not recorded from two initial mice and two other mice did not show evidence of behavioral learning (Methods). **c**, The 60-s ITI-50% mice (magenta, *n* = 8) learn the cue–reward association in about half the number of rewards as 60-s ITI mice (same data as Fig. 1g). Bar height represents mean number of rewards after which mice show evidence of learning. Values under labels represent the mean ± s.e.m. Mice that did not show evidence of learning (**b**) were excluded from analysis. ****P < 0.0001, Welch’s *t*-test. **d**,**e**, Among mice that learned the cue–reward association, 60-s ITI-50% mice show lower asymptotic lick rates than 60-s ITI mice, consistent with prior literature on partial reinforcement; however, asymptotic dopamine responses do not show statistically significant differences between groups suggesting a dissociation between behavior and dopamine. Left, Time course of cue-evoked licking (**d**) or normalized cue-evoked dopamine (**e**) across all conditioning trials for 60-s ITI-50% (magenta, *n* = 8 behavior, *n* = 6 dopamine) and 60-s ITI (gold, *n* = 17 behavior, *n* = 5 dopamine) mice. Right, Mean cue-evoked licking (**d**) and normalized cue-evoked dopamine (**e**) during the last 100 trials of conditioning for both groups. **P < 0.01, Welch’s *t*-test. **f**, Further suggesting a dissociation between behavior and dopamine, non-learner mice (black, *n* = 2) show intact dopaminergic learning similarly to behavioral learner mice (magenta, *n* = 6). Cumsum of cue-evoked licks (left axis, solid line) or normalized cue-evoked dopamine (right axis, dashed line) over all 600 trials. **g**, Schematic of 60-s ITI-10% partial reinforcement conditioning. Mice were conditioned identically to 60-s ITI mice (Fig. 1) except rewards were delivered with 10% reward probability for 50 trials with ~5 rewards a day. Reducing the reward probability by 10% led to a tenfold increase in IRI (~600 s on average across a session) while maintaining the ITI and ICI. Mice were conditioned for 32 days. **h**, Example 60-s ITI-10% mouse showing dopamine response to cue and reward for the 10 trials preceding and following the ‘dopamine learned trial’. Red arrows indicate the increased dopamine cue response after the identified learned trial. Note that the PSTHs average both rewarded and omission trials together. **i**, Average across mice of the cumsum of cue dopamine following aligned to each animal’s dopamine learned trial. Note the consistently positive response following the dopamine learned trial. **j**, 60-s ITI-10% dopamine cue responses emerge in a comparable number of rewards to 600-s ITI mice. Box lines represent median rewards until dopamine learned trial. Box edges represent the interquartile range (IQR). Whiskers extend to data points lying within 1.5 times the IQR. Circles represent individual mice. Values under labels represent the median and IQR. The outlier mouse showing learned trial after 34 rewards shows robust evidence of dopamine learning within ~5–7 rewards, but has later trials with low cue response, thereby resulting in a later identified learned trial (Extended Data Fig. 10h,i). *P < 0.05, Mann–Whitney *U*-test.

**Extended Data Fig. 10:**
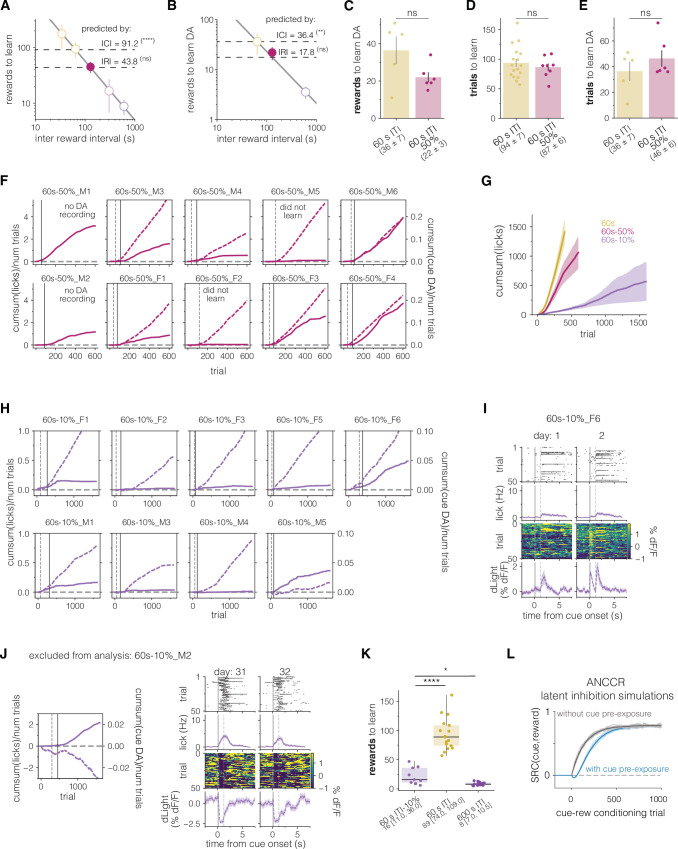
Results from 60 s ITI-50% and 60 s ITI-10% mice. **a.** Predicted and actual rewards to learn in 60 s ITI-50% mice. The previous experiments did not disambiguate the inter-cue interval (ICI) from the IRI because they are equivalent when rewards follow cues with 100% probability. If the learning rate is scaled by the CS + ICI rather than the IRI, 60 s ITI-50% mice would be expected to learn after 91.2 rewards based on the relationship determined in Fig. 3g (upper dashed line), similar to 60 s ITI mice, which have an identical inter-cue interval. However, if IRI is the driver of learning rate scaling 60 s ITI-50% would be expected to learn in 43.8 rewards (lower dashed line). The observed number of rewards to learn (magenta filed circle), 45, is consistent with learning rate scaling by IRI. Open circles and gray line same data as Fig. 3g. ****p < 0.0001, ns: not significant. One-sample t-test. **b.** Predicted and actual rewards until the emergence of cue-evoked dopamine in 60 s ITI-50% mice. As in **a**, ICI scaling of learning rate predicts dopaminergic learning in 36.4 rewards, while IRI scaling of learning rate predicts 17.8 rewards. The observed (magenta filed circle) dopaminergic learning in 22 rewards is consistent with learning rate scaling by IRI. Open circles and gray line are the same data as Extended Data Fig. 6e. **p < 0.01, ns: not significant. One-sample t-test. **c.** Number of rewards after which cue-evoked dopamine emerges in 60 s ITI-50% and 60 s ITI (same data as Fig. 2e) mice. Mice that did not show evidence of learning or did not have dopamine recordings (Fig. 7b) were excluded from analysis. ns: not significant. Welch’s t-test. **d.** 60 s ITI-50% mice learn the cue-reward association in about the same number of cue presentations/trials as 60 s ITI mice (same data as Fig. 1g). Mice that did not show evidence of learning (Fig. 7b) were excluded from analysis. Note that for 60 s ITI mice, cues/trials to learn and rewards to learn represent the same values (Fig. 7c). Ns: not significant. Welch’s t-test. **e.** Number of trials after which cue-evoked dopamine emerges in 60 s ITI-50% and 60 s ITI (same data as Fig. 2e) mice. Note that for 60 s ITI mice, cues/trials to learn and rewards to learn dopamine represent the same values (**c**). ns: not significant. Welch’s t-test. **f.** Cumulative sum of cue-evoked licks (solid, left axis) or of normalized cue-evoked dopamine (dashed, right axis) as a function of all trials for all 60 s ITI-50% mice. Both lick and cue-evoked dopamine values were divided by total trial number to display average responses across conditioning. Solid vertical lines represent learned behavior trial and dashed vertical lines represent dopamine (cue) learned trial. Dopamine was not recorded from two initial mice and two other mice did not show evidence of behavioral learning (see *Methods*). **g.** Group averages of cumulative sum of licks across trials for 60 s ITI (gold), 60 s ITI-50%(maroon), and 60 s ITI-10%(light purple). Note that despite lower lick rates and asymptotic performance for 60 s ITI-10% mice, they show consistent increase across trials. **h.** Cumulative sum of cue-evoked licks (solid, left axis) or of normalized cue-evoked dopamine (dashed, right axis) as a function of all trials for all 60 s ITI-10% mice. Both lick and cue-evoked dopamine values were divided by total trial number to display average responses across conditioning. Solid vertical lines represent learned behavior trial and dashed vertical lines represent dopamine (cue) learned trial. Due to the overall lower rate and frequency of anticipatory licking in these, learners and non-learners were not distinguished and behavioral learned trial was calculated on all mice. **i.** One 60 s ITI-10% outlier mouse’s calculated dopamine learned reward (Fig. 7j) was calculated at 34 rewards due to a dip in the cue DA response following initial emergence of cue evoked dopamine (**h**). Visual inspection of the first two days of conditioning shows clear cue-evoked dopamine following 5-7 rewards at the end of day 1/ beginning of day 2 of conditioning. **j.** One mouse was excluded from analysis of dopamine and behavior learned rewards due to a consistently negative dopamine response to the cue, contrasting with all other animals’ cue-responses recorded throughout the study. *Left*, cumulative sum curve of cue-evoked licking and behavior across all of conditioning for this animal demonstrating the negative Cumulative sum dopamine curve. *Right*, Lick raster and PSTH along with dopamine heatmap and PSTH for the final two days of conditioning (days 31 and 32). **k**. 60 s ITI-10% mice learned trial compared to 60 s ITI and 600 s ITI mice. Box lines represent median rewards until learned trial. Box edges represent interquartile range (IQR). Whiskers extend to data points lying withing 1.5 * IQR. Circles represent individual mice. Values under labels represent median and IQR. *p < 0.05, ****p < 0.0001, Mann-Whitney U test. **l**. Growth curves for the prospective association in simulations of the best-fit ANCCR model from Fig. 5 as a function of trials with (blue line) and without (grey line) prior exposure to cues, indicating the presence of latent inhibition when “omission” trials are presented in a block prior to conditioning. For the latent inhibition condition, 200 cue-only presentations were delivered prior to cue-reward conditioning. ITI in all cases was set to 30 s.

Although ANCCR predicts that learning rate scales with IRI, the previous experiments did not disambiguate IRI from ICI (CS+) because they are equal when reward probability is 100%. A counterintuitive prediction of the hypothesis that learning rate is scaled by IRI, and not ICI, is that reducing reward probability, and thus increasing IRI without affecting ICI, would decrease the number of cue–reward pairings needed to learn. This contrasts with the prediction that learning rate is controlled by the ratio between ITI and trial duration, which implies a constant number of rewards to learn regardless of partial reinforcement^27^. Thus, we conditioned a ‘60-s ITI-50%’ group identically to 60-s ITI but with 50% reward probability, doubling the IRI to ~120 s on average while maintaining the ITI and ICI (Fig. 7a). If the learning rate is scaled by ICI, it should occur in 91.2 rewarded trials (Fig. 3g), similarly to 60-s ITI. However, if learning rate is scaled by IRI, it should occur in 43.8 rewarded trials. Remarkably, 60-s ITI-50% mice took 45 ± 4 rewarded trials to learn, significantly fewer than 60-s ITI mice (*P* < 0.0001; Fig. 7b,c) or the ICI prediction (*P* < 0.0001), and not different from the IRI prediction (*P* > 0.99; Extended Data Fig. 10a). Dopaminergic learning showed the same scaling (22 ± 3 rewarded trials: *P* < 0.01 versus ICI predicted 36.4; *P* > 0.99 versus IRI predicted 17.8; Extended Data Fig. 10b,c). As rewarded trials make up about half the experienced trials, 60-s ITI-50% mice learn the behavior in an equivalent number of trials/cue presentations (87 ± 6) as 60-s ITI mice (*P* = 0.44; Extended Data Fig. 10d,f) despite only half being rewarded. This relationship was also observed for dopaminergic learning (46 ± 6 trials, *P* = 0.34 versus 60 s ITI; Extended Data Fig. 10e,f).

While 2/10 of 60-s ITI-50% mice did not learn the cue–reward association, of the mice that did learn, cue-evoked licking at the end of conditioning was less than half that of 60-s ITI mice (60 s: 4.5 ± 0.5 Hz, 60 s-50%: 1.65 ± 0.6 Hz, *P* < 0.01; Fig. 7d), consistent with prior literature on reduced reward-seeking during partial reinforcement^47^. However, cue-evoked dopamine did not differ between 60-s ITI-50% and 60-s ITI mice (60 s: 0.31 ± 0.02, 60 s-50%: 0.28 ± 0.04 Hz, *P* = 0.43; Fig. 7e), demonstrating a dissociation between dopamine and behavior. Supporting this dissociation, non-learner 60-s ITI-50% mice still develop cue-evoked dopamine responses, consistent with the emergence of behavior (but not dopamine) requiring a threshold crossing of the evidence for the cue–reward association (Fig. 7f). Thus, the dopamine cue response provides a more sensitive measure of learning than anticipatory licking.

We next tested whether further extending the IRI by reducing reward probability to 10% produces a proportional increase in learning rate. Thus, we conditioned a ‘60-s ITI-10%’ group (Fig. 7g). Because dopaminergic learning is a more sensitive measure of learning than behavior (Fig. 7f), we assessed whether cue-evoked dopamine emerges with ten times fewer rewards due to a 60-s ITI with 10% reward probability, comparable to the 600-s ITI group with 100% reward probability. Although the conditioned behavior was lower with 10% reinforcement, it consistently increased across trials (Extended Data Fig. 10g,h). The onset of conditioned behavior measured as the median number of rewards to learn (16 rewards) was closer to the 600-s ITI group (eight rewards) than the 60-s ITI group (89 rewards; Extended Data Fig. 10k). Despite the low probability of reinforcement, we found a robust increase in dopamine cue responses over trials (Fig. 7h,i). Cue-evoked dopamine emerged after a median of five rewards, comparable to the 600-s ITI group (median of four rewards; *P* = 0.10; Fig. 7j and Extended Data Fig. 10h,i). Thus, even under extreme partial reinforcement, the underlying neural learning rate is scaled by the IRI. While asymptotic performance reduces with partial reinforcement, the underlying dopaminergic response emerges with proportionally fewer cue–reward experiences.

**Fig. 8:**
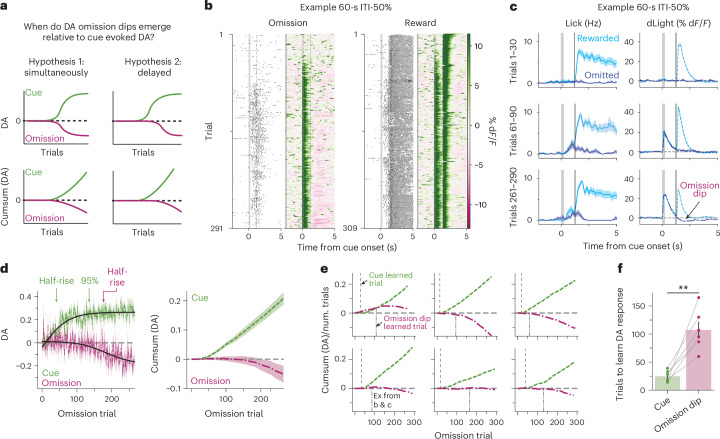
Emergence of dopamine dip to reward omission is inconsistent with canonical RPE signaling. **a**, Diagrams of two hypothesized relationships between the emergence of cue-evoked dopamine and reward omission-driven dopamine dips over the course of learning. Hypothesis 1 is based on the canonical TDRL framework of dopamine signaling in which a positive RPE at cue implies a positive cue value, and a positive cue value implies a negative RPE upon omission of the reward (regardless of whether omission dip is smaller than the reward burst). Thus, positive cue response and negative omission response coevolves over trials despite differences in kinetics or magnitude. If the learning of cue value is slowed due to a gradual ‘backpropagation’ of a TDRPE signal, negative omission response should instead appear even sooner than the positive cue response because the state immediately before outcome will acquire a positive value sooner than the cue onset. Hypothesis 2 is based on the ANCCR framework, which assumes that a reward omission becomes a meaningful event only after the cue–reward association exceeds some internal threshold. Thus, ANCCR predicts that the suppression of dopamine response to reward omission should occur well after the emergence of the dopamine cue response. **b**, Lick raster and heat map of dopamine aligned to cue onset for one example 60-s ITI-50% mouse during omission (left) and rewarded (right) trials across conditioning. **c**, Lick (left) and dopamine (right) PSTHs aligned to cue onset for the same example mouse as in **b**. Light blue represents data from trials where rewards were delivered, while dark blue represents trials where rewards were omitted. Data are binned into early conditioning (trials 1–30, top), middle conditioning (trials 61–90, middle) and late conditioning (trials 261–290, bottom). In the middle row, note the prominent cue-evoked dopamine and licking and the absence of a dopamine dip following reward omission. **d**, On average, reward omission-driven dopamine dips emerge later than cue-evoked dopamine in 60-s ITI-50% mice (*n* = 6). Average (left) and cumsum (right) of normalized dopamine responses to cue presentation (green, dashed line) or reward omission (magenta, dash-dot line) across reward omission trials. Black line represents sigmoid fits to experimentally observed cue and omission responses (Supplementary Table 1). Green arrows represent the trials at which cue response reaches 50% (41 trials) or 95% (135 trials) of its peak. Magenta arrow represents half-rise trial (176 trials) for omission response. **e**, Omission dips emerge later than cue-evoked dopamine in individual mice. Cumsum of normalized dopamine responses to cue presentation (green, dashed line) or reward omission (magenta, dash-dot line) across omission trials for all 60-s ITI-50% mice with dopamine recordings that learned the cue–reward association. Dashed lines on the upper half of the plots represent the omission trial after which cue-evoked dopamine emerges (dopamine learned trial), and dash-dot lines on the bottom of the plots represent the omission trial after which dips in dopamine following reward omission emerge. **f**, On average, cue-evoked dopamine emerges 82 omission trials before dips in dopamine following reward omission. Bar height represents the mean number of omissions before cue-evoked dopamine (green) or reward omission-driven dips in dopamine (magenta) begin. Error bars represent the s.e.m. Circles represent individual mice, and lines connect data from a single mouse. **P < 0.01, paired *t*-test.

We next used our dataset to further disambiguate between ANCCR and canonical temporal difference reward prediction error (TDRPE) models. In TDRL, cue and omission responses coevolve, or omission dips may even precede cue responses if TDRPE backpropagates within a trial^48^. In ANCCR, omissions gain meaning only after cue–reward learning surpasses a threshold^35^, predicting that the suppression of dopamine to omission should occur well after the emergence of cue responses (Fig. 8a). We found a clear distinction in the evolution of cue-evoked dopamine and the delayed emergence of dopamine dips in response to reward omissions (Fig. 8b–d). Cue-evoked dopamine emerges after 25 ± 4 omission trials, while dopamine dips appeared only after 107 ± 15 omission trials (*P* < 0.01; Fig. 8e,f). Cue responses reach 95% of their asymptote well before omission dips reach their half-maximum, indicating that omission dips emerge after cue response has stabilized, inconsistent with canonical TDRPE models. Although TDRPE modifications in which reward timing is learned after cue–reward association (similarly to ANCCR) may account for these results, they are inconsistent with gradual TDRPE backpropagation^48^. While these results constrain future TDRPE models, they are naturally consistent with ANCCR.

## Discussion

Here, we demonstrate a mathematical rule underlying cue–reward learning rate: the rate of behavioral and dopaminergic learning scales proportionally with the duration between rewards. Consequently, the total conditioning time needed for learning is independent of 20-fold variation in the number of experiences. This rule extends to aversive cue–shock learning where ISI controls learning rate. Consistent with IRI scaling of cue–reward learning rate, partial reinforcement effectively extends the IRI and counterintuitively increases learning rate per reward experience. Thus, over a fixed duration, increasing the number of cue–reward experiences does not increase overall learning, providing a qualitative constraint on associative learning models. We show that the experimentally observed mathematical rule underlying learning rate control emerges naturally from Bayes’ rule in outcome-triggered retrospective learning. These results require a reevaluation of the frameworks used to describe associative learning and a reassessment of the implicit assumption that ‘trials’ are the fundamental unit of experience^24^.

While standard TDRL implementations ignoring the ITI cannot, by definition, explain IRI scaling of learning rate, we demonstrate that a TDRL extension that ascribes lower state value during longer ITIs also cannot account for the observed proportional scaling of dopaminergic and behavioral learning. One could add IRI-scaled learning rates to TDRL to fit the data (Extended Data Fig. 7d), but this is ad hoc because temporal difference updates occur at every time step and provide no principled basis for proportionally scaling updates with IRIs, especially in an identity-specific manner. In contrast, ANCCR updates cue–reward associations at reward times, making IRI a natural determinant of learning rate. In the absence of current alternatives that derive scaling of learning rate by IRI from first principles, ANCCR provides a parsimonious explanation of these findings based on Bayes’ rule.

One potential concern for the generality of ANCCR given the partial reinforcement results (Fig. 7) is whether retrospective learning driven purely by rewards is consistent with latent inhibition (the slower acquisition of cue–reward associations following cue exposure without rewards^49^). However, during cue-only preexposure, the model learns a high baseline cue rate, thereafter requiring more cue–reward pairings to infer contingency (Extended Data Fig. 10l). Formally, elevated marginal cue probability with unchanged cue–reward probability reduces retrospective contingency, slowing learning.

A ‘trial-based’ explanation of our findings could assume that replays/reactivations of cue–reward experiences during the extended ITIs or sleep^50,51^ provide ‘virtual trials’ in lieu of real experiences^46^. If so, a single real experience should in principle be sufficient to produce a never-ending stream of replays, unless competing experiences, especially those occurring more frequently, dominate^46^. The ‘60-s ITI with CS−’ experiment (Fig. 6g), in which more frequent CS– omissions failed to disrupt CS+ reward learning, challenges this hypothesis. If replay instead preferentially favors rewarded experiences^52^, it becomes similar to ANCCR, and may be a mechanistic implementation of retrospective learning.

Here, we primarily focused on dopamine-based alternative theories of learning. However, some psychological/algorithmic/statistical theories are applicable here. The SOP theory^42,43^ captures a qualitative increase in learning rate by ITI, but does not explain how overall learning over a fixed duration is independent of the number of experiences. The behavioral learning data with 100% reward probability are generally consistent with timescale invariance—the observation that the number of trials to learn does not change when the entire timeline of conditioning is multiplied by a constant scale factor. However, the observation that partial reinforcement produces proportionally more learning per reward is inconsistent with timescale invariance and the information-based theories aiming to explain it^53–56^, which predict no change because both the total experienced trial duration and the total experienced ITI are equally lengthened between rewards^53^. Nevertheless, an information-based account triggered by rewards (that is, retrospective learning) has been suggested for this result.

ANCCR postulates that mesolimbic dopamine gates retrospective learning, thereby modulating learning rate. This aligns with recent models challenging the dopamine-TDRPE framework, such as the Kutlu–Calipari–Schmajuk model, where dopamine conveys perceived salience, influencing associability and therefore learning rate^57^, and the Dudman lab’s policy learning model, where dopamine directly modulates learning rate^37^. Therefore, all three models (and related work in flies^58^) propose that instead of signaling TDRPE, dopamine modulates learning rate (although not its sole determinant^11,59^). However, aside from ANCCR, these models do not explain IRI scaling of cue–reward learning rate. While there is accumulating evidence that mesolimbic dopamine does not function strictly as a TDRPE^34–37,40,57,60–62^, the current results call into question the broader trial-based reinforcement learning framework used to understand dopamine and learning.

Our results challenge the ‘practice makes perfect’ principle often associated with trial and error or skill learning. We speculate that repetition dependence in skill learning likely arises from factors such as sensory or motor discrimination demands (for example, distinguishing 440 Hz from 444 Hz). Collectively, we provide a new parsimonious framework for understanding dopamine-mediated cue–reward learning^35^ that explains the proportionality between cue–reward learning rate and IRI in both dopaminergic and behavioral measures. Because retrospective learning updates occur at every reward, ANCCR does not rely on experimenter-defined trials and thus avoids potentially problematic assumptions about trial structure^63^. This makes ANCCR well suited for explaining learning in naturalistic settings without arbitrary trial periods, and for providing a mechanistic basis for IRI scaling by grounding learning rate in the real-time dynamics of dopamine signaling.

## Methods

### Animals

All experiments and procedures were performed in accordance with guidelines from the National Institutes of Health (NIH) Guide for the Care and Use of Laboratory Animals and approved by the UCSF Institutional Animal Care and Use Committee. In total, 101 adult (>11 weeks at time of experiments; median: 13 weeks) wild-type male and female C57BL/6J mice (JAX; RRID: IMSR_JAX:000664) were used across 13 experimental groups: 30-s ITI (*n* = 6; 3 F/3 M), 60-s ITI (*n* = 19; 13 behavior-only: 7 F/6 M and 6 dopamine + behavior: 4 F/2 M), 300-s ITI (*n* = 6; 3 F/3 M), 600-s ITI (*n* = 19; 12 behavior-only: 5 F/7 M and 7 dopamine + behavior: 5 F/2 M), 3,600-s ITI (*n* = 5; all dopamine + behavior: 3 F/3 M), 60-s ITI-few trials (*n* = 18; 12 behavior-only: 6 F/6 M and 6 dopamine + behavior: 3 F/3 M), 60-s ITI-few trials with context extinction (*n* = 6; 3 F/3 M), 60-s ITI with CS− (*n* = 6; 3 F/3 M), 600-s ITI with background milk (*n* = 6; 3 F/3 M), 60-s ITI-50% (*n* = 10; 2 behavior-only: 0 F/2 M and 8 dopamine + behavior: 4 F/4 M) 60-s ITI-10% (*n* = 10; 5 F/5 M all dopamine + behavior), 45-s ISI (*n* = 8; 4 F/4 M all dopamine + behavior) and 135-s ISI (*n* = 7; 3 F/4 M all dopamine + behavior). One 60-s ITI mouse implanted with an optic fiber was excluded from all dopamine analyses due to a missed fiber placement (Extended Data Fig. 3). One 60-s ITI mouse that was implanted with an optic fiber failed to learn the cue–reward association (Extended Data Fig. 2c) and was excluded from dopamine analyses comparing behavioral and dopaminergic learning (Fig. 2). Two 60-s ITI-50% mice implanted with dopamine fibers failed to learn the cue–reward association (Fig. 7b and Extended Data Fig. 10f) and were excluded from comparisons against 60-s ITI mice and omission dip calculations (Figs. 7c and 8d–f and Extended Data Fig. 10). While small sample sizes preclude a rigorous analysis of sex differences across all conditions tested, no significant difference in ‘trials to learn’ was found between females and males in either the 60-s or 600-s ITI group, the two conditions best powered to detect differences. Thus, sexes were pooled for all analyses.

All cue–reward conditioned mice were head-fixed during conditioning and underwent surgery before behavior experiments either to implant a custom head ring for head fixation (behavior-only) or to inject viral vector and implant an optic fiber and head ring (dopamine + behavior; see ‘Surgery’). All cue–shock conditioned mice were freely moving during experiments but underwent surgery to inject viral vector and implant optic fibers. Mice were >7.5 weeks old at time of surgery (median, 9 weeks). Following surgery, mice were given at least a week to recover before beginning water deprivation. Mice implanted with optic fibers did not begin experiments until >3.5 weeks following surgery to allow time for virus expression. During water deprivation (cue–reward conditioned mice only), mice were given ad libitum access to food but were water deprived to ~85–90% of pre-deprivation body weight and maintained in that weight range throughout experiments through daily adjustments to water allotment. Mice were weighed and monitored daily for the duration of deprivation. After surgery, mice were randomly assigned to experimental groups and those with only a head ring implant were group housed in cages containing mice from multiple experimental groups, while fiber-implanted mice were single housed. Mice were housed on a reverse 12-h light–dark cycle with lights off from 8:00 to 20:00, and all behavior was run during the dark cycle. The mouse holding room was maintained at ~23–24 °C with 40–50% humidity.

### Surgery

Surgery was performed under aseptic conditions. Mice were anesthetized with isoflurane (5% induction, ∼1–2% throughout surgery) and placed in a stereotaxic device (Kopf Instruments) and kept warm with a heating pad. Before incision, mice were administered carprofen (5 mg per kg body weight, subcutaneously (s.c.)) for pain relief, saline (0.3 ml, s.c.) to prevent dehydration and local lidocaine (1 mg per kg body weight, s.c.) to the scalp for local anesthesia. All mice were implanted with a custom-designed head ring (5-mm inner diameter, 11-mm outer diameter, 3-mm height) on the skull for head fixation. The ring was secured to the skull with dental acrylic supported by screws. Following surgery, mice were given buprenorphine (0.1 mg per kg body weight, s.c.) for pain relief.

To measure dopamine release in a subset of mice, 500 nl of an adeno-associated viral (AAV) vector encoding the dopamine sensor dLight1.3b (AAVDJ-CAG-dLight1.3b, 3.9 × 10^13^ genome copies (GCs) per ml diluted in sterile saline to a final titer of 3.9 × 10^12^ GCs per ml or AAV5-CAG-dLight1.3b, 2.4 × 10^13^ GCs per ml diluted to 4–5 × 10^12^ GCs per ml) was injected unilaterally into the nucleus accumbens core (from bregma: AP, 1.3; ML, ±1.4; DV, −4.55), in either the right or the left hemisphere, counterbalanced across groups. Viral vectors were injected through a small glass pipette with a Nanoject III (Drummond Scientific) at a rate of 1 nl s^−1^. The injection pipette was kept in place for 5–10 min to allow diffusion, then slowly retracted to prevent backflow up the injection tract. Following injection, an optic fiber (NA 0.66, 400 μm, Doric Lenses) was implanted 200–350 μm above the virus injection site. Following fiber implant, the head ring was secured to skull as above. After experiments, fiber-implanted mice were transcardially perfused, and brains were fixed in 4% paraformaldehyde. Brains were sectioned at 50 µm and imaged on a Keyence microscope to verify fiber placement. Histology images presented (Extended Data Fig. 3) represent composites imaged with a ×10 objective. Stitched images of full brain slices were then cropped to focus on fiber placements in the nucleus accumbens (Extended Data Fig. 3b) or dorsal striatum (Extended Data Fig. 3c).

### Cue–reward conditioning

For experiments in Figs. 1–4, all animals were conditioned with an identical trial structure (see below), differing only in ITI as well as number of trial presentations to keep the total conditioning time roughly equal between groups (~1h). For Figs. 1 and 2, 60-s ITI mice were run for 50 trials a day with a variable ITI with a mean of 60 s (uniformly distributed from 48 s to 72 s). The 600-s ITI mice were run for 6 trials a day with a variable ITI with a mean of 600 s (uniformly distributed from 480 s to 720 s). For Fig. 3, 30-s ITI mice were run for 100 trials a day with a variable ITI with a mean of 30 s (uniformly distributed from 24 s to 36 s). The 300-s ITI mice were run for 11 trials a day with a variable ITI with a mean of 300 s (uniformly distributed from 240 s to 360 s). For Fig. 4, 3,600-s ITI mice were run for 2 trials a day with fixed ITI of 3,600 s and, unlike other groups, the session time lasted 2 h.

For experiments in Fig. 6, additional groups of mice were conditioned with parameters matching those of 60-s and/or 600-s ITI groups to control for the influence of factors that varied along with ITI (IRI) manipulations. The 60-s ITI-few mice were run for 6 trials a day (same as 600-s ITI mice) with a mean ITI of 60 s (uniformly distributed from 48 s to 72 s; same as 60-s ITI) to control for the difference in total trial experiences per day between 60-s ITI and 600-s ITI mice. Unlike other groups, sessions lasted ~6.5 min. The 60-s ITI-few mice with context extinction were conditioned similarly to 60-s ITI-few mice but remained in the experimental context for ~55 min following the end of conditioning trials, matching 600-s ITI group’s time in context and number of cue–reward experiences, while the rate of rewards during trials matched the 60-s ITI group. The 60-s ITI with CS− mice were conditioned similarly to 600-s ITI mice, for 6 (CS+) trials a day with a variable (CS+) ITI with a mean of 600 s (uniformly distributed from 480 s to 720 s); however, during the interval between CS+ trials, distractor CS− cues (0.25-s, 3-kHz constant tone, delivered through a piezo speaker: https://www.adafruit.com/product/1739) were presented. CS− cues were not followed by reward delivery and were delivered on a variable interval (exponentially distributed) with a mean of 60 s to approximate the rate of cue delivery in 60-s ITI mice. All mice could hear and respond to the CS− cue as evidenced by some generalized licking to the CS− during conditioning (Extended Data Fig. 9c,d). The 600-s ITI with background chocolate milk mice were conditioned similarly to 600-s ITI mice but, during the interval between cue–sucrose trials (mean of 600 s, uniformly distributed from 480 s to 720 s), mice received two uncued deliveries of chocolate milk (Nesquik Low Fat Chocolate Milk) separated from the previous sucrose or chocolate milk delivery by a variable interval with a mean of 180 s (uniformly distributed from 144 s to 216 s) to test whether cue–sucrose learning rate is affected by the general or identity-specific rate of rewards. Volume of chocolate milk was calibrated to match that of sucrose reward delivery (2–3 µl). Mice readily consumed chocolate milk rewards upon delivery (Extended Data Fig. 9h).

For experiments in Fig. 7, 60-s ITI-50% mice were conditioned identically to 60-s ITI mice (variable ITI with a mean of 60 s, uniformly distributed from 48 s to 72 s), except rewards were delivered with 50% reward probability for 50 trials with ~25 rewards a day to disambiguate the (CS+) ICI from the IRI. Reducing the reward probability by 50% led to a doubling of the IRI to ~120 s on average across a session while maintaining the ITI and ICI. The 60-s ITI-50% mice were conditioned for 12 days. The 60-s ITI-10% mice were conditioned similarly, but with a 10% probability of reward, increasing the IRI tenfold relative to 60-s ITI mice, similarly to 600-s ITI mice. The 60 s ITI-10% mice were conditioned for 32 days.

Trials (CS+) consisted of a 0.25-s 12-kHz constant tone through a piezo speaker (https://www.adafruit.com/product/1740) followed by a 1-s delay (trace period) after which sucrose-sweetened water (2–3 µl; 15% wt/vol) was delivered through a gravity-fed solenoid to a lick spout in front of the mouse, controlled by custom MATLAB and Arduino scripts^64^. After each outcome, there was a fixed 3-s period to allow reward consumption. Lick spout was positioned close to the animals such that animals could sense, but were not touched by, delivery of reward. Licks were detected through a complete-the-circuit design and recorded in MATLAB. Occasionally, certain mice would have long unbroken contacts with the spout (as measured by lick off–lick on time, due to grabbing the spout with their hands or not breaking contact with their tongues), occluding our ability to measure multiple licks during the period of contact. This was not corrected for as it generally happened following reward delivery or during the ITI and thus did not affect measurements of cue-evoked licks, our main variable of interest.

Mice were not habituated to the head-fixation apparatus or sucrose delivery before conditioning to minimize uncued reward exposure, which, we hypothesize, could affect retrospective contingency calculations during initial cue–reward learning. For the majority of mice, the first trial was their first experience of liquid sucrose reward. An initial subset of behavior-only 600-s ITI mice (*n* = 6) ran with a fixed ITI of 600 s and was given a single uncued reward delivery before conditioning on day 1. No gross difference in learning compared to subsequent groups was detected, and data were pooled. For all other groups on day 1, mice were placed in the head-fixation apparatus and conditioning commenced. Because a minority of animals from each condition did not initially consume sucrose at time of reward delivery, for all analyses, ‘trial 1’ was defined as the first trial in which a mouse licked to consume sucrose within 5 s of reward delivery. This design choice did not affect our main conclusions as analyzing ‘trials to learn’ in 30-s–3,600-s ITI mice without dropping any initial trials from analysis shifted the mean learned trial by <1 trial. To appropriately count omission trials, this was not done for partial reinforcement experiments, and trial 1 started with the first trial the animal was presented with regardless of licking behavior.

Mice were run for at least 8 days of conditioning, and trial analyses included the first 800 (30-s ITI), 400 (60-s ITI), 80 (300-s ITI) 40 (600-s ITI, 60-s ITI-few, 60-s ITI-few with context extinction, 60-s ITI with CS−, 600-s ITI with background chocolate milk) or 7–8 (3,600-s ITI) trials. For 60-s ITI-50% and 60-s ITI-10% mice, trial analyses included the first 600 and 1,600 trials, respectively. For omission trial-specific analyses (60-s ITI-50%), all omission trials occurring within the first 600 trials were analyzed (~300).

### Cue–shock conditioning

For cue–shock conditioning (Extended Data Fig. 5), two groups of freely moving mice were conditioned with an identical trial structure (15-s cue, 2-s trace period, 1-s shock), but differing in the ISI and number of trials a day. Compared to cue–reward conditioned mice, a longer cue period was necessary to measure freezing during the cue. The 45-s ISI mice received 13 trials a day with a mean ISI of 45.33 s (ITI of 27.33 s), while 135-s ISI mice received 5 trials a day with a mean ISI of 136 s (ITI of 118 s). The range of ISI values for each group was variable in the range of the mean ± 20%. The beginning of each conditioning session started with 300 s before the onset of trial 1 for both groups. This time is included in the analysis of total time to learn. The groups were matched so that each group spent the same amount of conditioning time in the chamber during each session leading to the different trial numbers. Three sessions of conditioning were conducted for each group on three consecutive days. Before the first conditioning day, mice were handled for 2 days and on the third day were acclimated to the photometry cables and habituated in the recording chambers for 20 min.

Conditioning took place in Med-Associates chambers with electric shock grid floors controlled by MED-PC. The cue was a 5-kHz tone (80 dB) and the shock was a scrambled electric shock (0.3 mA) delivered through the floor grid. Both the intensity of tone and shock were measured each day before recording. Each conditioning session was done in the same context (purple light, shock grid bottom, vanilla scent). Top-down videos of the chambers were recorded in each session for movement and freezing analysis.

### Fiber photometry

For cue–reward conditioning, fluorescent dLight signals were collected using either a Doric Fiber Photometry Console or pyPhotometry^65^ system. For both systems, light from 470-nm (~40 µW) and 405-nm (~25 µW) LEDs integrated into a fluorescence filter minicube (Doric Lenses) was passed through a low-autofluorescence patchcord (400 µm, 0.57 NA, Doric Lenses) to the mouse. Emission light was collected through the same patchcord, bandpass filtered through the minicube and measured with a single integrated detector. For the Doric system, excitation LED output was sinusoidally modulated by a Doric Fiber Photometry Console running Neuroscience Studio (v5.4 or v6.4) at 530 Hz (470 nm) and 209 Hz (405 nm). The console demodulated the incoming detector signal producing separate emission signals for 470 nm of excitation (dopamine) and 405 nm of excitation (dopamine-insensitive isosbestic control). Signals were sampled at 12 kHz and subsequently downsampled to 120 Hz following low-pass filtering at 12 Hz. For the pyPhotometry system, 405-nm and 470-nm excitation LEDs were modulated in time, rather than frequency, with separate brief 0.75-ms pulses used to separate isosbestic and signal channels. Data were sampled at 130 Hz and low-pass filtered at 12 Hz to match data from the Doric system. Due to a software error during file saving in the Doric system, the final trial was not recorded on two occasions (one 60-s ITI, one 600-s ITI) and was excluded from analysis. This error occurred either well before (60-s ITI) or well after (600-s ITI) the emergence of learning and thus had minimal effect on the resulting analysis. For one 3,600-s ITI animal, a pyPhotometry system crash during the ITI between trials 1 and 2 on day 5 resulted in ~15 min of photometry data loss during the ITI but did not affect analysis focused on cue and reward delivery epoch. A transistor–transistor logic pulse signaling behavior session start and stop was recorded by the photometry software to sync and align photometry and behavior data across hardware.

For cue–shock conditioning, fluorescent dLight signals were collected using an RWD R821 Tricolor MultiChannel Fiber Photometry System running OFRS software (version 2.0.0.33169). Excitation 470-nm (dopamine, ~40 µW) and 410-nm (isosbestic, ~15 µW) channels were separated through modulation in time. Each signal was turned on and off sequentially at an overall sampling rate of 60 Hz for an effective sampling rate of 30 Hz for the combined signals. Emission signals were filtered through dichroic filters in the system and detected with a CMOS camera. Transistor–transistor logic pulses sent from MED-PC to the photometry system during cue and shock synchronized the photometry signals with behavior.

### Analysis

#### Cue–reward behavior

The behavioral measure of learning here was licking in response to the cue before reward delivery. As mice learn the cue–reward association, cue presentation elicits anticipatory licking behavior toward the reward spout. To measure the cue-evoked change in licking behavior over baseline, the number of licks in the 1.25-s baseline period before cue onset was subtracted from the number of licks in the 1.25-s period from cue onset to reward delivery to calculate the change in licking behavior to the cue (cue-evoked licks). When this number was converted to a rate, it was reported as ‘Δ lick rate to cue’. To binarize cue-evoked licking behavior, we also measured the proportion of mice in each group that made more than one cue-evoked lick on each trial across conditioning (Extended Data Figs. 1 and 2). To visualize average trial licking behavior for each session in example animal plots (Figs. 1d, 2c, 3b, 4b, 7h and 8c and Extended Data Figs. 1a, 4a and 10i,j) or reward delivery aligned group averages (Extended Data Figs. 8f and 9h), lick PSTHs were generated by binning licks into 0.1-s bins, converting to a rate, and averaging across trials. The resulting average lick rate trace was smoothed with a Gaussian filter (sigma = 0.75) to aid visualization.

To calculate the trial at which animals show evidence of learning, we first took the cumsum of the cue-evoked licks^23,35,66–68^. Then drawing a diagonal from beginning to the end of the cumsum curve, we calculated the first trial that occurred within 75% of the maximum distance from the curve to the diagonal, which corresponded to the trial after which cue-evoked licking behavior emerged (Extended Data Fig. 2a–c). This trial was designated the ‘learned trial’. Occasionally after learning, cue licks taper off. If at the calculated learned trial the diagonal line was underneath the cumsum curve, which means that the mouse’s lick behavior was decreasing at that point rather than increasing, we iteratively reran the algorithm by drawing the diagonal from the beginning to the point on the cumsum curve corresponding to the previously calculated trial until at the new calculated trial the diagonal was above the cumsum curve (corresponding to the trial in which lick behavior begins to increase). Note that we use the first trial within 75% of maximum distance rather than the overall maximum distance (which would be the largest inflection point in the curve) to account for variability in post-learning behavior that occasionally caused the maximum distance from the diagonal to be at a point after a mouse has consistently licked to the cue for many trials; however, this choice did not affect the main conclusion of the analysis in Fig. 1 that 600-s ITI mice learn in ten times fewer trials than 60-s ITI mice (Extended Data Fig. 2d). Mice that did not show a > 0.5-Hz average increase in lick rate to cue for at least two sessions were classified as non-learners (Fig. 7b and Extended Data Figs. 2c, 6c and 10f) and were not considered in comparisons of learned trials (Figs. 1g, 3e and 7c and Extended Data Figs. 9b and 10c–e). Due to the lower average lick rates in 60-s ITI-10% animals, compared to all other groups tested, we did not segregate this group into learners and non-learners. Learned trial analyses were run on all animals in this group because despite the lower lick rates, all animals had positive slopes in the cumsum curves of licking behavior demonstrating consistent cue-evoked licking across trials (Extended Data Fig. 10g,h). To determine the ‘rewards to learn’ for animals conditioned with partial reinforcement (Fig. 7 and Extended Data Fig. 10), the trials to learn were calculated, and then the number of rewards delivered before the learned trial were counted for each animal. To measure the steepness of individual animal learning curves, we calculated the abruptness of change at the learned trial as the distance from the cumsum curve to the diagonal described above. This distance was calculated in normalized units where the top of the diagonal was set to equal 1 (Extended Data Fig. 2h). Cumsum data are occasionally displayed divided by the number of trials (yielding a *y* axis that corresponds to average response across all prior conditioning trials) to better compare across groups that experienced different numbers of trials.

To quantify the relationship between learning rate and IRI (Fig. 3g), the mean trials to learn for 30-s, 60-s, 300-s and 600-s ITI groups were plotted against the IRI (mean ITI + 4.25 s (1.25 s trial period + 3 s consummatory period)) on a log–log plot, and a linear least-squares regression was used to determine the best-fit line yielding the equation: log(trials_to_learn) = (−1.0593)log(IRI) + 3.8753. The slope and intercept determined here were used to calculate the predicted trials or rewards to learn for 3,600-s ITI (3,604.25-s IRI; Fig. 4e,f), 600-s ITI with background milk with a general IRI (604.25 s) or an identity-specific IRI (204.25 s; Extended Data Fig. 9f) and 60-s ITI-50% as predicted by the ICI (64.25 s) or IRI (128.5 s; Extended Data Fig. 10a).

To determine the total conditioning time until learning (Figs. 1h, 3h and 5b), the cumulative duration of all conditioning time (ITI + trial periods) from conditioning start up to, but not including, the trial period following the calculated ‘learned trial’ was summed for each individual animal.

For analysis of ITI lick rates (Extended Data Fig. 8g–k), lick rate was calculated from the period beginning with either the start of the session or the end of the prior consumption bout (consumption bout defined as the period from the first lick following reward delivery through all licks in which the interval between consecutive ‘lick off’ to ‘lick on’ was ≤500 ms) and ending with the onset of the following cue. ITI lick rate before learned trial was calculated as the median of the ITI lick rate for every ITI preceding the animal’s learned trial. Animals that did not show evidence of learning were excluded from this analysis. One additional 600-s ITI mouse with many long (>10-s) contacts with the spout during the ITI across days (presumed to be due to holding the spout) was also excluded from this analysis.

#### Cue–shock behavior

Our main behavioral measure for cue–shock conditioning was freezing during the shock-predictive cue. To analyze motion and freezing to the cue, top-down videos (640 × 480, ~30 fps) of each conditioning session were analyzed using ezTrack^69^. Empty-chamber calibration videos were used to determine a motion threshold noise cutoff of 11.5. Motion was calculated as the number of pixels with frame-to-frame grayscale value changes exceeding the motion threshold. Freezing was defined as at least 10 consecutive frames with motion below 500. To analyze freezing to the cue, the percentage of frames coded as ‘freezing’ from cue onset to offset was determined. We subtracted the baseline of the percentage of frames coded as freezing during a baseline time period equivalent to cue duration (15 s) immediately preceding cue onset. Motion to the cue was defined as the average motion from cue onset to offset and was similarly baseline subtracted. To determine the trial at which animals learned the cue–shock association, the same algorithm used to determine the learned trial in cue–reward conditioning was used on the cumsum of the freezing to cue, and this trial was used to calculate total conditioning time until learning, similarly to cue–reward conditioned mice (Extended Data Fig. 5b–d). Similarly to cue–reward conditioning, average cumsum curves are plotted on trial units scaled by the ratio of ISI to display curves as a function of conditioning time (Extended Data Fig. 5f,g).

#### Dopamine

To analyze the signals, a session-wide d*F/F* was calculated by applying a least-squares linear fit to the 405-nm signal to scale and align it to the 470-nm signal. The resulting fitted 405-nm signal was then used to normalize the 470-nm signal. Thus, d*F/F* is defined as d*F/F* = (470-nm signal − fitted 405-nm signal)/fitted 405-nm signal, expressed as a percentage^70^. Cue-evoked dopamine was measured as the area under the curve (AUC) of the dopamine signal for 0.5 s following cue onset minus the AUC of the baseline period 0.5 s directly preceding cue onset. Reward-evoked dopamine was measured as the AUC 0.5 s following the first detected lick after reward delivery minus the AUC of the pre-cue baseline period described above. If the onset and offset of a detected lick spanned reward delivery time, the reward AUC was calculated from time of reward delivery. For quantifying dopamine dips in response to omitted rewards (Fig. 8d–f), the AUC of a 2-s baseline window was subtracted from the AUC of a 2-s window beginning 1.25 s following cue onset (time of reward delivery in rewarded trials). A longer duration window was used to measure dips to account for the slower kinetics and broader shape of dips relative to cue responses. All dopamine responses reported in main figures are AUC measurements, but peak measurements are also plotted as a comparison point (Extended Data Figs. 4 and 6). To measure cue and reward peak dopamine responses, the mean dopamine signal during the baseline period was subtracted from the maximum value of the dopamine signal during the cue and reward windows described above for AUC measurements. Similarly to AUC measurements, peak responses were also normalized to the mean of the maximum three reward responses in each animal. To facilitate comparisons across mice with differing levels of virus expression, cue and reward dopamine measurements per mouse were normalized to the average of the three maximum reward responses in that mouse. For omission responses, dopamine measurements were normalized to the individual animal average of the three maximum reward responses recorded in a 2-s window following first lick after reward delivery to match the window used for dip measurements. All presented dopamine values represent these individual maximum reward normalized measurements, aside from example mice in which dopamine is plotted as %d*F/F* and cumsum plots in Figs. 2h and 7i and Extended Data Fig. 4c in which data are normalized to the maximum value of each animal’s cumsum curve as described below. Maximum rather than initial reward responses were chosen, as the reward response initially increased across early conditioning trials with different numbers of trials until maximum between conditions (Extended Data Figs. 4g,h and 6m,p).

To calculate the trial at which dopamine responses to the cue develop (dopamine learned trial), we took the cumsum of the normalized cue dopamine response described above. A diagonal was drawn from trial 1 through the point on the cumsum curve at 1.5 times the behavior learned trial to account for decreasing cue responses with extended training^71^. The same algorithm described above to determine the behavior learned trial was run on the cue dopamine curve. The lag between dopamine and behavioral learned trial (the number of trials between the development of dopamine responses to the cue and the emergence of behavioral learning) was defined as the behavior learned trial minus the dopamine learned trial (Fig. 2f). Omission dip learned trials (Fig. 8e,f) were calculated using the same algorithm on omission dip responses to detect the negative-going inflection point across all omission trials. One outlier 60-s ITI-10% mouse was excluded from the analysis due to consistent negative dopamine dips to the cue precluding our ability to detect the point at which the cue-evoked increase emerges (Extended Data Fig. 10j).

For cue–shock conditioned mice, the AUC during the last 14 s of the cue response was used as the main measure of cue-driven dopamine response (Extended Data Fig. 5j–l). The first second of the cue response was not included due to the presence of cue onset responses that were variable across animals and present on the first trial of conditioning. The dopamine learned trial was calculated for these animals using the same algorithm as the one used for cue–reward responses, but it was used to detect the negative-going inflection point in the cumsum curves due to the cue response evolving a dip during conditioning (Extended Data Fig. 5i–l). To average trial PSTHs across animals for cue–shock conditioned mice (Extended Data Fig. 5i), each animal’s PSTH was divided by the average of the three maximum peak values from trial onset through 2 s following shock termination. Two cue–shock conditioned mice (one 45-s ISI and one 135-s ISI) were excluded from dopamine analyses due to the absence of a consistent dip during the cue throughout conditioning (Extended Data Fig. 5l).

For one 60-s ITI dLight animal, during an initial conditioning session, a software crash caused the loss of lick data for 50 trials experienced by the animal. An additional 13 trials were presented to the animal that day and recorded following the crash. Photometry data were recorded for all 63 trials. Because the crash occurred before the emergence of learning and cue-evoked licking behavior (as confirmed by both online observation by experimenter before crash and a −0.14-Hz average cue-evoked change in lick rate for the 13 trials recorded after crash), the 50 trials in which data were lost were coded as 0 cue-evoked licks. All 63 trials the animal experienced were included in analyses.

To visualize the average relationship between dopamine responses and licking behavior across learning in 60-s and 600-s ITI mice with variability in individual learning rates, signals were aligned to the behavior learned trial and plotted through 250 or 25 trials after learning (Fig. 2h and Extended Data Fig. 4c). For aligned cumsum plots, data were normalized by the value from trial 400 (60-s ITI) or trial 40 (600-s ITI).

To quantify the relationship between dopaminergic learning rate and IRI (Extended Data Fig. 6e), the mean dopamine trials to learn 60-s and 600-s ITI groups were plotted against the IRI (mean ITI + 4.25 s (1.25-s trial period + 3-s consummatory period)) on a log–log plot, and the line between means was calculated as was done for behavior yielding the equation: log(trials_to_learn_dopamine) = (−1.0359)log(IRI) + 3.4338. The slope and intercept determined here were used to calculate the predicted trials or rewards to learn dopamine for 3,600-s ITI (3,604.25-s IRI; Extended Data Fig. 6i) and 60-s ITI-50% as predicted by the ICI (64.25 s) or IRI (128.5 s; Extended Data Fig. 10b).

### Theory and simulations

#### ANCCR: intuitive derivation of scaling of retrospective learning rate

We previously proposed a new learning model named ANCCR based on the learning of retrospective associations^35^. ANCCR operates by identifying cues consistently preceding meaningful events such as rewards. Thus, it learns whether a cue consistently precedes a reward (that is, a retrospective cue–reward association). This retrospective association provides a means to estimate whether the reward consistently follows a cue (that is, the prospective cue–reward association). The core principle of ANCCR is that a cue–reward association is learned and cached as a retrospective predecessor representation (denoted by *M*_*←*cr_), and then converted to a prospective successor representation (denoted by *M*_*→*cr_) using a Bayes’ rule-like normalization: ${M}_{\to {\rm{cr}}}={M}_{\leftarrow {\rm{cr}}}\frac{{M}_{\leftarrow r-}}{{M}_{\leftarrow c-}}$. *M*_*←r-*_ is proportional to the baseline rate of a reward in the environment, and *M*_*←c-*_ is proportional to the baseline rate of a cue. Here, we provide a quick intuitive derivation of the scaling of learning rate by the IRI. Please note that all the above variables are assumed to be conditioned on the experimental context. Thus, they should be listed as *M*_*←*cr|context_*, M*_*→*cr|context_*, M*_*←*c-|context_*, M*_*←*r-|context_. Because listing these conditional dependences is notationally cumbersome, we omit this in our treatments.

Each term on the right-hand side of the Bayes’ normalization (that is, *M*_*←*cr_, *M*_*←r-*_ and *M*_*←c-*_) is updated using a delta-rule-like update in ANCCR. Specifically, in equation (1):

1 $${M}_{\leftarrow {\rm{cr}}}\equiv (1-\alpha ){M}_{\leftarrow {\rm{cr}}}+{\alpha E}_{c};\text{updates at reward times}$$

where *E*_*c*_ is the eligibility trace of the cue, *α* is the learning rate for the retrospective update, and ≡ is the symbol for update, and equation (2):

2 $${M}_{\leftarrow x-}\equiv \left(1-{\alpha }_{0}\right){M}_{\leftarrow x-}+{\alpha }_{0}{E}_{x};\mathrm{updatesevery}dt$$

where *x* is any event type (for example, cue or reward) and *E*_*x*_ is its eligibility trace. As can be seen, *M*_*←*cr_ updates at the time of every reward with a learning rate of *α*, and *M*_*←x-*_ updates every *dt* (that is, continually) with a learning rate of *α*_0_. Both update rules determine a corresponding timescale of history for each variable (*M*_*←*cr_ or *M*_*←x-*_)—defined as the timescale over which a past event exerts influence on the current value of *M*_*←*cr_ or *M*_*←x-*_. The timescale over which one presentation of the cue influences future values of ${M}_{{\leftarrow {\rm{cr}}}_{,}}$ that is, its timescale of history, depends on both *α* and how frequently the reward occurs. On the other hand, the corresponding timescale for *M*_*←x-*_ depends on *α*_*0*_ and *dt*.

For the Bayes’ rule-like normalization to work in a (possibly) nonstationary environment, all terms on the right-hand side (that is, *M*_*←*cr_, *M*_*←r-*_ and *M*_*←c-*_) should be calculated over the same timescale of history. This is because normalizing the predecessor representation calculated over 1 h (say) by baseline rates of reward and cue calculated over 1 min (say) is obviously inappropriate if the environment has the potential to change during the hour. Thus, the quantitative relationship between learning rate and IRI can be obtained by setting the timescale of history for *M*_*←*cr_, *M*_*←r-*_ and *M*_*←c-*_ to be equal to each other. As shown in equations (1) and (2), the baseline rates of reward and cue are updated by a delta rule with a baseline learning rate *α*_*o*_ continually (that is, every time step *dt*). Assuming that the time constant of decay of the eligibility trace is very short, a single occurrence of *x* will have a net influence on *M*_*←x-*_ of ${\left(1-{\alpha }_{0}\right)}^{n}$ after *n* time steps—an exponentially decaying function of time. Equating this influence with an exponential time decay of ${e}^{-\frac{t}{\tau }}$, one can calculate the time constant of decay as $\tau =\frac{-{dt}}{\mathrm{ln}\left(1-{\alpha }_{0}\right)}$. Thus, the net timescale of history for the calculation of the baseline rate of events (cues or rewards) is $\frac{-{dt}}{\mathrm{ln}\left(1-{\alpha }_{0}\right)}$, where the numerator is the time interval between consecutive delta-rule updates with learning rate *α*_*o*_. The timescale of history for the predecessor representation *M*_*←*cr_ has a similar expression with the numerator being the time interval between consecutive updates, which equals the IRI because updates only occur at reward times, and the learning rate in the denominator equals the learning rate of associative update, *α*. Thus, the timescale of history for the predecessor representation is $\frac{-{\rm{IRI}}}{{\rm{ln}}(1-\alpha )}$. Setting both these timescales of history to be equal, one can show that the learning rate of associative update should be $\alpha =1-{(1-{\alpha }_{0})}^{\frac{{\rm{IRI}}}{{\rm{dt}}}}$. For small learning rates, this expression simplifies to $\alpha ={\alpha }_{0}\frac{{\rm{IRI}}}{{\rm{dt}}}$.

For a more formal derivation that accounts for the time constant of eligibility trace^72^, see Supplementary Note 1.

#### Comparison of learning models

To determine if a model of associative learning could capture the experimentally observed proportional scaling across ITI conditions, we simulated three likely candidates, which all account in some way for the time between cue–reward trial experiences: the microstimulus implementation of TDRL^41^, Wagner’s SOP^42,43^ and ANCCR^35^. For each model, we simulated the experimental conditions for 30 s through 3,600-s ITI and tested combinations of parameters to determine which could best replicate the quantitative, proportional scaling of learning rate by IRI observed experimentally. Our measure against which specific model instances were compared was the ‘trials to learn’ for each of the 30-s, 60-s, 300-s, 600-s and 3,600-s ITI groups. Simulations of each model were based on published versions^35,41,43^ with adjustments to ANCCR described above and below. To generate behavior, all models assumed that behavior emerged following a threshold crossing by the association quantity, which corresponded to cue ‘value’ in both TDRL and SOP and net contingency (NC_cue⟷reward_) in ANCCR. Thus, ‘learned trial’ is defined in TDRL and SOP as the first trial when value > threshold, and in ANCCR it is defined as the first trial where net contingency > threshold. While we recognize that action selection would likely involve other processes, the above threshold crossing was implemented to ensure that the models generated ‘learned trials’ through comparable operations. For each combination of parameters for all three models, all five ITI conditions (30 s–3,600 s) were simulated for the number of trials that experimental animals experienced over 8 days of conditioning. Each case was iterated 20 times.

To determine the parameter combination from each model that best fit the experimental data, we calculated the residual sum of squares (RSS) of the trials to learn from each simulated parameter combination against the experimental trials to learn for each IRI (Fig. 3g). RSS was calculated on log-transformed data to account for the wide variation in trials to learn across IRIs. The simulation with the lowest RSS was deemed the best fit to the experimental results.

After determining the best-fit parameter combination for each model, TDRL, SOP and ANCCR, we measured the AIC as AIC = 2*k* + *n* × ln(meanRSS), where *n* = sample size (number of animals), *k* = the number of parameters in the model, and meanRSS = the mean RSS between trials to learn for that parameter combination and experimental data. A lower AIC value represents a better fit to the data accounting for number of parameters needed to fit. Note that all data presented in figures (Extended Data Fig. 7) and text and used to calculate model weights represent the AIC calculated in which models are not penalized for parameters by substituting *k* = 0 into the equation above. This yields the formula AIC = *n* × ln(meanRSS). This was implemented as a conservative measure because only a few parameters from most models had the potential to affect the simulated results and the best-fit model (ANCCR) is the one with the fewest parameters. For AIC values penalizing the total number of parameters per model, refer to Supplementary Table 1.

The best model between TDRL, SOP and ANCCR was then determined as the one with the minimum AIC. A relative weight for each model compared to the best model was then calculated as model weight = *e* ^−0.5^^×(AIC – AIC^_min_^)^. For additional simulation results presented in Extended Data Fig. 7, model weights were calculated using the best-fit AIC from the other two models presented in Fig. 5.

To determine whether the time to learn increased with increasing ITIs in each model, the time to learn for each ITI condition for each simulation was calculated by multiplying the number of trials and the number of ITIs experienced before the first trial following the learned trial by the trial duration and mean ITI duration, respectively, and summing those numbers (Fig. 5e,h,k and Extended Data Fig. 7d,f). To determine if the time to learn for each ITI condition increased with increasing ITI duration, a regression was fit to the time to learn for the 30-s through 600-s ITI groups and slopes were compared to a similar regression fit through the behavior data (Extended Data Fig. 7a).

##### TDRL simulations

TDRL assumes that animals assign value to each moment following an event (for example, cue) to predict future reward. Each event elicits multiple states, and the value of each time step can be expressed as a weighted sum of activated states at that moment. If the prediction from the previous moment is different from what is experienced in the current moment, the model updates the value of the previous moment based on this RPE, assumed to be signaled by dopamine. Depending on how the model represents a state, TDRL can be further divided into subtypes. Here, for a representative TDRL algorithm, we used the microstimulus model^41^ because it naturally accounts for the ITI. This model assumes that time states are Gaussian functions of increasing width following each event (cue or reward). The following model parameters were fixed for every iteration: bin size (*dt*) = 0.25 s (set to cue duration); decay parameter of eligibility trace (λ) = 0.99 (set to a high value to allow rapid credit assignment to earlier states); width of Gaussian function (σ) = 0.08. For the following parameters, we swept across a range to determine whether any combination could best explain proportional IRI scaling of learning rate: threshold for behavior generation = [0.1, 0.2, 0.3, 0.4, 0.5, 0.6, 0.7, 0.8]; decay parameter of event memory (d) = [0.8, 0.9, 0.99, 0.999, 0.9999]; temporal discounting factor (γ) = [0.8, 0.9, 0.99, 0.999, 0.9999]; number of states elicited by each event (m) = [3, 10, 100, 1,000]; learning rate (α) = [0.001, 0.01, 0.1]. The best-fit-to-behavior parameter combination (Fig. 5c–e and Extended Data Fig. 7b) was: threshold = 0.3, α = 0.1, γ = 0.99, m = 3 and d = 0.9.

For comparisons between models (Fig. 5 and Extended Data Fig. 7), simulations were run for the same number of trials as experimental groups (30 s: 800, 60 s: 400, 300 s: 88, 600 s: 48, 3,600 s: 16). Because value and RPE did not asymptote in all ITI conditions for the best-fit model when simulating the same number of trials as experimental groups, we again ran the simulation with the best-fit parameters for at least 400 trials per ITI group to determine the asymptotic levels of RPE and value. We also searched for the best-fit parameter combination (Extended Data Fig. 7c) when ITI conditions 60 s–3,600 s were run for at least 400 trials. The best-fit TDRL parameter combination when each ITI consisted of at least 400 trials per group was: threshold = 0.4, α = 0.10, γ = 0.80, m = 3, d = 0.999. AIC and model weight comparisons for this model were run against the best-fit SOP and ANCCR models (from Fig. 5f–k and Extended Data Fig. 7e,g).

In principle, a similar rule derived in ANCCR could be applied ad hoc to any model of associative learning. Here, we demonstrate that applying such a rule to TDRL improves fit to experimental results. Using the best-fit model parameters determined during the initial TDRL parameter sweep described above (Fig. 5c–e and Extended Data Fig. 7b), we replaced the learning rate, *α*, based on the equation *α* = 1 – *e* ^(−*k*· IRI)^ and performed another parameter sweep to determine the best fit *k*. We searched over the range *k* = [0.00015, 0.0002, 0.00025, 0.0003, 0.00035, 0.0004] (the range matching experimentally observed learning rate) and found that the best fit to behavior results from *k* = 0.0003 (Extended Data Fig. 7d). Because value and RPE did not asymptote in all ITI conditions for the best-fit model when simulating the same number of trials as experimental groups, we again ran the simulation with the same parameters for 2,400 trials in total to determine the asymptotic cue-evoked RPE for this parameter combination.

##### SOP simulations

In SOP, cues or rewards evoke processing nodes consisting of many elements. These stimulus representations are dynamic: presentation of a stimulus moves a portion of elements from (only) the inactive (I) state into the primary active state (A1). Elements then decay into the secondary active state (A2; a refractory state) and then decay again back to the inactive state while the stimulus is absent. Elements transition between states according to individually specified probabilities. Cue–reward associations (value) are strengthened and learned when cue elements in A1cue and reward elements in A1reward overlap in time and decreased when cue elements in A1cue and reward elements in A2reward overlap in time. Following learning, cues evoke conditioned responding by directly activating reward elements to their A2 state. One way in which SOP has been hypothesized to explain ITI impact on learning is that more time between trials allows more elements to decay to the inactive state (as opposed to the refractory A2 state), allowing for a greater number of elements to transition to the A1 active state upon next cue and reward presentation. Parameter combinations were swept through to determine if any set of parameters could capture the quantitative scaling observed in the experimental results.

The relevant parameters in the model controlling the transition probabilities from I- > A1- > A2- > I are p1, pd1 and pd2, respectively. p1_cs_, pd1_cs_ and pd2_cs_ refer to the transition probabilities controlling the cue representation, while p1_us_, pd1_us_ and pd2_us_ refer to the transition probabilities controlling transitions between reward representation active states. The following parameters were fixed for every iteration of SOP run: the time step (*dt*) = 0.25 s (set to cue duration); reward magnitude of CS in A1 (r1) = 1; reward magnitude of CS in A2 (r2) = 0.5; scale factor for magnitude of activation for coincidence of CS and unconditioned stimulus (US) in A1(*L*_plus_) = 0.2; scale factor for magnitude of inhibition for coincidence of CS in A1 and US in A2(*L*_minus_) = 0.1; and p1cs = 0.1 and p1us = 0.6 based on previously published work^43^. The following parameter combinations reflecting the variables hypothesized to drive trial spacing effect were varied: threshold for behavior generation = [0.01, 0.1, 0.2, 0.3, 0.4, 0.5, 0.6, 0.7, 0.8]; pd1_us_ = [0.01, 0.2, 0.25, 0.5, 0.75]; pd2_us_ = [0.001, 0.001, 0.01, 0.1]; pd1_cs_ = [0.01, 0.2, 0.25, 0.5, 0.75]; pd2_cs_ = [0.001, 0.001, 0.01, 0.1]. Because SOP implementations make the assumption that pd1 > pd2 (ref. ^43^; that is, the decay from the A1 active state to the A2 state should be faster than the decay from the A2 active state to the inactive state), we constrained our results to parameter combinations that satisfied this inequality. The parameter combination providing the best fit to behavior (Fig. 5f–h and Extended Data Fig. 7e) was: threshold = 0.1, pd1_us_ = 0.25, pd2_us_ = 0.1, pd1_cs_ = 0.1 and pd2_cs_ = 0.0001. Relaxing this constraint, the best model fit parameters were: threshold = 0.6, pd1_us_ = 0.01, pd2_us_ = 0.01, pd1_cs_ = 0.1 and pd2_cs_ = 0.0001 (Extended Data Fig. 7f).

##### ANCCR simulations

In ANCCR, we derived a scaling rule for the retrospective learning rate (*α*) and the eligibility trace time constant (*T*) from the core principle of Bayes’ rule conversion of a retrospective to a prospective association (Supplementary Note 1). For the simulations considered here, these rules simplify to: $\alpha =1-{(1-{\alpha }_{0})}^{\frac{{\rm{IRI}}}{{dt}}}$ and *T* = *k* *×* IRI. For ANCCR, the parameters that were swept to identify the best-fit model were: threshold = [0.1, 0.2, 0.3, 0.4, 0.5, 0.6, 0.7, 0.8] *α*_*0* _= [1 × 10^−4^, 8 × 10^−5^, 6 × 10^−5^, 4 × 10^−5^, 2 × 10^−5^, 1 × 10^−5^, 8 × 10^−6^], *k* = [0.1, 0.3, 0.5, 0.7]. The best-fit parameters were: threshold = 0.4; *α*_*0*_ = 4 × 10^−5^; *k* = 0.5. The following parameters were fixed: *w* = 0.5; *dt* = 0.2 (same as in ref. ^35^). The dopamine response to the first reward was relatively high (although this response increases with repeated reward experience, consistent with our previous demonstration^35^). Two possibilities exist to account for this. One is that there is a Bayesian prior for *M*_*r←r*_ and *M*_*r←-*_, and the other is that part of the innate meaningfulness of a reward is signaled by dopamine. For simplicity, we assumed the latter and added an innate meaningfulness of 1 to dopamine reward response and 0 to dopamine cue response.

### Statistics and reproducibility

No statistical test was used to predetermine sample sizes. Sample sizes were chosen based on *n* values in similar published studies. Blinding was not possible during data acquisition because experimenters had to use specific conditioning protocols based on grouping. Experimenters were not blind to groups during data analysis, but were blind to group identity during histology for fiber placement verification. Animals excluded from specific analyses are described above and noted in figure legends. Statistical analyses were performed in Python 3.12 using either the scipy.stats (v1.16.2) or Pingouin^73^ (v0.5.5) packages. Welch’s *t*-test and Welch’s ANOVA was performed to compare between experimental groups, so as to not assume equal variances between the populations (Fig. 1g,j,k). To test for equality of variances, *F*-tests were run using a custom script. Nonparametric tests (Kruskal–Wallis *H*, Mann–Whitney *U*) were used to compare simulation results due to the presence of conditions with 0 variance and for learned trial comparisons with the 60-s-10% group due to the skewed distribution of their data. For the eight experimental comparisons performed in Fig. 6, the false discovery rate was controlled using the Benjamini–Yakutieli method to adjust *P* values. For comparison of asymptotic dopamine levels (Fig. 4j) and comparison of regression slopes for time to learn (Extended Data Fig. 7a), a Benjamini–Hochberg procedure was used to adjust *P* values. All other multiple comparisons were corrected for by adjusting *P* values with Bonferroni’s correction (Fig. 7j and Extended Data Figs. 7, 8b, 9b,f and 10a,b,k). All statistical tests were two tailed. *N* values reported represent individual animals or, in the case of simulations, the number of iterations. All linear regressions presented fit with a least-squares method using the ‘scipy.stats.linregress’ function (Fig. 3g and Extended Data Figs. 6e, 7a and 8i–k). For sigmoid fits to cue and omission dopamine responses (Fig. 8d), ‘scipy.optimize.curve_fit’ was used to determine parameters that best fit data to the equation *y* = *L* / (1 + np.exp(−*k* × (*x* − *x*_0_))) + *b*. Full statistical test information is presented in Supplementary Table 1, including test statistics, *n* values, degrees of freedom and both corrected and uncorrected *P* values. Time courses of the cumsum or average of the lick and/or dopamine data are presented as the mean between animals/iterations ± s.e.m. Bar graphs are presented as the mean between animals/iterations ± s.e.m. with individual animal (or iteration) data points. In the box plots (Fig. 7j and Extended Data Fig. 10k), the line represents the median, box edges represent the IQR, and whiskers extend to data within 1.5 times the IQR from the box. Results were considered significant at an alpha of 0.05. *P < 0.05, **P < 0.01, ***P < 0.001, ****P < 0.0001; NS (nonsignificant) denotes *P* > 0.05.

### Reporting summary

Further information on research design is available in the Nature Portfolio Reporting Summary linked to this article.

## Online content

Any methods, additional references, Nature Portfolio reporting summaries, source data, extended data, supplementary information, acknowledgements, peer review information; details of author contributions and competing interests; and statements of data and code availability are available at 10.1038/s41593-026-02206-2.

## Supplementary information

Supplementary Information Supplementary Note 1 and Supplementary Table 1.

Reporting Summary

## Data Availability

Data underlying this study is publicly available on DANDI at https://dandiarchive.org/dandiset/001632/.

## Code availability

All code for generating figures, analyzing data and running simulations is available at https://github.com/namboodirilab/duration_between_rewards_controls_learning_rate/.
