# Publisher Correction: Duration between rewards controls the rate of behavioral and dopaminergic learning

**DOI:** 10.1038/s41593-026-02425-7

**Published:** 2026-08-03

**Authors:** Dennis A. Burke, Annie Taylor, Huijeong Jeong, SeulAh Lee, Leo Zsembik, Brenda Wu, Joseph R. Floeder, Gautam A. Naik, Ritchie Chen, Vijay Mohan K Namboodiri

**Affiliations:** 1 Department of Neurology, University of California, San Francisco, San Francisco, CA USA; 2 Neuroscience Graduate Program, University of California, San Francisco, San Francisco, CA USA; 3 University of California, Berkeley, Berkeley, CA USA; 4 Department of Neurological Surgery, Department of Psychiatry and Behavioral Sciences, Department of Bioengineering and Therapeutic Sciences, University of California, San Francisco, San Francisco, CA USA; 5 Weill Institute for Neurosciences, University of California, San Francisco, San Francisco, CA USA; 6 Kavli Institute for Fundamental Neuroscience, Center for Integrative Neuroscience, University of California, San Francisco, San Francisco, CA USA

**Keywords:** Reward, Classical conditioning

Correction to: *Nature Neuroscience* https://doi.org/10.1038/s41593-026-02206-2, published online 12 February 2026.

In the version of the article initially published, the raster plots in Fig. 4b (top row) were misaligned and have now been corrected in the HTML and PDF versions of the article, as seen in Fig. 1.

**Fig. 1:**
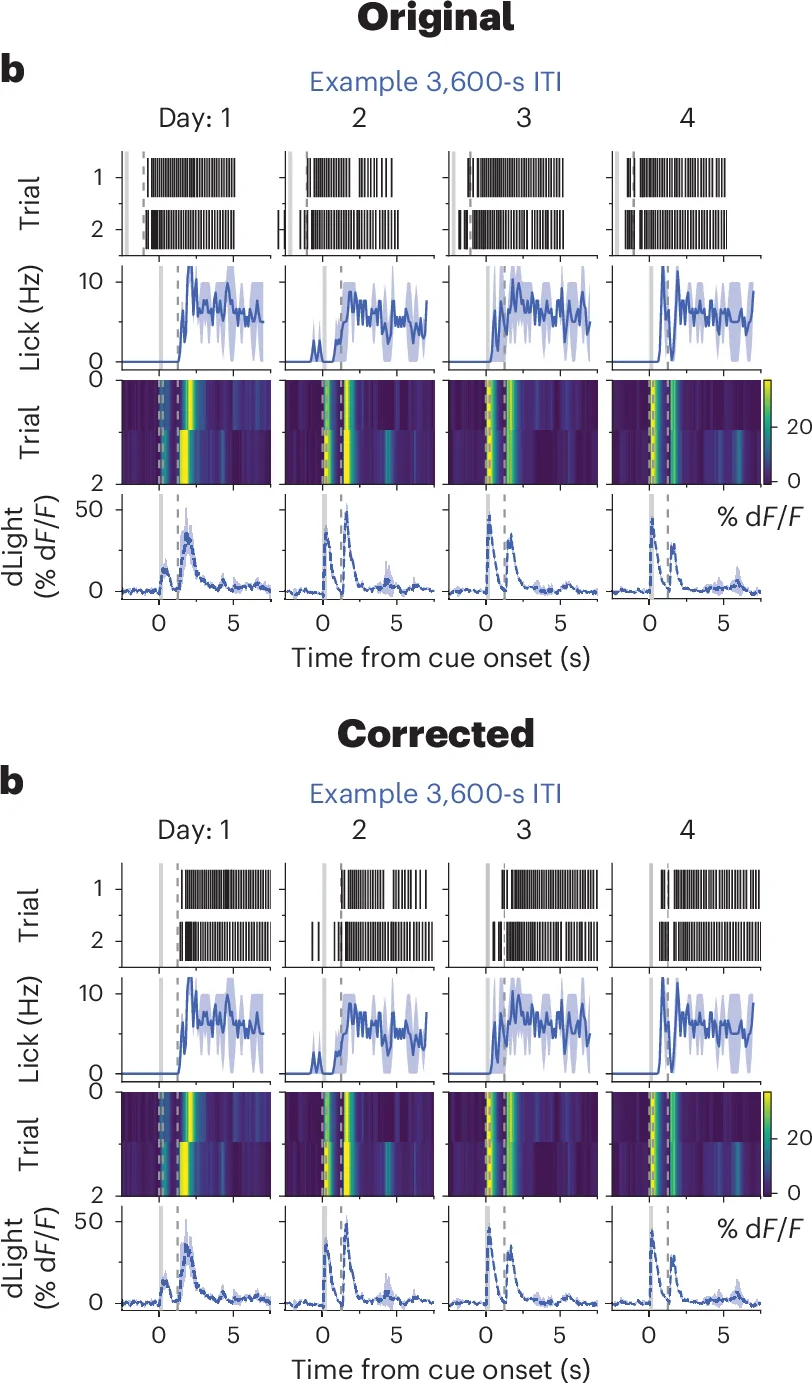
**Original and corrected Fig. 4b.**

