# Supplementary material for: Duration between rewards controls the rate of behavioral and dopaminergic learning
Source: Nat Neurosci. 2026 Feb 12;29(4):825–39. doi: 10.1038/s41593-026-02206-2 (PMC13035403; doi:10.1038/s41593-026-02206-2)
Supplement: Supplementary file 1 — Supplementary Note 1 and Supplementary Table 1. [file 41593_2026_2206_MOESM1_ESM.pdf]

nature neuroscience

Supplementary information https://doi.org/10.1038/s41593-026-02206-2

Duration between rewards controls the rate of behavioral and dopaminergic learning

In the format provided by the authors and unedited

Supplementary Note 1

Formal derivation of proportional scaling of retrospective learning rate by inter-reward interval

In the intuitive derivation above, we made the critical assumption that the time constant of decay of the eligibility trace, *T,* is very short. Here, we will derive the expression for the timescale of history for $M_{\leftarrow cr}$ and $M_{\leftarrow r-}$ or $M_{\leftarrow c-}$ for a general *T* and show that they are $\frac{-IRI}{\ln(1-\alpha)}$ and $\frac{-dt}{\ln(1-\alpha_0)}$ respectively, provided α_0 [illegible] $1-e^{-\frac{dt}{T}}$ (the formal condition of "very short" *T*, which as it turns out, need not be very short if α_0 is low).

We will first calculate the timescale of history for $M_{\leftarrow r-}$ or $M_{\leftarrow c-}$, both of which are updated using successive delta-rule like updates every *dt* with learning rate α_o. To this end, we will consider an event train of event type x (either cue or reward) and denote the estimate of $M_{\leftarrow x-}$ at the time of the nth timepoint/sample (i.e., at time *ndt*) as *M*(*n*) and the corresponding instantaneous eligibility trace as *E(n)*. The delta-rule for update of *M(n)* can then be written as:

$$M(n) = M(n-1) + \alpha_0\left(E(n) - M(n-1)\right) \tag{1}$$

This update occurs every *dt*. Simplifying, we get

$$M(n) = (1-\alpha_0)M(n-1) + \alpha_0 E(n) \tag{2}$$

Similarly expanding *M(n-1)* in terms of *M(n-2)*, we get

$$M(n) = (1-\alpha_0)\left[(1-\alpha_0)M(n-2) + \alpha_0 E(n-1)\right] + \alpha_0 E(n) \tag{3}$$

Expanding this recursive expression till *M(0)*, i.e., the initialized value of $M_{\leftarrow x-}$ prior to the first sample/timepoint, we get

$$M(n) = (1-\alpha_0)^n M(0) + \alpha_0\left[\sum_{i=0}^{n-1}\left\{(1-\alpha_0)^i E(n-i)\right\}\right] \tag{4}$$

We will assume that *M(0)=0* and replace *i* with *n-i* to count from the first sample to get

$$M(n) = \alpha_0\left[\sum_{i=1}^{n}\left\{(1-\alpha_0)^{n-i} E(i)\right\}\right] \tag{5}$$

To calculate the timescale of history of $M_{\leftarrow x-}$, we now just need to substitute an expression of *E(i)* for a given event train of event type x, and then identify the exponential time constant of growth.

We will next show that the eligibility trace for an event train occurring at a constant rate with period t_x rides on a trendline that is a saturating exponential growth curve with a time constant equal to the eligibility trace decay time constant, *T*. In other words, the growth curve of the eligibility trace is $\frac{T}{t_x}\left(1-e^{-\frac{t}{T}}\right)$.

To this end, we will assume a periodic event train for x from here on for analytical simplicity. Despite this simplistic assumption, our simulations show that the key results from this derivation hold for more realistic event trains.

Assume that the period of this periodic event train is t_x and that just before the jth occurrence of event x, the eligibility trace is E_j. If so, by definition, the eligibility trace immediately after the occurrence of the jth occurrence is E_j+1. Therefore, the mean eligibility trace in the time between the jth and $j+1^{th}$ occurrences, denoted as $\overline{E}_j$ is by definition:

$$\overline{E}_j = \frac{\int_0^{t_x} \left(E_j + 1\right) e^{-\frac{t}{T}} dt}{t_x} = \frac{T}{t_x}\left(E_j + 1\right)\left[1 - e^{-\frac{t_x}{T}}\right] \tag{6}$$

For a periodic event train, E_j can be calculated as

$$E_j = e^{-\frac{t_x}{T}} + e^{-\frac{2t_x}{T}} + \ldots + e^{-\frac{(j-1)t_x}{T}} = \frac{e^{-\frac{t_x}{T}} - e^{-\frac{jt_x}{T}}}{1 - e^{-\frac{t_x}{T}}} \tag{7}$$

Substituting this into the previous equation, we see that

$$\overline{E}_j = \frac{T}{t_x}\left(1 - e^{-\frac{jt_x}{T}}\right) \tag{8}$$

This means that the average trendline of the eligibility trace grows as a saturating exponential curve with time constant T and asymptote T/t_x. An interpolated continuous time estimate of the above trendline is simply $\frac{T}{t_x}\left(1 - e^{-\frac{t}{T}}\right)$. The exact eligibility trace curve will see-saw around this trendline as it grows by 1 on every occurrence of the event and then decreases exponentially until the next occurrence. However, since our goal here is to just find the overall timescale of growth for the mean eligibility trace $M_{\leftarrow x\text{-}}$, it is sufficient to substitute the above interpolated trendline for the eligibility trace into Equation (5).

Thus, at the ith sample in Equation (5), $E(i)$ can be written as $E(i) = \frac{T}{t_x}\left(1 - e^{-\frac{idt}{T}}\right)$. Please note that the *i* here refers to the ith sample and not the complex square root of -1.

Therefore, $M(n)$ from Equation (5) can be rewritten as:

$$M(n) = \frac{\alpha_0 T}{t_x}\left(1 - \alpha_0\right)^n \left[\sum_{i=1}^{n} \frac{\left(1 - e^{-\frac{idt}{T}}\right)}{\left(1 - \alpha_0\right)^i}\right] \tag{9}$$

It can be shown that this simplifies to

$$M(n) = \frac{T}{t_x} \frac{\left(1 - e^{-\frac{dt}{T}}\right)\left[1 - \left(1 - \alpha_0\right)^n\right] - \alpha_0\left[1 - e^{-\frac{(n+1)dt}{T}}\right]}{\left(1 - e^{-\frac{dt}{T}}\right) - \alpha_0} \tag{10}$$

This equation shows the growth curve for the average estimate of the trendline of the eligibility trace of event x. It can be verified that when α_0 is set to 1 (i.e., there is no averaging), the above curve reduces to the trendline of the eligibility trace that we assumed earlier, i.e., $\frac{T}{t_x}\left(1 - e^{-\frac{ndt}{T}}\right)$. When α_0 [illegible] $1 - e^{-\frac{dt}{T}}$ (i.e., eligibility trace decays quickly and there is slow averaging of eligibility trace), the first term in the numerator and denominator dominate, and therefore $M_{\leftarrow x\text{-}}$ grows as a saturating exponential curve of the form $\left[1 - \left(1 - \alpha_0\right)^n\right]$. Equating $\left[1 - \left(1 - \alpha_0\right)^n\right]$

with a saturating exponential growth curve of the form $1-e^{-\frac{t}{\tau}}$, we can see that the time constant equals $\tau = \frac{-dt}{\ln(1-\alpha_0)}$. Notice that this is also the same time constant that measures the exponentially decaying influence with which an event that occurred in the past influences the current estimate of $M_{\leftarrow x-}$. This is thus the timescale of history for $M_{\leftarrow x-}$.

We can now similarly estimate the time constant of growth for the predecessor representation $M_{\leftarrow xy}$ (typically, x is a cue and y is a reward). Intuitively, one can infer this from the above time constant for $M_{\leftarrow x-}$ of $\frac{-dt}{\ln(1-\alpha_0)}$ by noting that the numerator measures the time interval between successive delta-rule updates and the alpha in the denominator measures the corresponding learning rate for the delta-rule. By similarity, the interval between successive delta-rule updates for $M_{\leftarrow xy}$ is the inter-y-interval (denoted IYI) and the corresponding learning rate is α. Therefore, we can intuitively infer that the corresponding time constant should be $\tau = \frac{-IYI}{\ln(1-\alpha)}$. To see this more formally, let us assume a periodic event train for y with x's occurring just prior to each occurrence of y. In this case, if one sets the continual sampling to occur with $dt=IYI$, the baseline updates for x will be identical in timing to the predecessor representation updates calculated when y occurs. The only difference will be that the delta-rule updates for predecessor representation will have a learning rate α instead of α_0. Thus, the same results as in Equation (10) will hold for the predecessor representation, but with dt set to IYI and α_0 set to α. This means that the corresponding time constant of growth for the predecessor representation is $\tau = \frac{-IYI}{\ln(1-\alpha)}$.

Collectively, we can now see that to obtain the same time constant of growth for the baseline and the predecessor representation, one needs to satisfy the following relationship:

$$\frac{-dt}{\ln(1-\alpha_0)} = \frac{-IYI}{\ln(1-\alpha)} \tag{11}$$

This implies that α should be set to

$$\alpha = 1-(1-\alpha_0)^{\frac{IYI}{dt}} \tag{12}$$

For calculating the predecessor representation between a cue and reward, the retrospective updates occur at reward and hence, the corresponding learning rate should be:

$$\alpha = 1-(1-\alpha_0)^{\frac{IRI}{dt}} \tag{13}$$

Notice that the *IRI* here refers to the inter-reward interval of the specific type of reward, and is thus, reward identity-specific, a prediction that is tested in **Fig. 6j-l**.

Setting of eligibility trace time constant

It is intuitively clear that the eligibility trace time constant T needs to be set to match the timescales operating in the environment. This is because if the eligibility trace decays too quickly, there will be no memory of past events, and if it decays too slowly, it will take a long time to correctly learn event rates in the environment. Further, as we showed in the previous section, the asymptotic value of $M_{\leftarrow x-}$ for an event train at a constant rate λ_x with average period t_x is $T/t_x=T\lambda_x$. This means that the neural representation of $M_{\leftarrow x-}$ will need to be very high if T is very high and very low if T is very low. Since every known neural encoding scheme is non-linear at its limits with a floor and ceiling effect (e.g., firing rates can't be below zero or be infinitely high), the limited neural resource in the linear regime should be used appropriately for efficient coding. A linear regime of operation for $M_{\leftarrow x-}$ is especially

important in ANCCR since the estimation of the successor representation by Bayes' rule depends on the ratio of $M_{\leftarrow x-}$ for different event types. Such a ratio will be highly biased if the neural representation of $M_{\leftarrow x-}$ is in its non-linear range. Assuming without loss of generality that the optimal value of $M_{\leftarrow x-}$ is M_{opt} for efficient linear coding, we can define a simple optimality criterion for the eligibility trace time constant T. Specifically, we postulate that the net sum of squared deviations of $M_{\leftarrow x-}$ from M_{opt} for all event types should be minimized at the optimal T. The net sum of squared deviations, denoted by SS, can be written as

$$SS = \sum_x \left(M_{\leftarrow x-} - M_{opt} \right)^2 = \sum_x \left(T\lambda_x - M_{opt} \right)^2 \tag{14}$$

Where the second equality assumes asymptotic values of $M_{\leftarrow x-}$. The minimum of SS with respect to T will occur when $\frac{\partial(SS)}{\partial T} = 0$. It is easy to show that this means that the optimal T is:

$$T_{opt} = M_{opt} \frac{\sum_x \lambda_x}{\sum_x \lambda_x^{\,2}} \tag{15}$$

For typical cue-reward experiments with each cue predicting reward at 100% probability, $\lambda_{cue} = \lambda_{reward} = 1/IRI$. Substituting into the above equation, we get:

$$T_{opt} = M_{opt}.IRI \tag{16}$$

Thus, in typical experiments with 100% reward probability, the eligibility trace time constant should be proportional to the IRI (or equivalently, the inter-cue interval). In the simulations of ANCCR in **Fig. 5**, we used Equations (13) and (16) to set the learning rate and eligibility trace time constant respectively. These relationships explain why the learning rate scaling for a one-hour IRI deviates from perfect linearity in **Fig. 5**. This is because the optimal T for one-hour IRI equaled 1800 s (since best-fit M_{opt} was 0.5). At this value, the best-fit α_0 of $4\text{x}10^{-5}$ was not much smaller than $1-e^{-\frac{dt}{T}}$ $=11\text{x}10^{-5}$, and thus, the overall learning of the baseline $M_{\leftarrow c-}$ and the predecessor representation $M_{\leftarrow cr}$ was slower than the linear rate for the lower IRIs (see Equation (10)).

Both Equations (13) and (15) require the real-time estimation of inter-event intervals, which should be learned separately by an animal. Since the goal of the current study was not to understand how an animal achieves the real-time learning of inter-event intervals, we simply assumed the correct rates of the events in the simulations of ANCCR. Additional theoretical and experimental work will be needed to better understand how these quantities are set in real-time.

Supplementary Table 1

Fig.		Group (N)	Description	Test	Result	two-tailed p-value [uncorrected p-val]	Sig.
Fig. 1	g	**60 s ITI** (n = 17) **600 s ITI** (n = 19)	number of trials to learn	Welch's t-test	t (16.26) = -12.64	p = 7.90×10^{-10}	****
Fig. 1	g	**60 s ITI** (n = 17) **600 s ITI** (n = 19)	number of trials to learn: variance	F-test	F (16,18) = 111.2	p = 2.18×10^{-14}	****
Fig. 1	h	**60 s ITI** (n = 17) **600 s ITI** (n = 19)	total conditioning time before cue licking	Welch's t-test	t (31.97) = 0.39	p = 0.70	ns
Fig. 1	k	**60 s ITI** (n = 19) **600 s ITI** (n = 19)	change in lick rate to cue, last 10 or 100 trials	Welch's t-test	t (25.94) = 0.44	p = 0.66	ns
Fig. 1	k	**60 s ITI** (n = 19) **600 s ITI** (n = 19)	change in lick rate to cue, last 10 or 100 trials: variance	F-test	F (18,18) = 4.3	p = 0.00335	**
Fig. 2	e	**60 s ITI** (n = 5) **600 s ITI** (n = 7)	trials to DA learning	Welch's t-test	t (4.02) = 4.46	p = 0.011	*
Fig. 2	f	**60 s ITI** (n = 5) **600 s ITI** (n = 7)	trials from DA to behavior	Welch's t-test	t (4.06) = 7.20	p = 0.00184	**
Fig. 2	g	**60 s ITI** (n = 5) **600 s ITI** (n = 7)	total conditioning time before cue DA	Welch's t-test	t (6.27) = -0.87	p = 0.41	ns
Fig. 3	e	**30 s ITI** (n = 5) **300 s ITI** (n = 6)	number of trials to learn	Welch's t-test	t (4.1) = 4.36	p = 0.0114	*
Fig. 3	g	**mean trials to learn: 30 s, 60 s, 300 s, and 600 s ITI groups** (n = 4)	mean trials to learn (beh.) as a function of IRI (slope diff. from 0)	linear regression	log(trials_to_learn) = **(-1.0593)***log(IRI) + **3.8753** R2 = 0.9992	p = 0.000396	***
			slope of linear regression diff. from -1	one sample t-test	t(2) = -2.81	p = 0.106	ns
Fig. 3	h	**30 s ITI** (n = 5) **60 s ITI** (n = 17) **300 s ITI** (n = 6) **600 s ITI** (n = 19)	total conditioning time before cue licking	Welch's ANOVA	F(3,11.20) = 0.127	p = 0.94	ns

Fig. 4	e-f	**3600 s ITI** (n = 5)	number of trials to learn observed different from predicted (1.281)	one sample t-test	t(4) = 9.47	p =0.00069	***
Fig. 4	j	**60 s ITI** (n = 5)<br>**600 s ITI** (n = 7)	cue DA, last 10 or 100 trials	Welch's t-test	t (7.23) = -2.61	p = 0.0338<br>[0.0338]	*
Fig. 4	j	**3600 s ITI** (n = 5)<br>**60 s ITI** (n = 5)	cue DA, last 2 or 100 trials	Welch's t-test	t(4.42) = 7.72	p = 0.00199<br>[0.00100]	**
Fig. 4	j	**3600 s ITI** (n = 5)<br>**600 s ITI** (n = 7)	cue DA, last 2 or 10 trials	Welch's t-test	t(7.63) = 4.93	p = 0.00199<br>[0.00133]	**
Fig. 5	b	**30 s ITI** (n = 5)<br>**60 s ITI** (n = 17)<br>**300 s ITI** (n = 6)<br>**600 s ITI** (n = 19)	Conditioning time before behavior emergence as a function of ITI (experiments)	Linear regression of 30 s to 600 s data	time_before_behavior = **(-0.374)***ITI + **6025**<br>R2 = 0.00243	p = 0.742	ns
Fig. 5	e	**30 s ITI** (n = 20)<br>**60 s ITI** (n = 20)<br>**300 s ITI** (n = 20)<br>**600 s ITI** (n = 20)	Conditioning time before behavior emergence as a function of ITI (TDRL)	Linear regression	time_before_behavior = **(17.0356)***ITI + **50.64**	See **Extended Data Fig.7A**	
Fig. 5	h	**30 s ITI** (n = 20)<br>**60 s ITI** (n = 20)<br>**300 s ITI** (n = 20)<br>**600 s ITI** (n = 20)	Conditioning time before behavior emergence as a function of ITI (SOP)	Linear regression	time_before_behavior = **(14.3674)***ITI + **1169**	See **Extended Data Fig.7A**	
Fig. 5	k	**30 s ITI** (n = 20)<br>**60 s ITI** (n = 20)<br>**300 s ITI** (n = 20)<br>**600 s ITI** (n = 20)	Conditioning time before behavior emergence as a function of ITI (ANCCR)	Linear regression	time_before_behavior = **(0.5933)***ITI + **5570**	See **Extended Data Fig.7A**	
Fig. 6	c	**60 s ITI** (n = 19)<br>**60 s ITI-few** (n = 18)	change in lick rate to cue, trials 36-40	Welch's t-test	t (31.57) = 0.395	p > 0.9999<br>[0.695]	ns
Fig. 6	c	**600 s ITI** (n = 19)<br>**60 s ITI-few** (n = 18)	change in lick rate to cue, trials 36-40	Welch's t-test	t (34.71) = -7.28	$p = 3.76 \times 10^{-7}$<br>$[1.73 \times 10^{-8}]$	****

Fig. 6	f	**60 s ITI** (n = 19) **60 s ITI-few w/ context** (n = 6)	change in lick rate to cue, trials 36-40	Welch's t-test	t (15.29) = -1.06	p > 0.9999 [0.306]	ns
Fig. 6	f	**600 s ITI** (n = 19) **60 s ITI-few w/ context** (n = 6)	change in lick rate to cue, trials 36-40	Welch's t-test	t (11.39) = -4.34	p = 0.00593 [0.00109]	**
Fig. 6	i	**60 s ITI** (n = 19) **60 s ITI w/CS-** (n = 6)	change in lick rate to cue, trials 36-40	Welch's t-test	t (11.46) = -6.38	p = 0.000469 [4.32 x 10-5]	***
Fig. 6	i	**600 s ITI** (n = 19) **60 s ITI w/CS-** (n = 6)	change in lick rate to cue, trials 36-40	Welch's t-test	t (8.83) = -2.26	p = 0.22 [0.0506]	ns
Fig. 6	l	**60 s ITI** (n = 19) **600 s ITI w/ bgd milk** (n = 6)	change in lick rate to cue, trials 36-40	Welch's t-test	t (14.27) = -5.22	p = 0.000878 [0.000121]	***
Fig. 6	l	**600 s ITI** (n = 19) **600 s ITI w/ bgd milk** (n = 6)	change in lick rate to cue, trials 36-40	Welch's t-test	t (10.66) = -0.47	p > 0.9999 [0.644]	ns
Fig. 7	c	**60 s ITI - 50%** (n = 8) **60 s ITI** (n = 17)	number of rewards to learn	Welch's t-test	t (22.22) = 6.43	p = 1.70 x 10^{-6}	****
Fig. 7	d	**60 s ITI - 50%** (n = 8) **60 s ITI** (n = 17)	change in lick rate to cue last 100 trials (learners only)	Welch's t-test	t (16.44) = 3.72	p = 0.00178	**
Fig. 7	e	**60 s ITI - 50%** (n = 6) **60 s ITI** (n = 5)	cue DA last 100 trials (learners only)	Welch's t-test	t (7.24) = 0.83	p = 0.43	ns
Fig. 7	j	**60 s ITI** (n = 5) **60 s ITI - 10%** (n = 9)	trials to DA learning	Mann–Whitney U test	U = 2.5	p = 0.0179 [0.008938]	*
Fig. 7	j	**600 s ITI** (n = 7) **60 s ITI - 10%** (n = 9)	trials to DA learning	Mann–Whitney U test	U = 13.0	p = 0.0991 [0.04953]	ns

Fig. 8	d	**60 s ITI - 50%** (n = 6)	Cue DA response (60 s ITI-50%) sigmoid fit	Sigmoid fit	$y = 0.607 / (1 + e^{(-0.0271*(x + 0.352))}) -0.343$	n/a	
Fig. 8	d	**60 s ITI - 50%** (n = 6)	Omission DA response (60 s ITI-50%) sigmoid fit	Sigmoid fit	$y = -0.196 / (1 + e^{(-0.0263*(x - 182.68))}) + 0.0122$	n/a	
Fig. 8	f	**60 s ITI - 50%** (n = 6)	trials to learn: DA cue vs. omission response	paired t-test	t (5) = -5.35	p = 0.00306	**
Ext. Data Fig. 1	b	**60 s ITI** (n = 19) **600 s ITI** (n = 19)	change in lick rate to cue, trials 36-40	Welch's t-test	t (33.9) = -5.59	$p = 2.99 \times 10^{-6}$	****
Ext. Data Fig. 1	d	**60 s ITI** (n = 19) **600 s ITI** (n = 19)	prop of trials with >1 licks to cue, trials 36 - 40	Welch's t-test	t (30.66) = -8.72	$p = 8.34 \times 10^{-10}$	****
Ext. Data Fig. 2	d	**60 s ITI** (n = 17) **600 s ITI** (n = 19)	number of trials to learn (80% of max distance from diagonal)	Welch's t-test	t (16.36) = 12.46	$p = 9.06 \times 10^{-10}$	****
Ext. Data Fig. 2	d	**60 s ITI** (n = 17) **600 s ITI** (n = 19)	number of trials to learn (85% of max distance from diagonal)	Welch's t-test	t (16.36) = 12.49	$p = 8.79 \times 10^{-10}$	****
Ext. Data Fig. 2	d	**60 s ITI** (n = 17) **600 s ITI** (n = 19)	number of trials to learn (90% of max distance from diagonal)	Welch's t-test	t (16.27) = 11.57	$p = 2.88 \times 10^{-9}$	****
Ext. Data Fig. 2	d	**60 s ITI** (n = 17) **600 s ITI** (n = 19)	number of trials to learn (95% of max distance from diagonal)	Welch's t-test	t (16.3) = 11.69	$p = 2.45 \times 10^{-9}$	****
Ext. Data Fig. 2	d	**60 s ITI** (n = 17) **600 s ITI** (n = 19)	number of trials to learn (100% of max distance from diagonal)	Welch's t-test	t (16.26) = 13.13	$p = 4.51 \times 10^{-10}$	****
Ext. Data Fig. 2	g	**60 s ITI** (n = 19) **600 s ITI** (n = 19)	prop. of trials with >1 licks to cue, last 10 or 100 trials	Welch's t-test	t (23.38) = -1.72	p = 0.098	ns
Ext. Data Fig. 2	h	**60 s ITI** (n = 17) **600 s ITI** (n = 19)	abruptness of change at learning	Welch's t-test	t (33.02) = -0.040	p = 0.97	ns
Ext. Data Fig. 5	c	**45 s ISI** (n = 8) **135 s ISI** (n = 7)	trials to learn	Welch's t-test	t (7.78) = -3.41	p = 0.00954	**
Ext. Data Fig. 5	d	**45 s ISI** (n = 8) **135 s ISI** (n = 7)	total conditioning time before cue freezing	Welch's t-test	t (11.46) = 0.035	p = 0.97	ns
Ext. Data Fig. 5	j	**45 s ISI** (n = 7) **135 s ISI** (n = 6)	trials to DA learning	Welch's t-test	t (9.45) = -4.24	p = 0.00194	**
Ext. Data Fig. 5	k	**45 s ISI** (n = 7) **135 s ISI** (n = 6)	total conditioning time before cue DA	Welch's t-test	t (9.65) = -0.64	p = 0.54	ns

Ext. Data Fig. 6	b	**30 s ITI** (n = 6) **300 s ITI** (n = 6)	change in lick rate to cue, trials 71 - 80	Welch's t-test	t (9.33) = 4.88	p = 0.000777	***
Ext. Data Fig. 6	d	**30 s ITI** (n = 6) **300 s ITI** (n = 6)	change in lick rate to cue, last 20 or 200 trials	Welch's t-test	t (9.0) = 0.72	p = 0.49	ns
Ext. Data Fig. 6	e	**60 s and 600 s ITI** (n = 2 means)	mean trials to learn DA as a function of IRI	linear regression	log(trials_to_learn_DA) = **(-1.0359)**log(IRI) + **3.4338**	n/a	
Ext. Data Fig. 6	g	**600 s ITI** (n = 19) **3600 s ITI** (n = 5)	number of trials to learn	Welch's t-test	t (21.78) = 8.01	p = 6.27 x 10^{-8}	****
Ext. Data Fig. 6	j	**600 s ITI** (n = 7) **3600 s ITI** (n = 5)	trials to DA learning	Welch's t-test	t (9.98) = 3.23	p = 0.00898	**
Ext. Data Fig. 7	a	**Experimental** (n = 47) **TDRL** (n = 80)	Comparison of regression slopes: total conditioning time before cue licking vs ITI (30 – 600 s) experimental vs TDRL	t-test	t (123) = -15.39	p ≈ 0 [≈ 0]	****
Ext. Data Fig. 7	a	**experimental** (n = 47) **SOP** (n = 80)	Comparison of regression slopes: total conditioning time before cue licking vs ITI (30 – 600 s) experimental vs SOP	t-test	t (123) = -13.00	p ≈ 0 [≈ 0]	****
Ext. Data Fig. 7	a	**experimental** (n = 47) **ANCCR** (n = 80)	Comparison of regression slopes: total conditioning time before cue licking vs ITI (30 – 600 s) experimental vs ANCCR	t-test	t (123) = -0.849	p = 0. 3973 [0.3973]	ns
Ext. Data Fig. 7	a	**experimental** (n = 47) **TDRL alpha scaling** (n = 80)	Comparison of regression slopes: total conditioning time before cue licking vs ITI (30 – 600 s) experimental vs TDRL w/ alpha scaling	t-test	t (123) = -2.67	p = 0.0108 [0.008638]	*
Ext. Data Fig. 7	a	**experimental** (n = 47) **SOP (unconstrained)** (n = 80)	Comparison of regression slopes: total conditioning time before cue licking vs ITI (30 – 600 s) experimental vs SOP w/o pd1>pd2 constraint	t-test	t (123) = -7.98	p = 1.44 x 10^{-12} [8.67 x 10^{-13}]	****
Ext. Data Fig. 7	b	**30 s ITI** (n = 20) **60 s ITI** (n = 20) **300 s ITI** (n = 20) **600 s ITI** (n = 20)	<u>TDRL simulation (best fit to behavior): Akaike information criterion (AIC) model weight</u>	w/ no parameters k = 0	AIC = 445.53 Rel. weight: 4.41 x 10^{-22}	n/a	
				w/ all model parameters	AIC = 461.53		

		**3600 s ITI** (n = 20)		k = 8	Rel. weight: 2.20×10^{-23}		
Ext. Data Fig. 7	c	**30 s ITI** (n = 20)<br>**60 s ITI** (n = 20)<br>**300 s ITI** (n = 20)<br>**600 s ITI** (n = 20)<br>**3600 s ITI** (n = 20)	TDRL simulation (best fit to behavior with extra trials): Akaike information criterion (AIC) model weight	w/ no parameters<br>k = 0	AIC = 438.11<br>Rel. weight: 1.80×10^{-20}	n/a	
				w/ all model parameters<br>k = 8	AIC = 454.11<br>Rel. weight: 8.95×10^{-22}		
Ext. Data Fig. 7	c	**30 s ITI** (n = 20)<br>**60 s ITI** (n = 20)<br>**300 s ITI** (n = 20)<br>**600 s ITI** (n = 20)<br>**3600 s ITI** (n = 20)	TDRL simulation: RPE (cue) asymptotes	Kruskal-Wallis H-test	H (4) = 89.52	$p = 1.66 \times 10^{-18}$	****
Ext. Data Fig. 7	c	**60 s ITI** (n = 20)<br>**600 s ITI** (n = 20)	TDRL simulation: RPE (cue) asymptote (post-test)	Mann–Whitney U test	U = 0	$p = 2.04 \times 10^{-7}$<br>[6.80×10^{-8}]	****
Ext. Data Fig. 7	c	**60 s ITI** (n = 20)<br>**3600 s ITI** (n = 20)	TDRL simulation: RPE (cue) asymptote (post-test)	Mann–Whitney U test	U = 0	$p = 2.04 \times 10^{-7}$<br>[6.80×10^{-8}]	****
Ext. Data Fig. 7	c	**600 s ITI** (n = 20)<br>**3600 s ITI** (n = 20)	TDRL simulation: RPE (cue) asymptote (post-test)	Mann–Whitney U test	U = 140	p = 0.323<br>[0.108]	ns
Ext. Data Fig. 7	d	**30 s ITI** (n = 20)<br>**60 s ITI** (n = 20)<br>**300 s ITI** (n = 20)<br>**600 s ITI** (n = 20)<br>**3600 s ITI** (n = 20)	TDRL simulation (alpha scaled by IRI): Akaike information criterion (AIC) model weight	w/ no parameters<br>k = 0	AIC = 352.67<br>Rel. weight: 0.0643	n/a	
				w/ all model parameters<br>k = 8	AIC = 368.67<br>Rel. weight: 0.0032		
Ext. Data Fig. 7	d	**30 s ITI** (n = 20)<br>**60 s ITI** (n = 20)<br>**300 s ITI** (n = 20)<br>**600 s ITI** (n = 20)	Conditioning time before cue licking as a function of ITI (TDRL [alpha scaled by IRI])	linear regression	time_before_behavior = **(2.652)***ITI + **5132**	See **Ext. Data Fig. 7a**	

Ext. Data Fig. 7	d	**30 s ITI** (n = 20)<br>**60 s ITI** (n = 20)<br>**300 s ITI** (n = 20)<br>**600 s ITI** (n = 20)<br>**3600 s ITI** (n = 20)	TDRL simulation: RPE (cue) asymptotes	Kruskal-Wallis H-test	H (4) = 96.59	p = 5.23 x 10^{-20}	****
Ext. Data Fig. 7	d	**60 s ITI** (n = 20)<br>**600 s ITI** (n = 20)	TDRL simulation: RPE (cue) asymptote (post-test)	Mann–Whitney U test	U = 400	p = 2.40 x 10^{-8}<br>[8.01 x 10^{-9}]	****
Ext. Data Fig. 7	d	**60 s ITI** (n = 20)<br>**3600 s ITI** (n = 20)	TDRL simulation: RPE (cue) asymptote (post-test)	Mann–Whitney U test	U = 400	p = 2.40 x 10^{-8}<br>[8.01 x 10^{-9}]	****
Ext. Data Fig. 7	d	**600 s ITI** (n = 20)<br>**3600 s ITI** (n = 20)	TDRL simulation: RPE (cue) asymptote (post-test)	Mann–Whitney U test	U = 0	p = 1.40 x 10^{-9}<br>[4.68 x 10^{-10}]	****
Ext. Data Fig. 7	e	**30 s ITI** (n = 20)<br>**60 s ITI** (n = 20)<br>**300 s ITI** (n = 20)<br>**600 s ITI** (n = 20)<br>**3600 s ITI** (n = 20)	<u>SOP simulation (best fit to behavior): Akaike information criterion (AIC) model weight</u>	w/ no parameters<br>k = 0<br>w/ all model parameters<br>k = 12	AIC = 428.77<br>Rel. weight: 1.92 x 10^{-18}<br>AIC = 452.77<br>Rel. weight: 1.75 x 10^{-21}	n/a	
Ext. Data Fig. 7	f	**30 s ITI** (n = 20)<br>**60 s ITI** (n = 20)<br>**300 s ITI** (n = 20)<br>**600 s ITI** (n = 20)<br>**3600 s ITI** (n = 20)	<u>SOP simulation (best fit to behavior without pd1>pd2 constraint): Akaike information criterion (AIC) model weight</u>	w/ no parameters<br>k = 0<br>w/ all model parameters<br>k = 12	AIC = 373.50<br>Rel. weight: 1.94 x 10^{-6}<br>AIC = 397.50<br>Rel. weight: 1.77 x 10^{-9}	n/a	
Ext. Data Fig. 7	f	**30 s ITI** (n = 20)<br>**60 s ITI** (n = 20)<br>**300 s ITI** (n = 20)<br>**600 s ITI** (n = 20)	Conditioning time before cue licking as a function of ITI (SOP [no pd1>pd2 constraint]])	linear regression	time_before_behavior = **(8.998)***ITI + **3733**	See **Ext. Data Fig. 7a**	

Ext. Data Fig. 7	g	**30 s ITI** (n = 20) **60 s ITI** (n = 20) **300 s ITI** (n = 20) **600 s ITI** (n = 20) **3600 s ITI** (n = 20)	<u>ANCCR simulation (best fit to behavior): Akaike information criterion (AIC) model weight</u>	w/ no parameters k = 0	AIC = 347.18<br>Rel. weight: 1	n/a	
				w/ all model parameters k = 5	AIC = 357.18<br>Rel. weight: 1		
Ext. Data Fig. 7	g	**30 s ITI** (n = 20) **60 s ITI** (n = 20) **300 s ITI** (n = 20) **600 s ITI** (n = 20) **3600 s ITI** (n = 20)	ANCCR simulation: ANCCR (cue) asymptotes	Kruskal-Wallis H-test	H (4) = 95.05	$p = 1.11 \times 10^{-19}$	****
Ext. Data Fig. 7	g	**60 s ITI** (n = 20) **600 s ITI** (n = 20)	ANCCR simulation: ANCCR asymptote (post-test)	Mann–Whitney U test	U = 0	$p = 2.04 \times 10^{-7}$<br>[6.80×10^{-8}]	****
Ext. Data Fig. 7	g	**60 s ITI** (n = 20) **3600 s ITI** (n = 20)	ANCCR simulation: ANCCR asymptote (post-test)	Mann–Whitney U test	U = 0	$p = 2.04 \times 10^{-7}$<br>[6.80×10^{-8}]	****
Ext. Data Fig. 7	g	**600 s ITI** (n = 20) **3600 s ITI** (n = 20)	ANCCR simulation: ANCCR asymptote (post-test)	Mann–Whitney U test	U = 0	$p = 2.04 \times 10^{-7}$<br>[6.80×10^{-8}]	****
Ext. Data Fig. 8	b	**60 s ITI** (n = 6) **60 s ITI-few** (n = 6)	cue DA, trials 36 - 40	Welch's t-test	t (9.76) = -1.50	p = 0.330<br>[0.165]	ns
Ext. Data Fig. 8	b	**600 s ITI** (n = 7) **60 s ITI-few** (n = 6)	cue DA, trials 36 - 40	Welch's t-test	t (10.98) = -2.99	p = 0.0246<br>[0.0123]	*
Ext. Data Fig. 8	f	**60 s ITI** (n = 19)	60 s ITI - reward bout duration first 6 vs. last 6 trials per session	paired t-test	t (18) = -0.0034	p = 0.997	ns
Ext. Data Fig. 8	f	**30 s ITI** (n = 5)	30 s ITI - reward bout duration first 6 vs. last 6 trials per session	paired t-test	t (5) = -0.62	p = 0.564	ns
Ext. Data Fig. 8	i	**30 s, 60 s, 300 s, 600 s, and 3600 s ITI** (n = 5 means)	mean lick rate during ITI (before learning) as a function of IRI	linear regression	log(ITI_lick_rate) = **(-0.6129)**log(IRI) + **0.7577**<br>$R^2 = 0.9960$	p = 0.000108	***

Ext. Data Fig. 8	j	**30 s, 60 s, 300 s, 600 s, and 3600 s ITI** (n = 5 means)	mean trials to learn as a function of mean lick rate during ITI (before learning)	linear regression	log(learned_trial) = (**1.4078**)log(ITI_lick_rate) + **2.421**<br><br>$R^2 = 0.970$	p = 0.00221	**
Ext. Data Fig. 8	k	**60 s ITI** (n = 17)	learned trial vs. lick rate during ITI (before learning)	linear regression	log(learned_trial) = **(-0.1260)**log(ITI_lick_rate) + **1.8712**<br><br>$R^2 = 0.361$	p = 0.0106	*
Ext. Data Fig. 8	k	**300 s ITI** (n = 6)	learned trial vs. lick rate during ITI (before learning)	linear regression	log(learned_trial) = **(-0.4865)**log(ITI_lick_rate) + **0.7340**<br><br>$R^2 = 0.734$	p = 0.0292	*
Ext. Data Fig. 8	k	**600 s ITI** (n = 18)	learned trial vs. lick rate during ITI (before learning)	linear regression	log(learned_trial) = **(-0.2310)**log(ITI_lick_rate) + **0.6900**<br><br>$R^2 = 0.313$	p = 0.0158	*
Ext. Data Fig. 8	n/a	**30 s ITI** (n = 5)	learned trial vs. lick rate during ITI (before learning)	linear regression	log(learned_trial) = (**-0.1866)**log(ITI_lick_rate) + **2.165**<br><br>$R^2 = 0.0951$	p = 0.614	ns
Ext. Data Fig. 8	n/a	**3600 s ITI** (n = 5)	learned trial vs. lick rate during ITI (before learning)	linear regression	log(learned_trial) = **(-0.1105)**log(ITI_lick_rate) + **0.3890**<br><br>$R^2 = 0.190$	p = 0.462	ns
Ext. Data Fig. 9	b	**60 s ITI** (n = 17) **60 s ITI w/ CS-** (n = 6)	number of trials to learn	Welch's t-test	t (16.65) = 12.53	$p = 1.34 \times 10^{-9}$<br><br>$[6.70 \times 10^{-10}]$	****
Ext. Data Fig. 9	b	**600 s ITI** (n = 19) **60 s ITI w/ CS-** (n = 6)	number of trials to learn	Welch's t-test	t (9.22) = -0.19	p > 0.9999<br><br>[0.857]	ns
Ext. Data Fig. 9	f	**600 s ITI w/bgd milk** (n = 6)	number of trials to learn observed different from predicted (identity specific IRI: 8.49)	one sample t-test	t(5) = 6.88	p = 0.00198 [0.00099]	**
Ext. Data Fig. 9	f	**600 s ITI w/bgd milk** (n = 6)	number of trials to learn observed different from predicted (general IRI: 26.80)	one sample t-test	t(5) = -25.93	$p = 3.19 \times 10^{-6}$<br><br>$[1.60 \times 10^{-6}]$	****
Ext. Data Fig. 9	g	**600 s ITI** (n = 19) **600 s ITI w/bgd milk** (n = 6)	number of trials to learn	Welch's t-test	t (16.96) = -4.33	p = 0.000461	***

Ext. Data Fig. 10	a	**60 s ITI - 50%** (n = 8)	number of trials to learn observed different from predicted (by ICI: 91.24)	one sample t-test	t (7) = -13.08	p = 7.13 x 10^{-6} [3.56 x 10-6]	****
Ext. Data Fig. 10	a	**60 s ITI - 50%** (n = 8)	number of trials to learn observed different from predicted (by IRI: 43.78)	one sample t-test	t (7) = -0.34	p > 0.9999 [0.74]	ns
Ext. Data Fig. 10	b	**60 s ITI - 50%** (n = 6)	number of trials to learn DA observed different from predicted (by ICI: 36.4)	one sample t-test	t (5) = -5.37	p = 0.006046 [0.003023]	**
Ext. Data Fig. 10	b	**60 s ITI - 50%** (n = 6)	number of trials to learn DA observed different from predicted (by IRI: 17.75)	one sample t-test	t (5) = 1.58	p =0.35 [0.174]	ns
Ext. Data Fig. 10	c	**60 s ITI** (n = 5) **600 s ITI (n = 6)**	**rewards** to DA learning	Welch's t-test	t (5.07) = 1.84	p = 0.124	ns
Ext. Data Fig. 10	d	**60 s ITI** (n = 17) **60 s ITI - 50%** (n = 8)	number of **trials** to learn	Welch's t-test	t (20.41) = 0.79	p = 0.441	ns
Ext. Data Fig. 10	e	**60 s ITI** (n = 5) **60 s ITI - 50%** (n = 6)	**trials** to DA learning	Welch's t-test	t (8.45) = -1.02	p = 0.335	ns
Ext. Data Fig. 10	k	**60 s ITI** (n = 17) **60 s ITI - 10%** (n = 9)	number of trials to learn	Mann–Whitney U test	U = 0	p = 0.000084 [0.000042]	****
Ext. Data Fig. 10	k	**600 s ITI** (n = 19) **60 s ITI - 10%** (n = 9)	number of trials to learn	Mann–Whitney U test	U = 29.0	p = 0.0114 [0.00568]	*
