# Supplementary material for: Duration between rewards controls the rate of behavioral and dopaminergic learning
Source: Nat Neurosci. 2026 Feb 12;29(4):825–39. doi: 10.1038/s41593-026-02206-2 (PMC13035403; doi:10.1038/s41593-026-02206-2)
Supplement: Supplementary file 2 — Reporting Summary [file 41593_2026_2206_MOESM2_ESM.pdf]

nature portfolio

Corresponding author(s): Vijay Mohan K Namboodiri

Last updated by author(s): Nov 24, 2025

Reporting Summary

Nature Portfolio wishes to improve the reproducibility of the work that we publish. This form provides structure for consistency and transparency in reporting. For further information on Nature Portfolio policies, see our Editorial Policies and the Editorial Policy Checklist.

Statistics

For all statistical analyses, confirm that the following items are present in the figure legend, table legend, main text, or Methods section.

n/a	Confirmed	
☐	☒	The exact sample size (*n*) for each experimental group/condition, given as a discrete number and unit of measurement
☐	☒	A statement on whether measurements were taken from distinct samples or whether the same sample was measured repeatedly
☐	☒	The statistical test(s) used AND whether they are one- or two-sided<br>*Only common tests should be described solely by name; describe more complex techniques in the Methods section.*
☒	☐	A description of all covariates tested
☐	☒	A description of any assumptions or corrections, such as tests of normality and adjustment for multiple comparisons
☐	☒	A full description of the statistical parameters including central tendency (e.g. means) or other basic estimates (e.g. regression coefficient) AND variation (e.g. standard deviation) or associated estimates of uncertainty (e.g. confidence intervals)
☐	☒	For null hypothesis testing, the test statistic (e.g. *F*, *t*, *r*) with confidence intervals, effect sizes, degrees of freedom and *P* value noted<br>*Give P values as exact values whenever suitable.*
☒	☐	For Bayesian analysis, information on the choice of priors and Markov chain Monte Carlo settings
☒	☐	For hierarchical and complex designs, identification of the appropriate level for tests and full reporting of outcomes
☐	☒	Estimates of effect sizes (e.g. Cohen's *d*, Pearson's *r*), indicating how they were calculated

Our web collection on statistics for biologists contains articles on many of the points above.

Software and code

Policy information about availability of computer code

Data collection	Cue reward conditioning behavioral data were collected using custom MATLAB scripts (MATLAB 2020b) based on our prior publication (https://github.com/namboodirilab/B-CALM). Fiber photometry data were collected through a Doric Fiber Photometry Console running Doric Neuroscience Studio v5.4/v6.4 or a pyPhotometry. Aversive (fear) conditioning behavior was controlled by MED-PC software. Aversive conditioning photometry data was collected through a RWD R821 Tricolor MultiChannel 51 Fiber Photometry System running OFRS Software (version V2.0.0.33169). Motion and freezing behavior for cue-shock conditioning was generated from video files using ezTrack: (https://github.com/denisecailab/ezTrack)
Data analysis	All code for generating figures, analyzing data, and running simulations can be found at: https://github.com/namboodirilab/duration_between_rewards_controls_learning_rate . Statistical analyses were performed in Python 3.12 using either the scipy.stats (v1.12.016.2) or Pingouin 72 (v0.5.5). Model simulations were run in MATLAB 2021b.

For manuscripts utilizing custom algorithms or software that are central to the research but not yet described in published literature, software must be made available to editors and reviewers. We strongly encourage code deposition in a community repository (e.g. GitHub). See the Nature Portfolio guidelines for submitting code & software for further information.

Data

Policy information about availability of data

All manuscripts must include a data availability statement. This statement should provide the following information, where applicable:

- Accession codes, unique identifiers, or web links for publicly available datasets
- A description of any restrictions on data availability
- For clinical datasets or third party data, please ensure that the statement adheres to our policy

Data underlying this study is publicly available on DANDI at https://dandiarchive.org/dandiset/001632

Research involving human participants, their data, or biological material

Policy information about studies with human participants or human data. See also policy information about sex, gender (identity/presentation), and sexual orientation and race, ethnicity and racism.

Reporting on sex and gender	n/a
Reporting on race, ethnicity, or other socially relevant groupings	n/a
Population characteristics	n/a
Recruitment	n/a
Ethics oversight	n/a

Note that full information on the approval of the study protocol must also be provided in the manuscript.

Field-specific reporting

Please select the one below that is the best fit for your research. If you are not sure, read the appropriate sections before making your selection.

☒ Life sciences ☐ Behavioural & social sciences ☐ Ecological, evolutionary & environmental sciences

For a reference copy of the document with all sections, see nature.com/documents/nr-reporting-summary-flat.pdf

Life sciences study design

All studies must disclose on these points even when the disclosure is negative.

Sample size	No statistical test was used to predetermine sample sizes. Sample sizes were chosen based on n's in similar in published studies.
Data exclusions	One 60 s ITI mouse implanted with an optic fiber was excluded from all dopamine analyses due to a missed fiber placement (Extended Data Fig. 3). One additional 60 s ITI mouse that was implanted with an optic fiber failed to learn the cue-reward association (Extended Data Fig. 2c) and was excluded from dopamine analyses. Two 60 s ITI-50% mice implanted with dopamine fibers failed to learn the cue-reward association (Fig. 7b and Extended Data Fig. 10f) and were excluded from comparisons against 60 s ITI mice and omission dip calculations (Fig. 6c,n-p and 17 Extended Data Fig. 10). One outlier 60 s ITI-10% mouse was excluded from analysis due to consistent negative dopamine dips to the cue precluding our ability to detect the point at which the cue evoked increase emerges (Extended Data Fig. 10j). Two cue-shock conditioned mice (one 45 s ISI and one 135 s ISI) were excluded from dopamine analyses due to the absence of a consistent dip during the cue throughout conditioning (Extended Data Fig. 5l). All exclusions are noted in the manuscript and these animals' data are shown in the supplement.
Replication	While no complete replication of the findings was performed, data for each experiment were acquired through multiple, serial cohorts of animals run across multiple months or years (eg. 60 s and 600 s ITI groups each consisted of 4 cohorts of 2-6 mice each conditioned over the course of 10 months). Groups run across time displayed qualitatively similar behavior.
Randomization	Cagemates were randomly assigned to experimental groups following surgery. Behavior only mice were group housed with mice from at least one other experimental group.
Blinding	Blinding was not possible during data acquisition because experimenter had to use specific conditioning protocols based on grouping. Experimenters were not blind to groups during data analysis, but were blind to group identity during histology for fiber placement verification.

Reporting for specific materials, systems and methods

We require information from authors about some types of materials, experimental systems and methods used in many studies. Here, indicate whether each material, system or method listed is relevant to your study. If you are not sure if a list item applies to your research, read the appropriate section before selecting a response.

Materials & experimental systems

n/a	Involved in the study
☒	☐ Antibodies
☒	☐ Eukaryotic cell lines
☒	☐ Palaeontology and archaeology
☐	☒ Animals and other organisms
☒	☐ Clinical data
☒	☐ Dual use research of concern
☒	☐ Plants

Methods

n/a	Involved in the study
☒	☐ ChIP-seq
☒	☐ Flow cytometry
☒	☐ MRI-based neuroimaging

Animals and other research organisms

Policy information about studies involving animals; ARRIVE guidelines recommended for reporting animal research, and Sex and Gender in Research

Laboratory animals	Adult wild-type male and female C57BL/6J mice (JAX; RRID:IMSR_JAX:000664) were used in this study. Mice were >8 weeks of age at time of surgery (median: 9.5 weeks) and >11 weeks at time of experiments (median: 13 weeks). Animal's were maintained on a reverse 12:12 lightcycle with lights off from 8am to 8pm. All experiments were run in the dark cycle. Animal holding room was maintained at ~23-24C with 40-50% humidity.
Wild animals	This study did not involve wild animals.
Reporting on sex	Both male and female mice were used in roughly equal numbers across conditions. The focus of this study was not on sex differences. While small sample sizes preclude a rigorous analysis of sex differences across all conditions tested, no significant difference in "trials to learn" was found between females and males in either 60 s or 600 s ITI groups (Figure 1), the two conditions best powered to detect differences. Thus, sexes were pooled for all analyses.
Field-collected samples	This study did not involve samples collected from the field.
Ethics oversight	All animal care and experimental procedures were approved by the University of California San Francisco Institutional Animal Care & Use Committee (UCSF IACUC).

Note that full information on the approval of the study protocol must also be provided in the manuscript.

Plants

Seed stocks	*Report on the source of all seed stocks or other plant material used. If applicable, state the seed stock centre and catalogue number. If plant specimens were collected from the field, describe the collection location, date and sampling procedures.*
Novel plant genotypes	*Describe the methods by which all novel plant genotypes were produced. This includes those generated by transgenic approaches, gene editing, chemical/radiation-based mutagenesis and hybridization. For transgenic lines, describe the transformation method, the number of independent lines analyzed and the generation upon which experiments were performed. For gene-edited lines, describe the editor used, the endogenous sequence targeted for editing, the targeting guide RNA sequence (if applicable) and how the editor was applied.*
Authentication	*Describe any authentication procedures for each seed stock used or novel genotype generated. Describe any experiments used to assess the effect of a mutation and, where applicable, how potential secondary effects (e.g. second site T-DNA insertions, mosiacism, off-target gene editing) were examined.*
